# Siglec15 shapes a non-inflamed tumor microenvironment and predicts the molecular subtype in bladder cancer

**DOI:** 10.7150/thno.53649

**Published:** 2021-01-01

**Authors:** Jiao Hu, Anze Yu, Belaydi Othmane, Dongxu Qiu, Huihuang Li, Chao Li, Peihua Liu, Wenbiao Ren, Minfeng Chen, Guanghui Gong, Xi Guo, Huihui Zhang, Jinbo Chen, Xiongbing Zu

**Affiliations:** 1 Department of Urology, Xiangya Hospital, Central South University, Changsha, China; 2 Immunobiology & Transplant Science Center, Houston Methodist Research Institute, Texas Medical Center, Houston, TX 77030, USA; 3 Department of Pathology, Xiangya Hospital, Central South University, Changsha, China; 4 Department of Urology, Hunan Provincial People's Hospital, Changsha, China; 5 Department of Urology, the First Affiliated Hospital of the University of South China, Hengyang, China

**Keywords:** Siglec15, Bladder cancer, Immunotherapy, Molecular subtype, Tumor microenvironment

## Abstract

**Rationale:** Siglec15 is an emerging target for normalization cancer immunotherapy. However, pan-cancer anti-Siglec15 treatment is not yet validated and the potential role of Siglec15 in bladder cancer (BLCA) remains elusive.

**Methods:** We comprehensively evaluated the expression pattern and immunological role of Siglec15 using pan-cancer analysis based on RNA sequencing data obtained from The Cancer Genome Atlas. We then systematically correlated Siglec15 with immunological characteristics in the BLCA tumor microenvironment (TME), including immunomodulators, cancer immunity cycles, tumor-infiltrating immune cells (TIICs), immune checkpoints, and T cell inflamed score. We also analyzed the role of Siglec15 in predicting the molecular subtype and the response to several treatment options in BLCA. Our results were validated in several public cohorts as well as our BLCA tumor microarray cohort, the Xiangya cohort. We developed an immune risk score (IRS), validated it, and tested its ability to predict the prognosis and response to cancer immunotherapy.

**Results:** We found that Siglec15 was specifically overexpressed in the TME of various cancers. We hypothesize that Siglec15 designs a non-inflamed TME in BLCA based on the evidence that Siglec15 negatively correlated with immunomodulators, TIICs, cancer immunity cycles, immune checkpoints, and T cell inflamed score. Bladder cancer with high Siglec15 expression was not sensitive to cancer immunotherapy, but exhibited a higher incidence of hyperprogression. High Siglec15 levels indicated a luminal subtype of BLCA characterized by lower immune infiltration, lower response to cancer immunotherapy and neoadjuvant chemotherapy, but higher response to anti-angiogenic therapy and targeted therapies such as blocking Siglec15, β-catenin, PPAR-γ, and FGFR3 pathways. Notably, a combination of anti-Siglec15 and cancer immunotherapy may be a more effective strategy than monotherapy. IRS can accurately predict the prognosis and response to cancer immunotherapy.

**Conclusions:** Anti-Siglec15 immunotherapy might be suitable for BLCA treatment as Siglec15 correlates with a non-inflamed TME in BLCA. Siglec15 could also predict the molecular subtype and the response to several treatment options.

## Introduction

Bladder cancer (BLCA) is the second most common urinary cancer 1. Despite neoadjuvant and adjuvant chemotherapy, the outcome for metastatic BLCA is poor 2. Cancer immunotherapy, including immune checkpoint blockade (ICB), has achieved promising survival benefits for advanced BLCA 3, 4. Although BLCA is an immunogenic cancer characterized by high tumor mutation burden (TMB) and neoantigens 5, only a small number of patients respond to ICB because of primary or secondary mechanisms of resistance to it 3, 4. An inflamed tumor microenvironment (TME) in conjunction with pre-existing anticancer immunity is necessary, but not sufficient, for the success of ICB 6-9. ICB inhibits tumor growth by re-invigorating tumor-cytotoxic T cells in TME, but does not induce their formation 10. Theoretically, molecules or pathways resulting in a non-inflamed TME will cause resistance to ICB. In BLCA, such molecules and pathways, including β-catenin, PPAR-γ, and FGFR3 pathways, have been shown to promote the formation of a non-inflamed TME by excluding the infiltration level of tumor-infiltrating immune cells (TIICs) 11-15. For patients with a non-inflamed TME, transforming it into an inflamed TME by reversing these ICB-resistant mechanisms is one of the top priorities along with promoting the recruitment of TIICs to drive tumor regression 16.

Given the substantial economic burden and toxic side effects of cancer treatments, more robust and economic biomarkers that predict the response to ICB must be explored. PD-L1 is related to the clinical response of ICB in many clinical trials, but its predictive value may be weakened by many factors 17, 18. TMB, microsatellite instability (MSI), and the molecular subtype can predict the clinical response of BLCA to ICB. However, these biomarkers are detected using complex molecular methods, which are slow and expensive 3, 4. Moreover, molecular subtypes can predict the prognosis and some therapeutic responses of BLCA 19, but their widespread clinical application has failed so far. Therefore, there is an urgent medical need for the development of faster and economical molecular subtype predictors.

Siglec15, a member of the sialic acid-binding immunoglobulin-like lectins family, is an emerging broad-spectrum target for normalization cancer immunotherapy, and is complementary to PD-L1 20, 21. Wang et al. demonstrated that Siglec15 promoted tumor growth by inhibiting the proliferation of CD8+ T cells, and Siglec15 inhibitors could relieve this immunosuppression 20. The results of a phase I clinical trial in advanced non-small cell lung cancer (NSCLC) indicated that Siglec15 inhibitors achieved a promising clinical response (NCT03665285). Currently, a phase II clinical trial is ongoing to assess this treatment's efficacy in solid tumors including NSCLC, ovarian cancer (OV), melanoma, breast cancer (BRCA), and colorectal cancer. However, it is critical to note that the potential of Siglec15 as a broad-spectrum therapeutic target was not validated in pan-cancers before initiating this phase II clinical trial. The recent progress of this phase II clinical trial in NSCLC and ovarian cancer has been slow, casting doubt on the validity of Siglec15 inhibitors in unselected cancer types (http://ir.nextcure.com/news-releases/news-release-details/nextcure-provides-interim-update-phase-2-portion-nc318). Recently, Li et al. performed a pan-cancer analysis on Siglec15 and confirmed that Siglec15 plays an immunoregulatory role in lung adenocarcinoma and may be a vital prognostic biomarker 22. However, the role of Siglec15 in BLCA should be further investigated.

In this study, we performed a pan-cancer analysis of the expression patterns and immunological role of Siglec15. We found that BLCA may be a suitable candidate for anti-Siglec15 therapy. We also report that Siglec15 promotes the development of a non-inflamed TME in BLCA, and has the potential to predict the molecular subtype of BLCA.

## Methods

**Figure 1:**
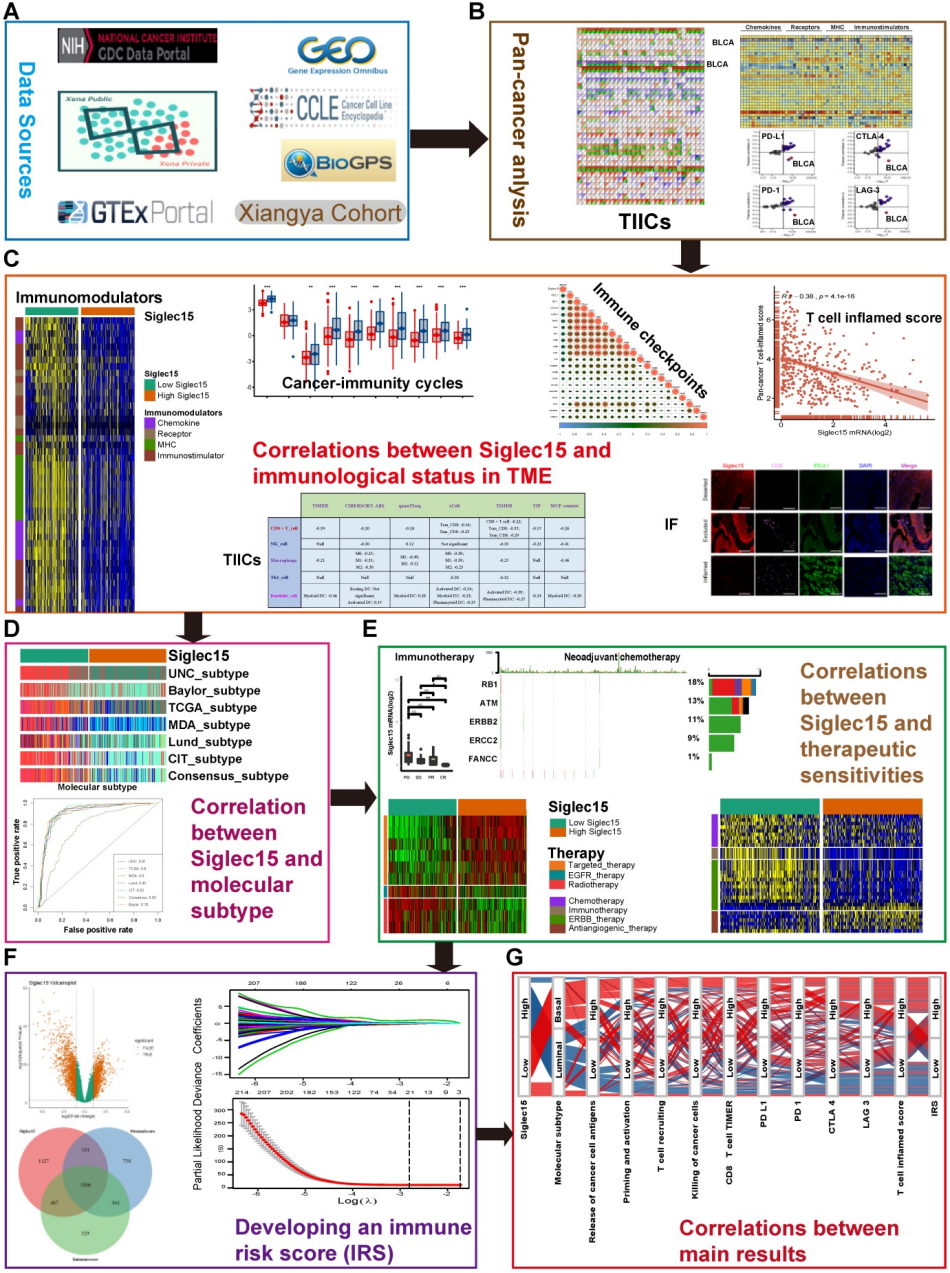
** Overview of the study design.** (A) Data sources used in this study; (B) Correlations between Siglec15 and pan-cancer immunological elements. TIICs were estimated using ssGSEA algorithm. (C) Correlation between Siglec15 and the immunological status of the tumor microenvironment in BLCA. The immunological status includes immunomodulators, steps of the cancer-immunity cycle, immune checkpoints, T cell-inflamed score, and TIICs. (D) Role of Siglec15 in predicting the molecular subtypes in BLCA. (E) Role of Siglec15 in predicting therapeutic responses to immunotherapy, neoadjuvant/adjuvant chemotherapy, targeted therapy, EGFR therapy, and radiotherapy in BLCA. (F) Development of an immune risk score; (G) Sankey plot displaying the main results in this study. TIICs: tumor-infiltrating immune cells, IF: immunofluorescence.

Figure 1 shows the workflow of this study.

### Data retrieval and preprocessing

The Cancer Genome Atlas (TCGA) data: The pan-cancer RNA sequencing (RNA-seq) data (FPKM value), somatic mutation data, and survival information were downloaded from the UCSC Xena data portal 23. Data on RNA-seq were log2 transformed, while somatic mutation data were analyzed using VarScan2 and then used to calculate TMB. MSI data were collected from the supplementary files of Bonneville's study 24. The copy number variation (CNV) data, processed using the GISTIC algorithm, were downloaded from the UCSC Xena data portal, whereas the methylation data were downloaded from the LinkedOmics data portal 25. The abbreviations for various cancer types are given in Table S1.

Gene Expression Omnibus (GEO): Eight BLCA GEO cohorts with detailed survival data were downloaded, namely GSE13507, GSE31684, GSE48075, GSE48277, GSE69795, GSE70691, GSE32894, and GSE5287. Three immunotherapy-related cohorts, GSE78220 (melanoma), GSE135222 (NSCLC), and GSE91061 (melanoma), were also downloaded. In addition, an immunotherapy cohort (PMID29301960) of renal cell carcinoma was collected from the supplementary files of Miao's study 26. Based on the Creative Commons 3.0 License, the complete expression data and detailed clinical information of the IMvigor210 cohort (a BLCA immunotherapy related cohort) were obtained from http://research-pub.Gene.com/imvigor210corebiologies/27. Detailed information about these datasets is summarized in Table S1**.**

Other data sources: Expression data of Siglec15 in normal tissues were downloaded from BioGPS data portal and Genotype-Tissue Expression (GTEx) project. Finally, expression data of Siglec15 in cancer cell lines were downloaded from the BioGPS data portal and Cancer Cell Line Encyclopedia (CCLE) project.

### Evaluation of the immunological characteristics of the TME in BLCA

Immunological characteristics of the TME in BLCA include the expression of immunomodulators, activity of the cancer immunity cycle, infiltration level of TIICs, and the expression of inhibitory immune checkpoints. We first collected information on 122 immunomodulators including MHC, receptors, chemokines, and immune stimulators from the study of Charoentong et al. (Table S2) 28. The cancer immunity cycle reflects the anticancer immune response and comprises seven steps: release of cancer cell antigens (Step 1), cancer antigen presentation (Step 2), priming and activation (Step 3), trafficking of immune cells to tumors (Step 4), infiltration of immune cells into tumors (Step 5), recognition of cancer cells by T cells (Step 6), and killing of cancer cells (Step 7) (Table S3) 29. The activities of these steps determine the fate of the tumor cells. Xu et al. evaluated the activities of these steps using a single sample gene set enrichment analysis (ssGSEA) based on the gene expression of individual samples 30. Thereafter, several algorithms were developed to calculate the infiltration level of TIICs in TME using bulk RNA-seq data. Different algorithms and marker gene sets of TIICs initiate calculation errors. To avoid these errors, we comprehensively calculated the infiltration level of TIICs using seven independent algorithms: Cibersort-ABS, MCP-counter, quanTIseq, TIMER, xCell, TIP, and TISIDB (Table S4) 30-36. We also identified the effector genes of TIICs from previous studies (Table S5). Finally, we collected 22 inhibitory immune checkpoints with therapeutic potential from Auslander's study (Table S6) 37.

Ayers et al. developed and validated a pan-cancer T cell-inflamed score, which could define pre-existing cancer immunity, as well as predict the clinical response of ICB 38. The eighteen genes included in the T cell-inflamed score algorithm and their coefficients are shown in Table S7. Here, we computed the T cell inflamed score as a weighted linear combination of the scores from the 18 genes. Hyperprogression is an adverse event associated with ICB. We summarized several predictors of hyperprogression (Table S8) 39-41. The amplification and high mRNA expression of MDM2, MDM4, DNMT3A, CCND1, FGF19, FGF4, and FGF3 are positively correlated with hyperprogression. In addition, the deletion and low mRNA expression of CDKN2A and CDKN2B are also positively correlated with hyperprogression.

To confirm the role of Siglec15 in modulating cancer immunity in BLCA, we analyzed the correlation between Siglec15 and the immunological characteristics of TME with respect to the above aspects. The findings from this study were validated in three independent external cohorts, including GSE31684, GSE32894, and IMvigor210.

### Immunohistochemistry and immunofluorescence staining of bladder cancer microarray

Sixty-three bladder cancer specimens were prepared in a tissue microarray (TMA) format. Representative tumor areas were obtained from formalin-fixed, paraffin-embedded specimens of primary cancer tissues, where 1.5-mm cores from each cancer block were arrayed. Immunohistochemistry was performed as described previously 42. Briefly, staining was performed using a CD8-specific antibody (ab4055, Abcam), an anti-PD-L1 antibody (ab213524, Abcam), and an anti-Siglec15 antibody (ab198684, Abcam) in combination with a secondary antibody [horseradish peroxidase (HRP)-conjugated goat anti-rabbit immunoglobulin G (IgG)]. For the scoring of PD-L1, we first evaluated the staining intensity of whole tumor tissue at low magnification. Samples with no staining in any cancer cells were assigned score 0, weakly stained samples scored 1, samples stained with moderate intensity scored 2, and samples with a strong intensity of staining scored 3. We also calculated the number of positive cells from five high magnification fields chosen at random as well as their mean intensities. As described above, samples with < 25 % positive expression were scored 1, samples within the expression range of 25 %-50 % scored 2, samples within the expression range of 50 %-75 % scored 3, and samples with expression ≥ 75% scored 4. The final PD-L1 expression was determined by multiplying the intensity score with the positive expression value. For CD8 and Siglec15 staining, we estimated only the percentage of cells with a strong intensity (brown staining) of membrane staining. Tumors were classified into three phenotypes based on the spatial distribution of CD8+ T cells. These included the “inflamed phenotype”: CD8+ T cells located in the tumor parenchyma; the “excluded phenotype”: CD8+ T cells located in the stroma surrounding the tumor but not in parenchyma; and the “deserted phenotype,” characterized by the absence of CD8+ T cells in both tumor parenchyma and stroma. Both excluded and deserted phenotypes can be considered as non-inflamed phenotypes. All slides were reviewed by two independent pathologists. Immunofluorescence co-staining of Siglec15, CD8, and PD-L1 was performed as described by Wang et al. 20. Briefly, co-staining for Siglec-15, PD-L1, and CD8 was performed using a sequential multiplexed immunofluorescence protocol with three antibodies used in the immunohistochemistry. Corresponding secondary antibodies used were CY3-TSA (G1222, Servicebio) for Siglec15 detection, FITC-TSA (G1223, Servicebio) for PD-L1 detection and CY5-TSA (G1224 Servicebio) for CD8 detection. Nuclei were highlighted using DAPI. Finally, we estimated the proportions of positive cells in the whole field.

### RNA sequencing of bladder cancer samples

Sixty fresh bladder cancer samples were collected from our hospital and were immediately stored in liquid nitrogen. Total RNA was extracted from the tissues using TRIzol (Invitrogen, Carlsbad, CA, USA) according to the manufacturer's instructions. Subsequently, NanoDrop and Agilent 2100 bioanalyzer (Thermo Fisher Scientific, MA, USA) were used to quantify total RNA. The mRNA library was then constructed. Total RNA was purified and fragmented into small pieces. Then, first-strand cDNA and second-strand cDNA were synthesized. The cDNA fragments were further amplified by PCR after incubating with A-tailing mix and RNA Adapter Index for end repair. The qualified double-stranded PCR products were then used to construct the final library (single-stranded circular DNA). Among the 60 samples collected, 57 qualified. Eventually, the 57 qualified bladder cancer samples (named the Xiangya cohort) were further sequenced on a BGISEQ-500 platform (BGI-Shenzhen, China). The gene expression levels were calculated using RSEM (v1.2.12).

### Real-time quantitative PCR (qPCR)

For real-time qPCR analysis, total RNA was extracted from 30 paired samples of bladder cancer and adjacent normal tissues using RNA extraction reagent (Donghuan, Shanghai, China) according to the manufacturer's instructions. cDNA was synthesized using a cDNA synthesis kit (Takara, Dalian, China). Real-time q-PCR was performed to detect the expression of Siglec15 using SYBR Green qPCR Master Mix (Junxing, Suzhou, China). Gene expression levels were normalized to the “housekeeping” gene GAPDH. Primer sequences for Siglec15 and GAPDH were as follows: Siglec15 (forward primer: 5′-TTTGAGCCAGATGAACCCCC-3′; reverse primer: 5′-CAGGGAGCTCCGAAATGGTT-3′); GAPDH (forward primer: 5′-GACAGTCAGCCGCATCTTCT-3′; reverse primer: 5′-GCGCCCAATACGACCAAATC-3′).

### Calculation of the enrichment scores of various gene signatures

A set of gene signatures positively correlated with the clinical response of an anti-PD-L1 agent (atezolizumab) in BLCA were collected from Mariathasan's study 27. Twelve bladder cancer signatures that are specific to different molecular subtypes were collected from the study performed by the Bladder Cancer Molecular Taxonomy Group 19. We also collected other therapeutic signatures, including oncogenic pathways that could shape a non-inflamed TME, targeted therapy-associated gene signatures, and gene signatures predicting radiotherapy responses (Table S9). The enrichment scores of these signatures were calculated using the GSVA R package 43. Subsequently, it was noted that the mutation statuses of several critical genes, including RB1, ATM, ERBB2, ERCC2, and FANCC, were predictors of the response to neoadjuvant chemotherapy in BLCA 44-47.

After comparing the differences in the values of the enrichment scores of therapeutic signatures and the mutation statuses of neoadjuvant chemotherapy predictors between Siglec15 groups, we could determine the role of Siglec15 in predicting the response to these therapies. Finally, the BLCA-related drug-target genes were screened using the Drugbank database (Table S10) 48.

### Prediction of the molecular subtypes in BLCA

There are several molecular subtype systems, such as CIT, Lund, MDA, TCGA, Baylor, UNC, and Consensus subtypes 19, 49-54. ConsensusMIBC and BLCAsubtyping R packages were used to determine the molecular subtypes of individuals. Thereafter, we correlated Siglec15 with different molecular subtypes and specific bladder cancer gene signatures. Based on the correlations between different molecular subtype systems depicted previously, BLCA can be classified into two major subtypes, namely basal and luminal subtypes (Table S11) 19. Receiver operating characteristic (ROC) curves were plotted to explore the predictive accuracy of Siglec15 for molecular subtypes. Moreover, the predictive accuracy of Siglec15 was validated in four external validation cohorts, including two general BLCA cohorts (GSE31684, GSE48277), one immunotherapy-related cohort (IMvigor210), and one neoadjuvant chemotherapy-related cohort (GSE70691).

### Identification of immune-related differentially expressed RNAs (DERs)

Patients were classified into various groups based on the median Siglec15 mRNA expression, immune score, and stromal score. The immune and stromal scores of BLCA were calculated using the ESTIMATE R package. The empirical Bayesian approach of the limma R package was applied to identify DERs from the RNA-seq data. The criteria for determining differential DERs were set with the adjusted P-value < 0.01, and the |log(fold change)|>1. We determined the common DERs using the VennDiagram R package. Gene Ontology (GO) and Kyoto Encyclopedia of Genes and Genomes (KEGG) analyses were performed using the ClusterProfiler R package. Finally, we performed a protein-protein interaction (PPI) network analysis using Cytoscape to identify key clusters.

### Development of an immune risk score (IRS)

Based on the time of inclusion of patients into the trial, TCGA-BLCA cohort was divided into training and validation sets with a ratio of 7:3. In the training set, we performed univariate Cox analysis in common DERs using the survival R package. Furthermore, the least absolute shrinkage and selector operation (LASSO) algorithm was applied to screen optimal candidate DERs (IRS RNA-expression profiles) with the best discriminative capability. We then developed an IRS based on the IRS RNA-expression profiles, weighted using the multivariate Cox regression coefficient as follows:

IRS=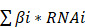


where β_i_ is the coefficient of the 'i'th IRS RNA-expression profile. Specifically, patients were classified into high and low IRS groups based on the median IRS. The Kaplan-Meier method was applied and the log-rank test was used to statistically compare the groups in order to estimate the prognostic significance of the IRS. The statistical performance of the IRS was assessed using the tROC R package. Additionally, the prognostic value of the IRS was validated in TCGA internal validation set. However, some RNAs included in the primary IRS algorithm could not be detected in external validation sets due to different RNA detection platforms. Therefore, we could not directly validate the performance of the primary IRS algorithm in external validation sets. However, we re-developed an IRS based on the common RNAs that were included in both, the IRS RNA-expression profiles and the external validation sets. Consequently, we determined the prognostic performance of the new IRS, which reflects the prognostic value of the IRS RNA expression profiles.

Since the IRS was developed based on immune DERs, we explored the role of IRS in predicting the clinical response of ICB in TCGA-BLCA cohort by analyzing the correlations between IRS and the immunological characteristics of an inflamed TME. Jiang et al. developed a signature of T cell dysfunction and exclusion (TIDE), which could accurately predict cancer immunotherapy response 55. Therefore, we compared the accuracy in predicting ICB response and survival probability between the IRS and TIDE algorithms in four immunotherapy cohorts, GSE78220, GSE91061, IMvigor210, and PMID29301960. The TIDE scores of samples in these cohorts were calculated and downloaded from http://tide.dfci.harvard.edu/.

### Statistical analysis

Correlations between variables were explored using Pearson or Spearman coefficients. Continuous variables fitting a normal distribution between binary groups were compared using a t-test. Otherwise, the Mann-Whitney U test was applied. Categorical variables were compared using the chi-squared test or Fisher's exact test. Survival curves for prognostic analyses of categorical variables were generated using the Kaplan-Meier method, while the log-rank test was applied to estimate statistical significance. The level of significance was set at *P* < 0.05, and all statistical tests were two-sided. All statistical data analyses were implemented using R software, version 3.6.3.

## Results

### Pan-cancer expression pattern, prognostic significance, and immunological correlation of Siglec15

After a comprehensive analysis of the expression data from TCGA, GTEx, and Oncomine databases, we found that Siglec15 was highly expressed in the majority of cancers such as BLCA and breast cancer (BRCA) compared with normal tissues (Figure S1A-D). Siglec15 was also expressed in various cancer cell lines, including bladder cancer cell lines, based on the screening of expression data from BioGPS and CCLE databases (Figure S1E-F). On the other hand, Siglec15 was expressed in very low amounts in normal tissues, except for macrophages (Figure S1G). In thirty paired samples consisting of bladder cancer and normal tissues, Siglec15 was found to be significantly and more highly expressed in cancer tissues than in normal tissues (Figure S1H). The pan-cancer overexpression pattern of Siglec15 prompted us to explore its prognostic value. Therefore, we performed a pan-cancer survival analysis concerning overall survival, progression-free survival, and cancer-specific survival using the Cox regression model, Kaplan-Meier analysis, and log-rank test. As anticipated, Siglec15 emerged as a prognostic biomarker in various cancers (Figure S2-S4), even though its prognostic value was variable in different cancers. However, these results need further evaluation, especially using multivariable analysis.

**Figure 2:**
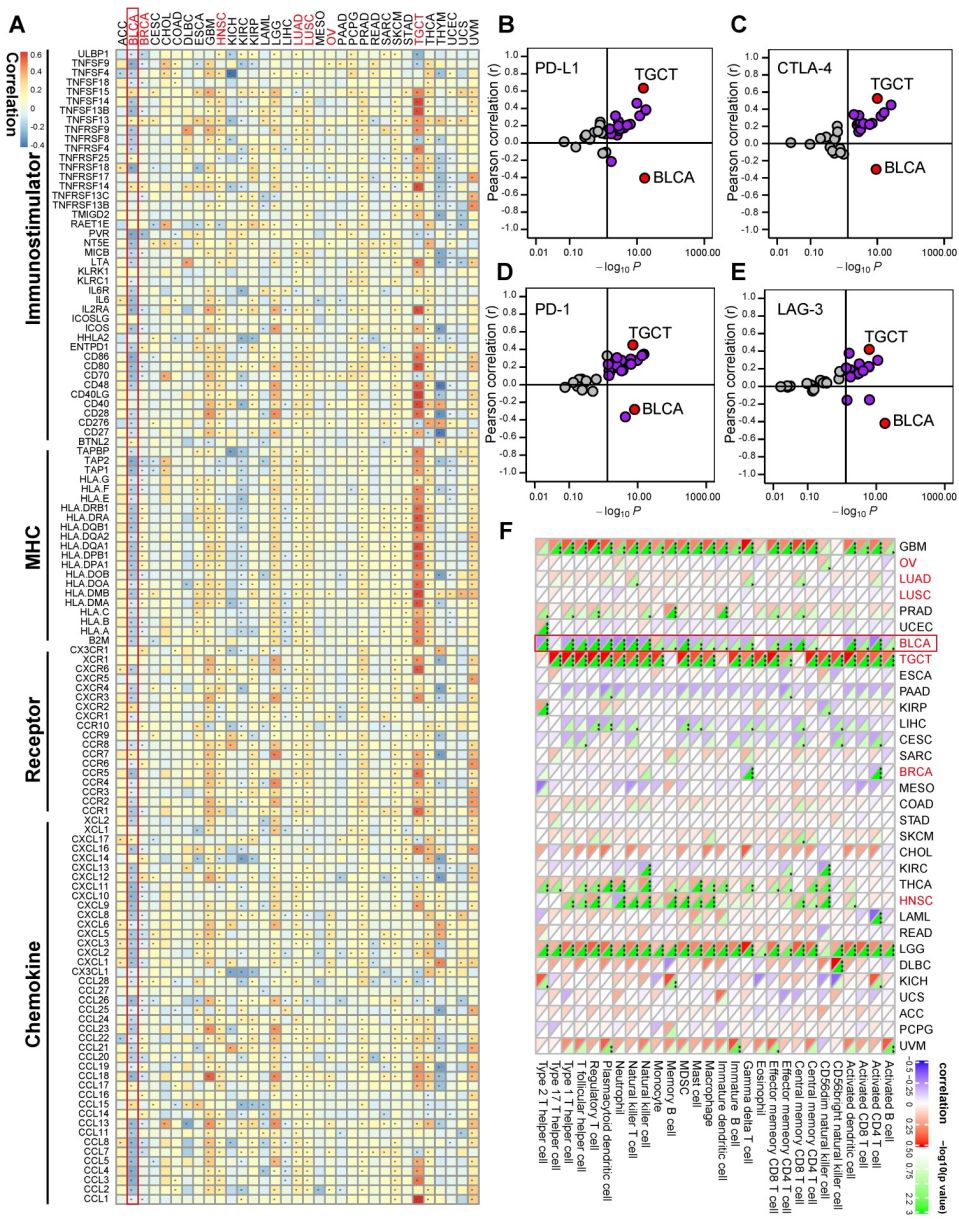
** The effect of Siglec15 on immunological status in pan-cancers.** (A) Correlation between Siglec15 and 122 immunomodulators (chemokines, receptors, MHC, and immunostimulators). (B-E) Correlation between Siglec15 and four immune checkpoints, PD-L1, CTLA-4, PD-1, and LAG-3. The dots represent cancer types. The Y-axis represents the Pearson correlation, while the X-axis represents -log_10_P. (F) Correlation between Siglec15 and 28 tumor-associated immune cells calculated with the ssGSEA algorithm. The color indicates the correlation coefficient. The asterisks indicate a statistically significant p-value calculated using spearman correlation analysis. (*P < 0.05; **P < 0.01; ***P < 0.001).

Pan-cancer analyses aimed at depicting the immunological role of Siglec15 are critical in determining the types of cancers that may benefit from anti-Siglec15 immunotherapy. Our findings revealed that Siglec15 was negatively correlated with a majority of immunomodulators in BLCA (Figure 2A). We also estimated the infiltration levels of TIICs in the TME using the ssGSEA algorithm. Likewise, Siglec15 was negatively correlated with the majority of TIICs in BLCA (Figure 2F). Furthermore, we demonstrated that the expression of Siglec15 was mutually exclusive of several immune checkpoints, including PD-L1, PD-1, CTLA-4, and LAG-3 in BLCA (Figure 2B-E, Table S2). Apart from BLCA, these negative immunological correlations of Siglec15 were not observed with other malignancies, such as NSCLC, ovarian cancer, melanoma, BRCA, and colorectal cancer. Nevertheless, we noted that Siglec15 was negatively correlated with TMB and MSI in several cancers, suggesting that Siglec15 may reflect cancer immunogenicity in these cancers (Figure S5).

In summary, the overexpression pattern of Siglec15 is TME specific, which demonstrates the potential of Siglec15 as a target for normalized cancer immunotherapy. The immunosuppressive effect of Siglec15 in TME is the most obvious in BLCA, which suggests that BLCA may be a suitable candidate cancer type for anti-Siglec15 immunotherapy.

### Mutational analyses of Siglec15 in BLCA

There were no mutations found in the Siglec15 gene. The CNV pattern of Siglec15 is shown in Figure S6A. Notably, copy number deletion and methylation of the Siglec15 reduced the expression of Siglec15 mRNA (Figure S6B-C). These results indicate that epigenetic modifications of the Siglec15 gene may be an alternative therapeutic method of intervention for anti-Siglec15 inhibitors. The top 30 mutational genes in the low- and high-Siglec15 groups, and an overview of the mutation profiles in BLCA are summarized in Figure S6D-F.

### Siglec15 shapes a non-inflamed TME in BLCA

**Figure 3:**
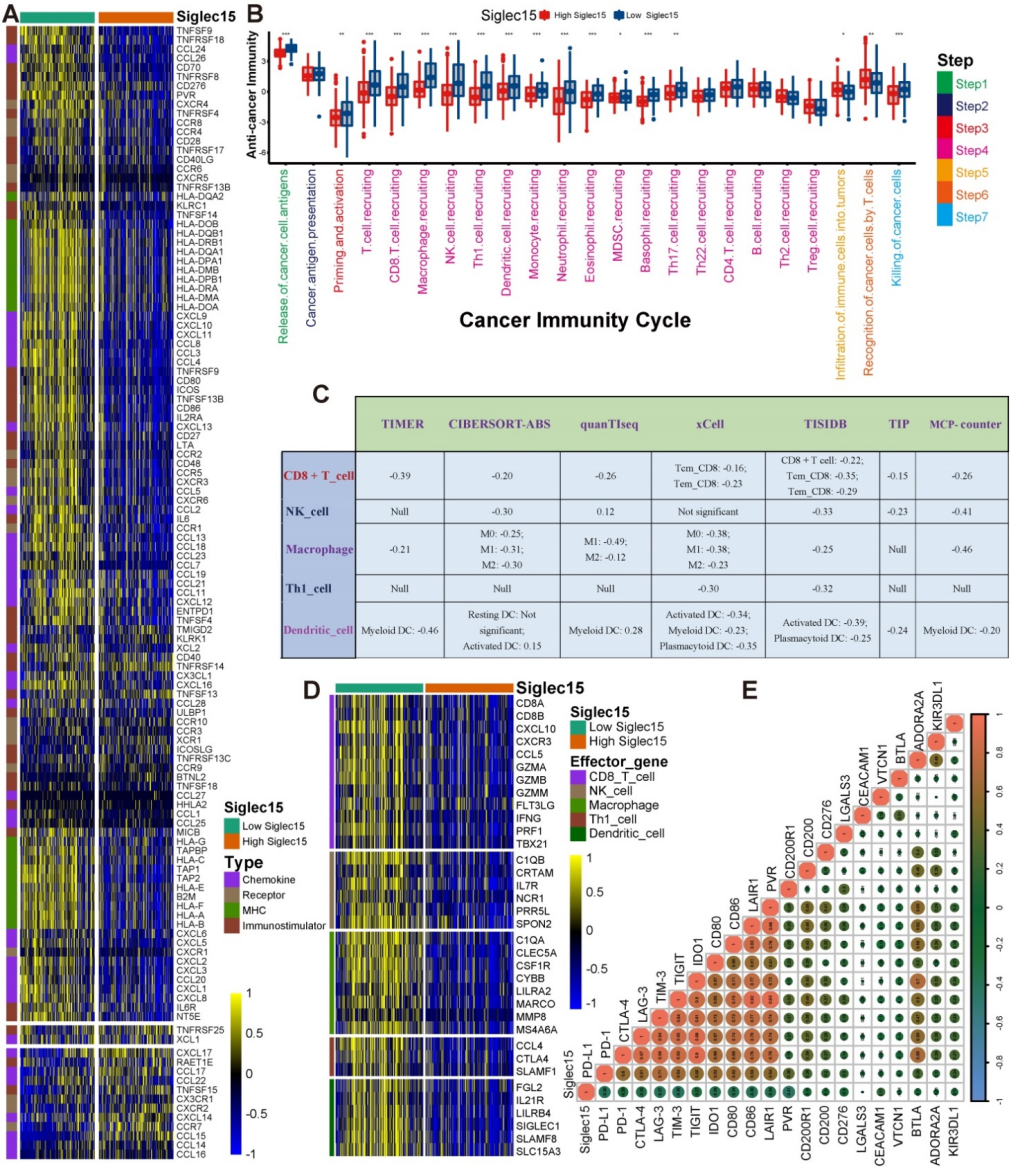
** Siglec15 shapes a non-inflamed TME in BLCA.** (A) Differences in the expression of 122 immunomodulators (chemokines, receptors, MHC, and immunostimulators) between high- and low-Siglec15 groups in BLCA. (B) Differences in the various steps of the cancer immunity cycle between high- and low-Siglec15 groups. (C) Correlation between Siglec15 and the infiltration levels of five types of TIICs (CD8+ T cells, NK cells, macrophages, Th1 cells, and dendritic cells), which were calculated using seven independent algorithms. (D) Differences in the effector genes of the above tumor-associated immune cells between high- and low-Siglec15 groups. (E) Correlation between Siglec15 and 20 inhibitory immune checkpoints. The color and the values indicate the Spearman correlation coefficient. The asterisks indicated a statistically significant p-value calculated using Mann-Whitney U test (*P < 0.05; **P < 0.01; ***P < 0.001).

Siglec15 was found to be negatively correlated with a large number of immunomodulators (Figure 3A, Table S2). A majority of MHC molecules were downregulated in the high-Siglec15 group, which indicated that the capacity of antigen presentation and processing was downregulated in the high-Siglec15 group. Three critical chemokines (CXCL9, CXCL10, and CCR3), which induce the recruitment of CD8+ T cells into the TME in BLCA, were downregulated in the high-Siglec15 group. Other chemokines, including CCL2, CCL3, CCL4, CCL5, CCL19, CCL20, CCL21, CXCL11, CXCL13, and paired receptors including CCR1, CCR2, CCR5, CCR6, and CXCR3, were negatively correlated with Siglec15. These chemokines and receptors promote the recruitment of effector TIICs such as CD8+ T cells, TH17 cells, and antigen-presenting cells. Given the complex and manifold functions of the chemokine system, the relationship between Siglec15 and individual chemokines was not sufficient to clarify the overall immunological effect of Siglec15 in TME.

The activities of the cancer immunity cycle are a direct comprehensive performance of the functions of the chemokine system and other immunomodulators 29, 30. In the high-Siglec15 group, activities of the majority of the steps in the cycle were found to be downregulated, including the release of cancer cell antigens (Step 1), priming and activation (Step 3), and trafficking of immune cells to tumors (Step 4) (CD8 T cell recruiting, Macrophage recruiting, Th1 cell recruiting, NK cell recruiting, DC recruiting, and TH17 recruiting) Siglec15(Figure 3B, Table S3). Subsequently, the reduced activities of these steps may reduce the infiltration levels of effector TIICs in the TME. Interestingly, the activity of recognition of cancer cells by T cells (Step 6) was downregulated in the low-Siglec15 group. This may be due to the high expression of PD-L1 in the low-Siglec15 group. The activity of Step 7 (killing of cancer cells) was downregulated in the high-Siglec15 group.

Next, we calculated the infiltration level of TIICs using seven independent algorithms (Figure S7-S13, Table S4). In line with the previous results, Siglec15 was negatively correlated with the infiltration level of CD8+ T cells, NK cells, Th1 cells, macrophages, and dendritic cells in different algorithms (Figure 3C). Similarly, Siglec15 was negatively correlated with the effector genes of these TIICs (Figure 3D, Figure S14A, Table S5). In addition, Siglec15 was negatively correlated with the marker genes of macrophages (Figure S14B-E). The expression of immune checkpoint inhibitors such as PD-L1/PD-1 was reported to be low in non-inflamed TME 7. Consistently, in this study, Siglec15 was found to be negatively correlated with a majority of immune checkpoint inhibitors including PD-L1, PD-1, CTLA-4, LAG-3, TIM-3, IDO1, and TIGIT (Figure 3E, Table S6).

**Figure 4:**
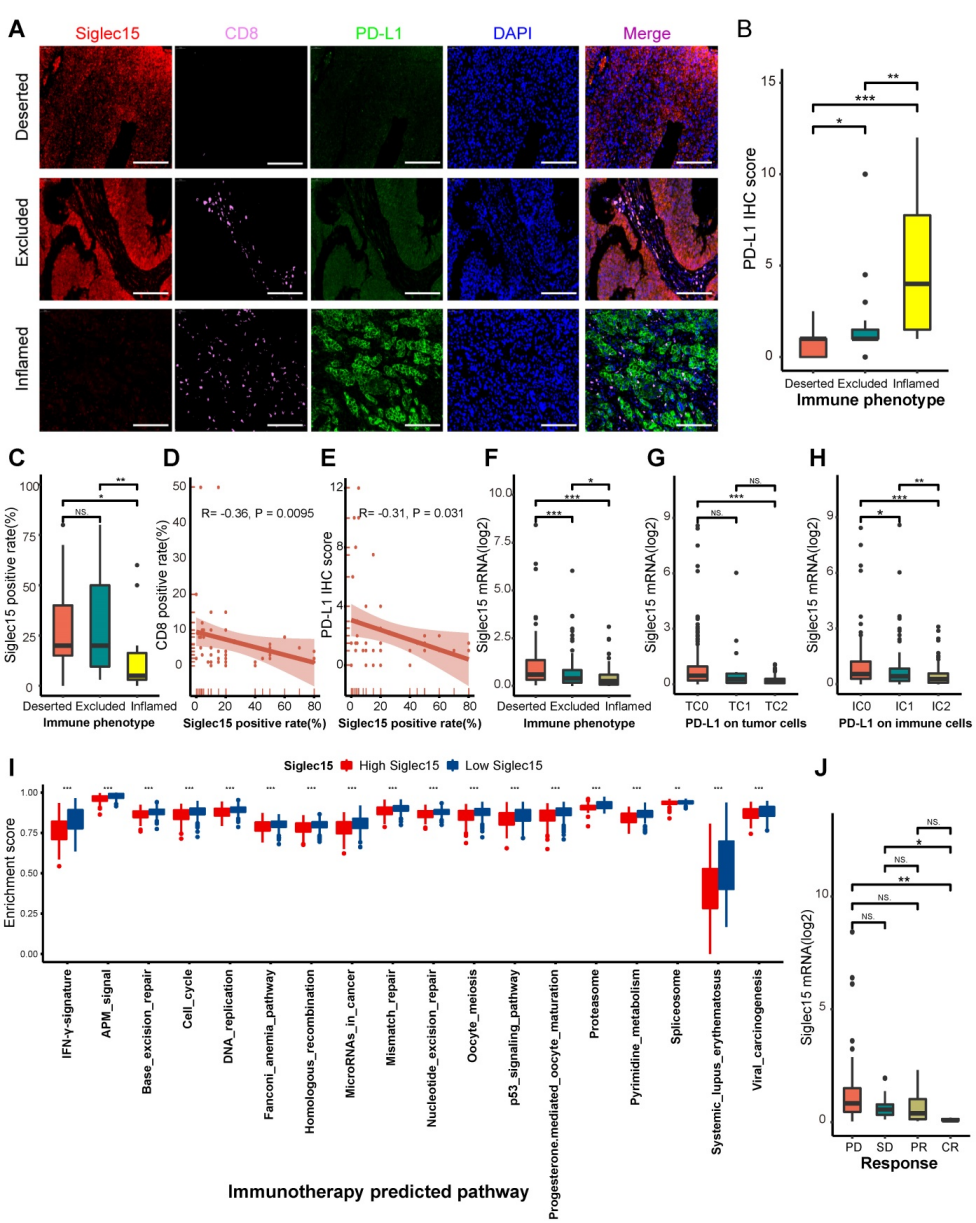
** Correlations between Siglec15, the immune phenotype, and the clinical response of immunotherapy in BLCA.** (A) Expression of Siglec15, CD8 and PD-L1 in the bladder cancer microarray (TMA) cohort were detected using immunofluorescence. Representative co-staining images of Siglec15, PD-L1, and CD8 in three immune phenotypes. Bladder cancer tissues were divided into three immune phenotypes, namely deserted, excluded, and inflamed phenotypes, based on the spatial distribution of CD8+ T cells. The scale bars correspond to 200 μm. (B-C) PD-L1 IHC score and the positive rate of Siglec15 (detected using immunofluorescence) in the three phenotypes of the TMA cohort. (D) Correlation between the Siglec15 positive rate and CD8 positive rate detected using immunofluorescence. (E) Correlation between the Siglec15 positive rate (detected using immunofluorescence) and PD-L1 IHC score. (F) Expression of Siglec15 in all three phenotypes in the IMvigor210 cohort. (G-H) Differences in the PD-L1 expression on tumor cells, and the PD-L1 expression on immune cells between high- and low-Siglec15 groups in the IMvigor210 cohort. (I) Differences in the enrichment scores of immunotherapy-predicted pathways between high- and low-Siglec15 groups in TCGA-BLCA cohort. (J) Correlation between Siglec15 and the clinical response of cancer immunotherapy in the IMvigor210 cohort. The asterisks indicate a significant statistical p-value calculated using the Mann-Whitney U test (*P < 0.05; **P < 0.01; ***P < 0.001). IHC: immunohistochemistry; PD: progressed disease; SD: stable disease; PR: partial response; CR: complete response.

These results were validated in a TMA cohort (Table S12). Samples in the TMA cohort were divided into three phenotypes, deserted, excluded, and inflamed, based on the spatial distribution of CD8+ T cells. Representative images are shown in Figure 4A and Figure S14F. The PD-L1 IHC score in the inflamed phenotype was the highest (Figure 4B).

Consistently, the inflamed phenotype had the highest CD8 positive rate (Figure S14G). These results indicated that the classification of these three immune phenotypes was suitable. We then analyzed the correlations between Siglec15, CD8, and PD-L1. The inflamed phenotype had the lowest Siglec15 expression among these three phenotypes (Figure 4C). Meanwhile, the Siglec15 expression was negatively correlated with CD8 expression (Figure 4D, Figure S14H). Also, the expression of PD-L1 was positively correlated with the expression of CD8 (Figure S14I). Finally, we found that the Siglec15 expression was negatively correlated with the PD-L1 expression (Figure 4E, Figure S14J).

Furthermore, we validated these results in three external cohorts with a larger sample size. In the IMvigor210 cohort, the expression of Siglec15 was higher in deserted phenotypes, TC0 (tumor cells with the lowest PD-L1 values), and IC0 (immune cells with the lowest PD-L1 values) groups (Figure 4F-H). Siglec15 was negatively correlated with a majority of immunomodulators, effector genes of TIICs, and immune checkpoints in GSE32894, GSE31684, and IMvigor210 cohorts (Figure S15A-C, Figure S16A-C, Figure S17A-C).

Collectively, Siglec15 strongly correlated with the development of a non-inflamed TME. However, despite the immunosuppressive properties of Siglec15 in BLCA, multivariate Cox analysis showed that it did not have any effect on the prognosis of BLCA (Table S1: survival analysis).

### Siglec15 predicts clinical response and hyperprogression of ICB in BLCA

**Figure 5:**
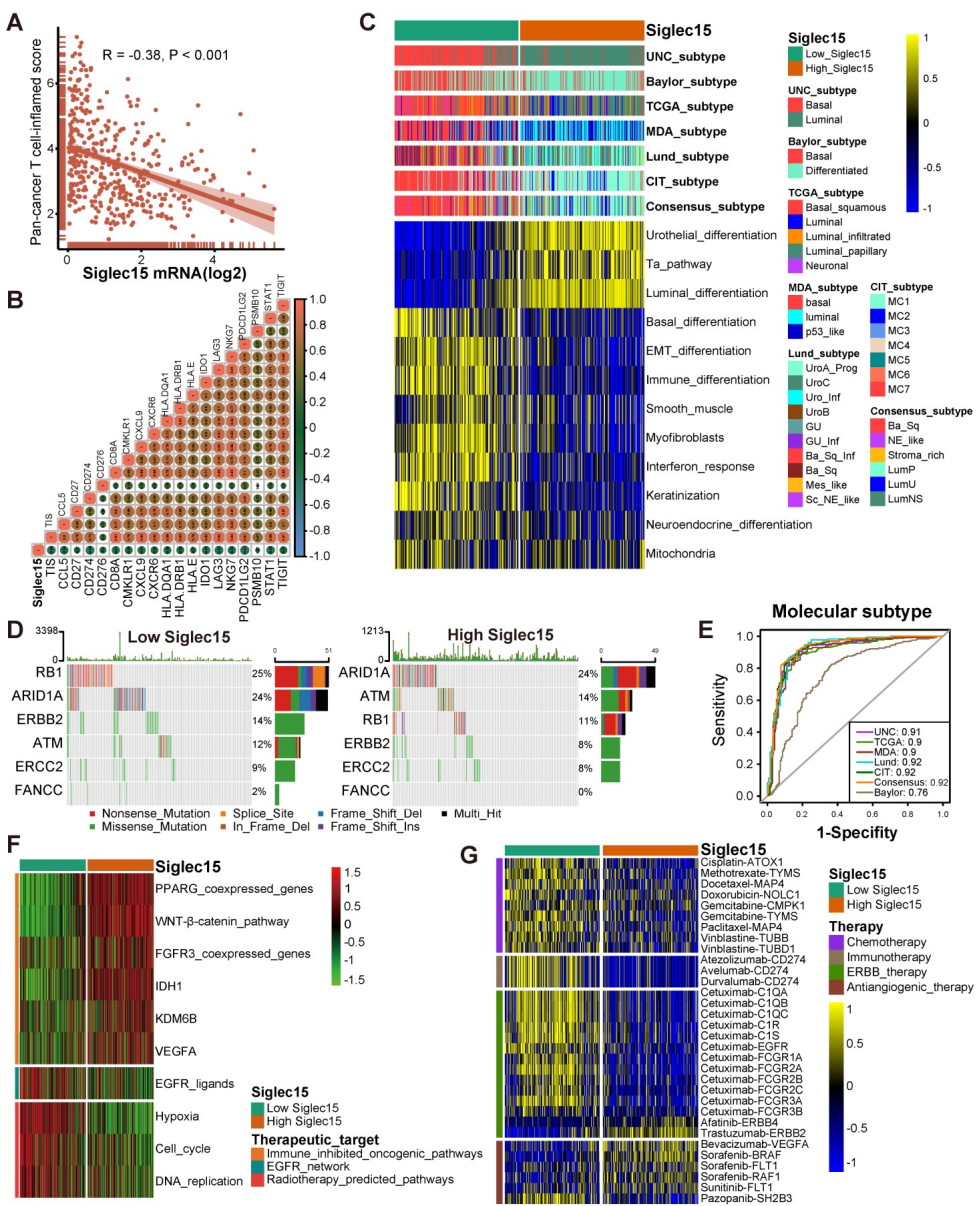
** Siglec15 predicts the molecular subtype and the therapeutic response to several therapies in BLCA.** (A-B) Correlations between Siglec15 and the pan-cancer T cell inflamed score and the individual genes included in the T cell inflamed signature. The T cell inflamed score is positively correlated with the clinical response to cancer immunotherapy. (C) Correlations between Siglec15 and molecular subtypes using seven different algorithms and bladder cancer signatures. (D-E) Mutational profiles of neoadjuvant chemotherapy-related genes in low- and high-Siglec15 groups. (E) Predictive accuracy of Siglec15 for molecular subtypes using seven different algorithms. The accuracy was equal to the area under the ROC curves. (F) Correlations between Siglec15 and the enrichment scores of several therapeutic signatures such as targeted therapy and radiotherapy. (G) Correlation between Siglec15 and the BLCA-related drug-target genes screened from the Drugbank database.

In theory, patients with higher Siglec15 expression should have a lower response to ICB because Siglec15 defines a non-inflamed TME. As expected, the expression of Siglec15 was significantly higher in patients with a progressive and stable disease compared to the patients showing a partial or complete response to therapy (Figure 4J). Siglec15 negatively correlated with the enrichment scores of most immunotherapy-positive gene signatures (Figure 4I, Table S13), which was validated in three external cohorts (Figure S15D, Figure S16D, Figure S17D). Furthermore, we analyzed the correlations between Siglec15 and various immune signatures (expression of immunomodulators and TIIC effector genes, immune checkpoints, and immunotherapy-related signatures) in subgroups with different ICB responses. These groups were defined as complete response, partial response, stable disease, and progressed disease groups. Results of the subgroup analyses indicated that high Siglec15 was negatively correlated with these immune signatures and predicted a lower response to immunotherapy in all subgroups (Figure S18-S21). In addition, Siglec15 was negatively correlated with the pan-cancer T cell inflamed score (R = -0.38, *P* < 0.001) (Figure 5A-B). Another concern was that the incidence of ICB-associated hyperprogression may be higher in the high-Siglec15 group. The copy number amplification rates and mRNA expression of genes positively correlated with hyperprogression, including CCND1, FGF3, FGF4, FGF19, MDM2, MDM4, and DNMT3A, were significantly higher in the high-Siglec15 group (Figure S22A-B). In contrast, the mRNA expression of genes negatively correlated with hyperprogression, including CDKN2A and CDKN2B, was significantly lower in the high-Siglec15 group (Figure S22A-B).

In summary, ICB should not be implemented in BLCA patients with high-Siglec15 expression as they are not responsive to ICB and instead exhibit a higher incidence of hyperprogression.

### Siglec15 predicts molecular subtypes and therapeutic opportunities

Findings from the PURE-01 study elucidated that basal-type BLCA showed the highest immune cell infiltration and pathological response rates to pembrolizumab 56. In addition, the consensus subtype revealed a similar conclusion that basal-type tumors were more likely to respond to ICB 19. BLCA with lower Siglec15 expression was more likely to be the basal subtype among the seven molecular subtyping systems (Figure 5C). This re-validated the conclusion that Siglec15 was negatively correlated with the response to ICB. In addition, the enrichment scores for luminal differentiation, Ta pathway, and urothelial differentiation were greater in the high-Siglec15 group. On the other hand, the enrichment scores for basal differentiation, EMT differentiation, immune infiltration, and interferon response were lower in the high-Siglec15 group (Figure 5C). These outcomes were validated using three external cohorts (Figure S15E, Figure S16E, Figure S17E). Moreover, except for the Baylor molecular subtyping system, the area under the ROC curves (AUC) of Siglec15 in other systems was ≥ 0.90 (Figure 5E). We observed similar findings, using four cohorts for validation (Figure S22C-F).

A molecular subtype can also predict the clinical response to neoadjuvant chemotherapy, radiotherapy, and several targeted therapies 19, 57. Basal subtype tumors were more likely to respond to neoadjuvant chemotherapy. The mutation rates of RB1, ERBB2, and FANCC were significantly higher in the low-Siglec15 group (basal subtype**)** (Figure 5D). In addition, the enrichment scores for radiotherapy-predicted pathways and EGFR ligands were higher in the low-Siglec15 group (Figure 5F, Table S13). Furthermore, results from the Drugbank database indicated a significantly higher response to chemotherapy, immunotherapy, and ERBB therapy in the low-Siglec15 group (Figure 5G). This shows that ICB, neoadjuvant or adjuvant chemotherapy, and ERBB therapy can be used, either alone or in combination, for the treatment of BLCA with low Siglec15 expression.

BLCA with higher Siglec15 expression was more likely to be the luminal subtype (Figure 5C). ICB, chemotherapy, and radiotherapy were all unsuitable for BLCA with high Siglec15 expression. The enrichment scores for several immunosuppressive oncogenic pathways were significantly higher in the high-Siglec15 group (Figure 5F, Table S13). These oncogenic pathways were related to the non-inflamed TME in BLCA. Consequently, inhibiting these pathways promoted the formation of an inflamed TME, thereby reactivating cancer immunity 13, 58. In line with this observation, drugs targeting the PPARG and FGFR pathways have achieved promising results in BLCA. Likewise, erdafitinib (an FGFR inhibitor) achieved an excellent response in metastatic BLCA with a prior ICB therapy 59. Theoretically, Siglec15 shares a similar immunosuppressive function with these oncogenic pathways. Therefore, targeted therapy blocking these pathways can be used in combination with anti-Siglec15 therapy for the treatment of BLCA with high Siglec15 expression. These results were validated in three external cohorts (Figure S15F, Figure S16F, Figure S17F). We found that anti-angiogenic therapy may be suitable for BLCA with high Siglec15 expression (Figure 5G, Table S10).

### Siglec15 predicts immune phenotypes and molecular subtypes in Xiangya cohort

**Figure 6:**
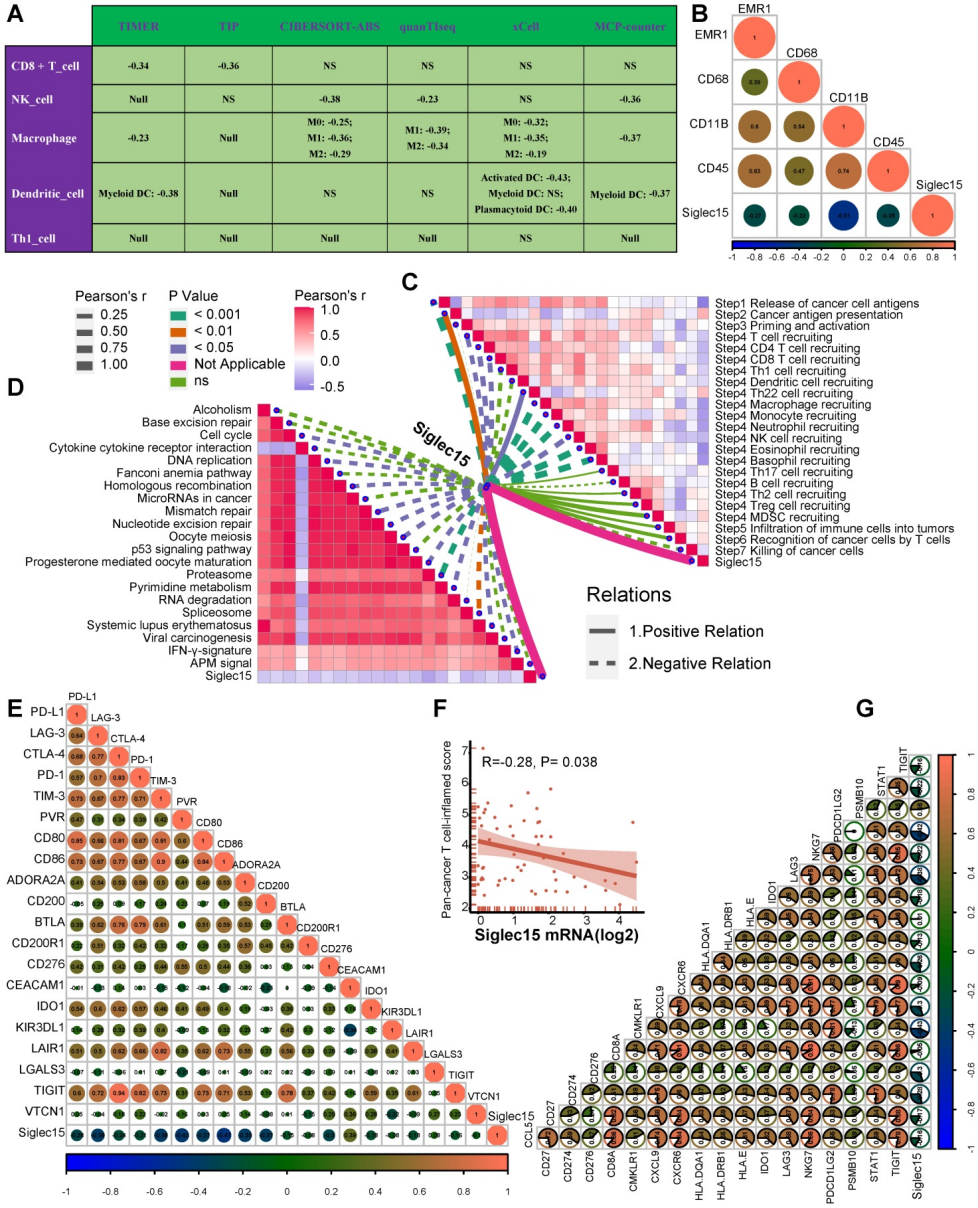
** Roles of Siglec15 in predicting immune phenotypes in the Xiangya cohort.** (A) Correlations between Siglec15 and the infiltration levels of five tumor-associated immune cells (CD8+ T cells, NK cells, macrophages, Th1 cells, and dendritic cells). (B) Correlations between Siglec15 and four critical marker genes of macrophages. (C) Correlations between Siglec15 and the steps of the cancer immunity cycle. (D) Correlations between Siglec15 and the enrichment scores of immunotherapy-predicted pathways. (E) Correlations between Siglec15 and immune checkpoints. (F-G) Correlations between Siglec15 and the T cell inflamed score.

In our cohort, Siglec15 negatively correlated with a majority of immunomodulators, such as CXCL9, CXCL10, CCL2, CCL3, CCL4, CCL19, and others (Table S14). Siglec15 was also found to be negatively correlated with CD8+ T cells, NK cells, dendritic cells, and macrophages in multiple algorithms (Figure 6A). Siglec15 also negatively correlated with four critical marker genes of macrophages (Figure 6B). As expected, Siglec15 negatively correlated with the critical steps of the cancer-immunity cycle, including the release of cancer cell antigens (Step 1), and trafficking of immune cells to tumors (Step 4) (CD8 T cell recruiting, Macrophage recruiting, Th1 cell recruiting, NK cell recruiting, DC recruiting, and TH17 recruiting) (Figure 6C). We also analyzed the correlations between Siglec15 and the predicted ICB response signatures. Siglec15 negatively correlated with the enrichment scores for all immunotherapy-related positive signatures (Figure 6D). Furthermore, Siglec15 was also negatively correlated with a majority of immune checkpoints (such as PD-L1, LAG-3, and CTLA-4) and the T cell-inflamed score (Figure 6E-G).

In summary, Siglec15 can accurately distinguish basal and luminal subtypes in seven different molecular subtype algorithms (Figure S23A-B). BLCA with higher Siglec15 expression was more likely to be the luminal subtype. The accuracy of Siglec15 in predicting molecular subtypes ranged from 0.81 to 0.91 in seven algorithms. Furthermore, the roles of Siglec15 in predicting therapeutic responsiveness to neoadjuvant or adjuvant chemotherapy, radiotherapy, and targeted therapy were successfully validated (Figure S23C).

### Identifying immune-related DERs

In this study, we identified 1500 common DERs (Figure S24, Table S15). Interestingly, there was no commonality between DERs upregulated in the high-Siglec15 group and the high immune/stromal score groups. Similarly, there was no intersection between DERs downregulated in the high-Siglec15 group and the high immune/stromal score groups (Figure S24H-K). This revealed that Siglec15 was negatively correlated with the immune and stromal scores in the TME. As expected, several basal subtype-specific genes, including KRT6A, KRT6B, KRT6C, KRT5, and KRT14, were upregulated in the low-Siglec15 group **(**Figure S24A). Also, several luminal subtype-specific genes, including UPK1A, UPK2, UPK3A, and KRT20 were upregulated in the high-Siglec15 group **(**Figure S24A). This implied that Siglec15 expression levels could predict the molecular subtype of BLCA. Results based on GO and KEGG analyses suggested that these DERs were enriched in the immune-related functional processes (Figure S25, Table S16). Findings from PPI analyses identified 24 clusters. Among these 24 clusters, the top three clusters and corresponding hub genes were also associated with the immune-related processes (Table S17).

### Development and validation of an IRS

**Figure 7:**
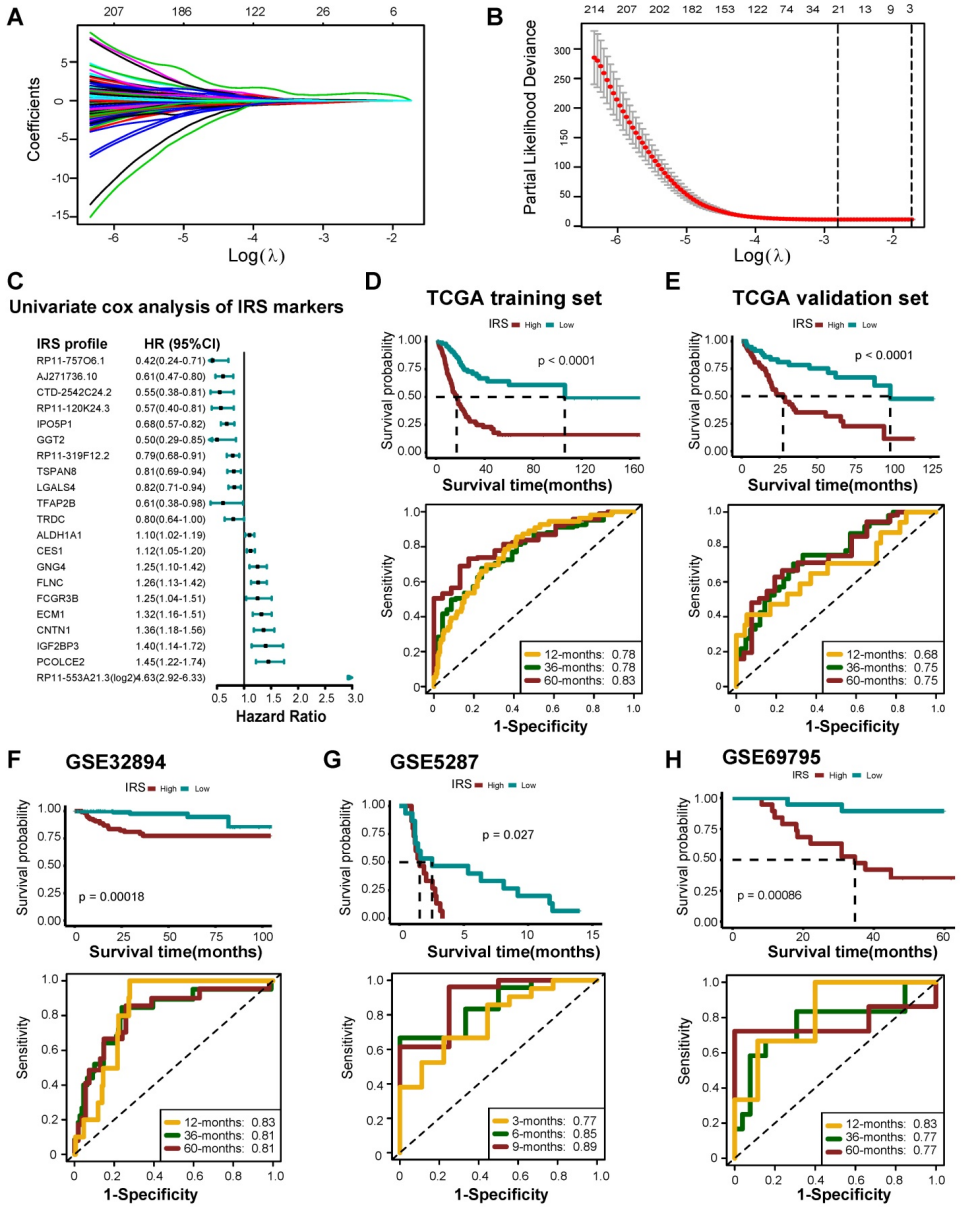
** Developing IRS RNA-expression profiles using LASSO Cox regression.** (A) LASSO coefficient profiles of 524 prognostic RNAs in TCGA training cohort. The coefficient profile plot was developed against the log (Lambda) sequence. (B) Cross-validation for turning parameter selection via minimum criteria in the LASSO regression model. Two dotted vertical lines were plotted at the optimal values using the minimum criteria. Optimal RNAs with the best discriminative capability (21 in number) were selected for developing the IRS. (C) Forest plot of the IRS RNA-expression profiles in univariate cox analysis. (D) Development of IRS in TCGA training set and the predictive accuracy of IRS for survival. (E) Validation of the IRS in TCGA validation set. (F-H) Validation of the IRS RNA-expression profiles in three external independent sets: GSE32894, GSE5287, and GSE69795.

Overall, 524 DERs were found to affect prognosis based on univariate Cox analysis (Table S18). We then identified 21 best candidate DERs (IRS RNA-expression profiles) with minimal lambda (0.001765) (Figure 7A-C) using the LASSO algorithm. IRS was developed according to the IRS RNA-expression profiles using multivariate Cox regression analysis. In TCGA training set, 275 patients were classified in the high IRS group (n=137) and low IRS group (n=138) using the median value of IRS as the risk cut-off. As shown in Figure 7D, patients with low IRS had significantly longer overall survival time than those with high IRS. The AUC of IRS was 0.78, 0.78, and 0.83 at 12, 36, and 60 months, respectively. The predictive accuracy of the IRS was well validated in TCGA internal validation set (Figure 7E). We further validated the prognostic value of the IRS RNA-expression profiles in several external BLCA cohorts. Our findings demonstrate that the IRS RNA-expression profiles were a valuable prognostic panel in all BLCA cohorts (Figure 7F-H, Figure S26A-D, Figure S27, Figure S28). In addition, the prognostic value of the IRS RNA-expression profiles can be validated in GSE135222 (NSCLC) (Figure S26E).

Apart from the prognostic value, IRS can predict the clinical response to ICB. Remarkably, the IRS was negatively related to Siglec15, but positively related to the pan-cancer T cell inflamed score (Figure S29A-B). The expression of several immune checkpoints, such as PD-L1, CTLA-4, and LAG-3, was significantly higher in the high IRS group (Figure S29C). Meanwhile, the IRS was positively related to several immunomodulators, effector TIICs, and cancer immunity cycle activities (Figure S29D-F). Lastly, the enrichment scores of most immunotherapy positively related signatures were significantly higher in the high IRS group (Figure S29G).

Finally, we compared the accuracy in predicting ICB response between the IRS and TIDE algorithms, and we also evaluated the prognostic value of these two algorithms. In GSE78220 (melanoma), TIDE behaved better in predicting the ICB response than IRS (C-index: 0.76 Vs. 0.69) (Figure S30A) Meanwhile, high TIDE or IRS predicted poor prognosis (Figure S30B-C). However, IRS performed better in predicting prognosis than TIDE (Figure S30D-E). In GSE91061 (melanoma), there was no difference in the predictive accuracy in predicting ICB response between the two algorithms (Figure S31A). However, TIDE was not related to prognosis (Figure S31B). Notably, IRS performed better in predicting prognosis than TIDE (Figure S31C-E). In PMID29301960 (KIRC), IRS behaved better in predicting ICB response than TIDE (C-index: 0.75 vs. 0.56) (Figure S32A). Similarly, IRS performed better in predicting prognosis than TIDE (Figure S32B-E). However, the predictive accuracy of TIDE or IRS for ICB response or prognosis was low in the IMvigor210 cohort (Figure S33). In general, TIDE and IRS were comparable in predicting ICB response. As for the prognostic value, IRS may behave better than TIDE.

## Discussion

In this study, we demonstrated that bladder cancer (BLCA) is a suitable candidate for anti-Siglec15 immunotherapy. We further confirmed that Siglec15 shaped a non-inflamed TME based on the evidence that Siglec15 negatively correlated with the immunological status of TME in BLCA. In addition, we elucidated that Siglec15 could accurately predict the clinical response of ICB as well as the molecular subtypes, and the response to several therapies. Finally, we developed an IRS to predict prognosis and clinical response to ICB.

Furthermore, whether a molecule can be a target for normalization cancer immunotherapy depends on two necessary characteristics: TME-specific over-expression and immunosuppressive function 60. Siglec15 was overexpressed TME-specifically in various cancers, which implies fewer side effects of anti-Siglec15 treatment. This has been validated in preclinical research using mouse models 20. Findings from preclinical research and phase I clinical trials of Siglec15 inhibitors indicated that Siglec15 may be a broad-spectrum therapeutic target 20. However, in this case, pan-cancer analyses revealed that Siglec15 exerted no immunosuppressive effect in the majority of cancers, including lung adenocarcinoma, lung squamous cell carcinoma, BRCA, head and neck squamous cell carcinoma, and OV, which were enrolled in a phase II clinical trial of Siglec15 inhibitor. Meanwhile, Siglec15 was positively related to PD-L1, PD-1, CTLA-4, and LAG-3 in most cancers. These results provide an insight into why the further progress of the phase II clinical trial in NSCLC and OV was hindered. Finally, BLCA was identified as the ideal cancer for anti-Siglec15 immunotherapy.

The cancer immunity cycle represents the immune response of our body to cancer. The activities of the cancer-immunity cycle comprehensively reflect the final effect of the complex immunomodulatory interactions in TME. Here, we noted that Siglec15 was negatively correlated with the activities of several steps of the cancer immunity cycle. For example, the activity of T cell recruitment was significantly downregulated in the high-Siglec15 group. Consequently, the infiltration levels of several effector TIICs, such as CD8+ T cells, NK cells, macrophages, TH1 cells, and dendritic cells, were also significantly decreased. These results can be validated in different algorithms or external validation cohorts. Therefore, Siglec15 defines a non-inflamed TME. Another critical characteristic of an inflamed TME is the upregulation of inhibitory immune checkpoints such as PD-L1/PD-1, which are driven by pre-infiltrating TIICs 61. These immune checkpoints suppress pre-existing cancer immunity to avoid excessive immune response, but also lead to immune evasion. The ICB targeting these immune checkpoints has achieved promising survival benefits in advanced BLCA 3, 4. In our study, the expression of inhibitory immune checkpoints was significantly downregulated in the high Siglec15 group, which might be attributed to the downregulation of pre-existing TIICs. This suggested that BLCA with high Siglec15 expression was not sensitive to ICB. Further, we consistently found that both the clinical response rates to ICB, and CD8+ T cell infiltration and PD-L1 expression were significantly downregulated in the high-Siglec15 group in the IMvigor210 cohort. Meanwhile, Siglec15 was negatively related to the T cell inflamed score and the enrichment scores of immunotherapy-predicted pathways. Another concern is that ICB-associated hyperprogression was more likely to occur in the high-Siglec15 group. In this respect, the exploration of alternative treatment options is urgently needed for BLCA, particularly with high Siglec15 expression.

Wang et al. demonstrated that Siglec15 promotes immune evasion by inhibiting the proliferation of CD8+ T cells 20. However, we found that Siglec15 may exert an immunosuppressive function by comprehensively downregulating the expression of critical immunomodulators such as CXCL9, CXCL10, and CXCR3, and subsequently downregulating the activities of the cancer-immunity cycle. Subsequently, the recruitment of effector TIICs decreased, thereby promoting the development of a non-inflamed TME. To a certain extent, the immunological role of Siglec15 was similar to those of several reported immunosuppressive oncogenic pathways, including β-catenin, PPAR-γ, and FGFR3 pathways 13-15. These oncogenic pathways have been reported to impair the infiltration of TIICs by reducing the expression of immunomodulators; hence, shaping a non-inflamed TME 13. Siglec15 was significantly positively correlated with the enrichment scores of these oncogenic pathways. However, it seems that Siglec15 may exert a wider range of effects on the anticancer immune response compared to these pathways. These outcomes not only provide clues for subsequent research on the mechanism of Siglec15 in immune regulation, but also lay the foundation for developing new treatment options. Reversing these oncogenic mechanisms may make the tumor immunologically “hot” and trigger the anticancer immune response in the TME 12, 59, 62. Likewise, a prior treatment option for BLCA with high Siglec15 expression was to transform a non-inflamed TME into an inflamed TME and consequently trigger an anti-cancer immune response. The expression of inhibitory immune checkpoints will increase due to negative feedback regulation. Therefore, subsequent ICB therapy may reactivate the suppressed anticancer immunity and enhance the efficacy of anti-Siglec15 therapy, which mainly triggers anticancer immunity. A combination of different ICB drugs was noted to be more effective than monotherapy 63, 64. However, therapeutic targets, such as PD-L1, PD-1, and CTLA-4, of the current ICB were positively correlated with each other in BLCA. Therefore, a combination of these drugs appears to have overlapping clinical applications. In contrast, Siglec15 was significantly negatively correlated with these ICB therapeutic targets, which suggests that anti-Siglec15 therapy in combination with ICB exhibited complementary effects.

Wang et al. found that the cytokine, macrophage colony-stimulating factor, acts as a positive regulator in myeloid cells and promotes the expression of Siglec15 20. Interestingly, we found that CSF1R and CSF1 were downregulated in samples with high Siglec15 expression. There could be several reasons for this discrepancy. First, the study performed by Wang et al. was conducted in vivo and in vitro rather than in the human tumor microenvironment. There are many biological differences between the in vivo/in vitro systems and the human tumor microenvironment. For example, the number and types of tumor-infiltrating immune cells, tumor cells, immunomodulators, and the extracellular matrix composition may have an effect on the level of macrophages and the expression of Siglec15. However, it is difficult to consider all of these factors at the same time in an in vivo/in vitro study. Second, the results of our study suggest that Siglec15 is mainly expressed in bladder cancer cells rather than macrophages. Several findings support this conclusion. Siglec15 was negatively related to the infiltration levels of tumor-associated macrophages in multiple immune cell estimation algorithms, such as TIMER and CIBERSORT. Furthermore, we found that Siglec15 was negatively related to the effector genes of macrophages, including C1QA, CLEC5A, CYBB, LILRA2, MARCO, MMP8, and MS4A6A. We further revealed that Siglec15 was negatively related to four critical marker genes of macrophages, including EMR1 (F4/80), CD68, PTPRC (CD45), and ITGAM (CD11b). In addition, the activities of macrophage recruitment and monocyte recruitment were significantly lower in the high-Siglec15 group. Therefore, we hypothesized that Siglec15 expressed from bladder cancer cells may decrease the infiltration level of macrophages and monocytes by downregulating the recruiting ability of macrophages. As a result, Siglec15 was negatively related to CSF1R and CSF1 in our study.

A molecular subtype can explain the heterogeneity of BLCA at the molecular level; thus, it can be used to predict the prognosis and the response to several treatment options, including neoadjuvant chemotherapy and ICB 19, 49-54. Recent studies have focused on developing more convenient and economical molecular subtyping methods for clinical applications. For instance, Eckstein et al. created an ImmuneTyper based on the expression of three cytotoxic T cell-related genes (CD3Z, CD8A, and CXCL9), detected using reverse transcription-quantitative polymerase chain reaction. This ImmuneTyper can assess immunological status, stratify the molecular subtype, and predict the prognosis of BLCA 65. Alternatively, Woerl et al. developed a pathological deep learning algorithm based on whole histological slide images, which can precisely predict individual molecular subtypes in BLCA 66. Here, we explored an individual's molecular subtype using seven different algorithms. Except for with the Baylor molecular subtype algorithm, the AUCs of Siglec15 in predicting the molecular subtype were ≥ 0.90. The Baylor molecular subtype algorithm was developed based on an 18-gene signature selected from urothelial cellular differentiation. Therefore, this molecular subtype algorithm is a simple classification, which also explains why the accuracy of Siglec15 in this algorithm was slightly lower. Moreover, the predictive accuracy of Siglec15 for molecular subtypes can also be validated from the perspective of therapeutic responses. Basal subtype tumors (low-Siglec15 group) were more responsive to ICB and neoadjuvant chemotherapy compared to luminal subtype tumors (high-Siglec15 group). For BLCA in the high-Siglec15 group, targeted therapy such as blocking Siglec15, β-catenin, PPAR-γ, and FGFR3 pathways and anti-angiogenic therapy may be valuable alternative options.

Finally, we developed and validated an IRS to predict prognosis and response to ICB based on the IRS RNA-expression profiles. Of note, the IRS RNA-expression profiles were generalized and robust in external validation cohorts. That said, there were also a few limitations to this study. First, the sample size of our TMA cohort was small. Second, although our results were validated in many external validation cohorts, the batch effects from different cohorts should be considered to confirm these intriguing findings. Third, we did not determine the optimal cut-off value of Siglec15. Here, the median Siglec15 mRNA expression was considered as the cut-off value. Finally, further experiments are needed to determine the expression profiles of Siglec15 in tumor cells and TIICs.

## Conclusions

This study demonstrated that bladder cancer may be a suitable candidate for anti-Siglec15 immunotherapy. We show that Siglec15 shapes a non-inflamed TME in BLCA and can also predict the clinical response to ICB and the BLCA molecular subtype.

## Supplementary Material

Supplementary figure and table legends.

Supplementary figures 1-19.

Supplementary figures 20-33.

Supplementary tables.
