# Supplementary material for: Siglec15 shapes a non-inflamed tumor microenvironment and predicts the molecular subtype in bladder cancer
Source: Theranostics. 2021 Jan 1;11(7):3089–108. doi: 10.7150/thno.53649 (PMC7847675; doi:10.7150/thno.53649)
Supplement: Supplementary file 1 — Supplementary figure and table legends. [file thnov11p3089s1.pdf]

# Supplementary files

**Figure S1. Expression pattern and immunological correlation of Siglec15 in pan-cancers.** (A-C) The expression pattern of Siglec15 of pan-cancers in TCGA, TCGA combined with GTEx, and Oncomine. The asterisks indicated a significant statistical p value calculated with Mann-Whitney U test.(*P < 0.05; **P < 0.01; ***P < 0.001). (D) The expression of Siglec15 in BLCA cohort in Oncomine. (E-F) The expression of Siglec15 in cancer cell lines in BioGPS and CCLE. (G) The expression of Siglec15 in normal tissues. (H) Quantitative RT-PCR (qPCR) estimation on Siglec-15 mRNA levels in 30 paired bladder cancer and normal tissues.

**Figure S2. Prognostic analysis of Siglec15 for overall survival in pan-cancers.** (A) The prognostic analyses of Siglec15 in pan-cancers using a univariate Cox regression model. Hazard ratio >1 indicated a risk factor and hazard ratio <1 represented a protective factor. (B-G) The prognostic analyses of Siglec15 in pan-cancers using Kaplan-Meier method and log-rank test. Only cancers in which Siglec15 was a significant prognostic biomarker were shown.

**Figure S3. Prognostic analysis of Siglec15 for disease specific survival in pan-cancers.** (A) The prognostic analyses of Siglec15 in pan-cancers using a univariate Cox regression model. Hazard ratio >1 indicated a risk factor and hazard ratio <1 represented a protective factor. (B-G) The prognostic analyses of Siglec15 in pan-cancers using Kaplan-Meier method and log-rank test. Only cancers in which Siglec15 was a significant prognostic biomarker were shown.

**Figure S4. Prognostic analysis of Siglec15 for progression free survival in pan-cancers.** (A) The prognostic analyses of Siglec15 in pan-cancers using a univariate Cox regression model. Hazard ratio >1 indicated a risk factor and hazard ratio <1 represented a protective factor. (B-G) The prognostic analyses of Siglec15 in pan-cancers using Kaplan-Meier method and log-rank test. Only cancers in which Siglec15 was a significant prognostic biomarker were shown.

**Figure S5. Correlations between Siglec15 and TMB, MSI in pan-cancers.** (A) Correlation between Siglec15 and TMB in pan-cancers. (B) Correlation between Siglec15 and MSI in pan-cancers. The asterisks indicated a significant statistical p value calculated with spearman correlation analysis.(*P < 0.05; **P < 0.01; ***P < 0.001).

**Figure S6. Multi-omics analysis of Siglec15 in BLCA.** (A) CNV pattern of Siglec15 in BLCA. (B) Effect of Siglec15 CNV pattern on the expression of Siglec15 mRNA. The asterisks indicated a significant statistical p value calculated with Mann-Whitney U test (*P < 0.05; **P < 0.01; ***P < 0.001). (C) Effect of Siglec15 methylation on the expression of Siglec15 mRNA. (D-E) The top 30 mutational genes in low and high Siglec15 group respectively. The upper barplot showed the TMB, the number on

the right showed the mutation frequency. The right barplot represented the proportion of variant types. (F) Overview of mutation profiles in BLCA.

**Figure S7. Correlations between Siglec15 and the tumor associated immune cells calculated with TIMER algorithm.** The p value was calculated with spearman correlation analysis.

**Figure S8. Correlations between Siglec15 and the tumor associated immune cells calculated with CIBERSORT-ABS algorithm.** The p value was calculated with spearman correlation analysis.

**Figure S9. Correlations between Siglec15 and the tumor associated immune cells calculated with quanTIseq algorithm.** The p value was calculated with spearman correlation analysis.

**Figure S10. Correlations between Siglec15 and the tumor associated immune cells calculated with xCell algorithm.** The p value was calculated with spearman correlation analysis.

**Figure S11. Correlations between Siglec15 and the tumor associated immune cells calculated with TISIDB algorithm.** The p value was calculated with spearman correlation analysis.

**Figure S12. Correlations between Siglec15 and the tumor associated immune cells calculated with TIP algorithm.** The p value was calculated with spearman correlation analysis.

**Figure S13. Correlations between Siglec15 and the tumor associated immune cells calculated with MCP-counter algorithm.** The p value was calculated with spearman correlation analysis.

**Figure S14. Correlations between Siglec15, tumor associated immune cells and immune phenotypes.** (A) Correlations between Siglec15 and the effector genes of five tumor associated immune cells . (B-E) Correlations between Siglec15 and four critical marker genes of macrophages. (F) Expression of Siglec15, PD-L1, and CD8 in the bladder cancer microarray (TMA) cohort were detected using immunohistochemistry. Representative images of CD8, PD-L1, and Siglec15 in three immune phenotypes were displayed. The scale bars correspond to 200 μm. (G) CD8 positive rates in the three immune phenotypes in BLCA TMA cohort detected by immunofluorescence. (H) Correlation between Siglec15 positive rates and CD8 positive rates detected using immunohistochemistry. (I) Correlation between PD-L1 positive rates and CD8 positive rates detected using immunofluorescence. (I) Correlation between PD-L1 positive rates and Siglec15 positive rates detected using immunofluorescence.

**Figure S15. Correlations between Siglec15 and the immunological status, the enrichment scores of therapeutic signatures, and the molecular subtype in GSE32894 cohort.** (A-C) Correlation between Siglec15 and immunomodulators, effector genes of tumor associated immune cells, and inhibitory immune checkpoints in BLCA. (D) Correlation between Siglec15 and enrichment scores of immunotherapy predicted signatures. The asterisks indicated a significant statistical p value calculated with Mann-Whitney U test (*P < 0.05; **P < 0.01; ***P < 0.001). (E) Correlation between Siglec15 and molecular subtype and bladder cancer signatures. (F) Correlation between Siglec15 and enrichment scores of therapeutic signatures, including radiotherapy, targeted therapy.

**Figure S16. Correlations between Siglec15 and the immunological status, the enrichment scores of therapeutic signatures, and the molecular subtype in GSE31684 cohort.** (A-C) Correlation between Siglec15 and immunomodulators, effector genes of tumor associated immune cells, and inhibitory immune checkpoints in BLCA. (D) Correlation between Siglec15 and enrichment scores of immunotherapy predicted signatures. The asterisks indicated a significant statistical p value calculated with Mann-Whitney U test (*P < 0.05; **P < 0.01; ***P < 0.001). (E) Correlation between Siglec15 and molecular subtype and bladder cancer signatures. (F) Correlation between Siglec15 and enrichment scores of therapeutic signatures, including radiotherapy, targeted therapy.

**Figure S17. Correlations between Siglec15 and the immunological status, the enrichment scores of therapeutic signatures, and the molecular subtype in IMvigor210 cohort.** (A-C) Correlation between Siglec15 and immunomodulators, effector genes of tumor associated immune cells, and inhibitory immune checkpoints in BLCA. (D) Correlation between Siglec15 and enrichment scores of immunotherapy predicted signatures. The asterisks indicated a significant statistical p value calculated with Mann-Whitney U test (*P < 0.05; **P < 0.01; ***P < 0.001). (E) Correlation between Siglec15 and molecular subtype and bladder cancer signatures. (F) Correlation between Siglec15 and enrichment scores of therapeutic signatures, including radiotherapy, targeted therapy.

**Figure S18. Correlations between Siglec15 and the immune signatures in CR subgroup of IMvigor210 cohort.** (A-C) Correlation between Siglec15 and immunomodulators, effector genes of tumor associated immune cells, and inhibitory immune checkpoints in BLCA. (D) Correlation between Siglec15 and enrichment scores of immunotherapy predicted signatures. The asterisks indicated a significant statistical p value calculated with Mann-Whitney U test (*P < 0.05; **P < 0.01; ***P < 0.001).

**Figure S19. Correlations between Siglec15 and the immune signatures in PR subgroup of IMvigor210 cohort.** (A-C) Correlation between Siglec15 and

immunomodulators, effector genes of tumor associated immune cells, and inhibitory immune checkpoints in BLCA. (D) Correlation between Siglec15 and enrichment scores of immunotherapy predicted signatures. The asterisks indicated a significant statistical p value calculated with Mann-Whitney U test (*P < 0.05; **P < 0.01; ***P < 0.001).

**Figure S20. Correlations between Siglec15 and the immune signatures in PD subgroup of IMvigor210 cohort.** (A-C) Correlation between Siglec15 and immunomodulators, effector genes of tumor associated immune cells, and inhibitory immune checkpoints in BLCA. (D) Correlation between Siglec15 and enrichment scores of immunotherapy predicted signatures. The asterisks indicated a significant statistical p value calculated with Mann-Whitney U test (*P < 0.05; **P < 0.01; ***P < 0.001).

**Figure S21. Correlations between Siglec15 and the immune signatures in SD subgroup of IMvigor210 cohort.** (A-C) Correlation between Siglec15 and immunomodulators, effector genes of tumor associated immune cells, and inhibitory immune checkpoints in BLCA. (D) Correlation between Siglec15 and enrichment scores of immunotherapy predicted signatures. The asterisks indicated a significant statistical p value calculated with Mann-Whitney U test (*P < 0.05; **P < 0.01; ***P < 0.001).

**Figure S22. Correlations between Siglec15 and the hyper-progression associated genes, and the predictive accuracy of Siglec15 for molecular subtype in four validation sets.** (A) Correlation between Siglec15 and CNV pattern of hyper-progression associated genes in BLCA. The p value was calculated with Fisher t test. (B) Correlation between Siglec15 and mRNA expression of hyper-progression associated genes in BLCA. The asterisks indicated a significant statistical p value calculated with Mann-Whitney U test (*P < 0.05; **P < 0.01; ***P < 0.001). (C-F) The predictive value of Siglec15 for molecular subtype in four independent validation sets, including an immunotherapy cohort (IMvigor210), a neoadjuvant-chemotherapy cohort(GSE70691), and two general bladder cancer cohorts(GSE31684, GSE48277).

**Figure S23. Role of Siglec15 in predicting molecular subtypes and therapeutic sensitivities in Xiangya cohort.** (A) Correlations between Siglec15 and molecular subtypes and bladder cancer signatures. (B) ROC curves indicated the predictive accuracy of Siglec15 in predicting molecular subtypes. (C) Correlations between Siglec15 and enrichment scores of therapeutic signatures, including radiotherapy, targeted therapy.

**Figure S24. Differentially expressed RNAs between the Siglec15 groups, the immune score groups and the stromal score groups.** (A-F) Differential analysis of RNAs between Siglec15 groups, immune score groups and stromal score groups. The criterias for determining differentially expressed RNAs were set as adjusted P value <

0.01 and |logFC|>1. (G) 1500 common immune-related differential RNAs shown in Venn diagram. (H-I) There is no intersection between RNAs up-expressed among high Siglec15 group and RNAs up-expressed among high immune/stromal score groups. Similarly, there is no intersection between RNAs down-expressed among high Siglec15 group and RNAs down-expressed among high immune/stromal score groups. (J) There is 1010 common RNAs between RNAs down-expressed among high Siglec15 group and RNAs up-expressed among high immune/stromal score groups. (K) There is 490 common RNAs between genes up-expressed among high Siglec15 group and RNAs down-expressed among high immune/stromal score groups.

**Figure S25. GO and KEGG analysis of 1500 common differentially expressed RNAs.** (A-C) Biological process, cellular component, and molecular function of 1500 common differentially expressed RNAs. (D) KEGG analysis of 1500 common differentially expressed RNAs.

**Figure S26. Performance of the IRS RNA-expression profiles in predicting survival in five independent validation cohorts**. (A-E) Survival in high vs. low-IRS patients depicted by KM plots and ROC curves to depict the accuracy of IRS in five bladder cancer cohorts. (E) Survival in high vs. low-IRS patients depicted by KM plots and ROC curves to depict the accuracy of IRS in GSE135222 (lung cancer).

**Figure S27. Performance of the IRS RNA-expression profiles in predicting survival in subgroups of IMvigor210 cohort.** (A-H) Survival in high vs. low-IRS patients depicted by KM plots and ROC curves to depict the accuracy of IRS in eight subgroups of IMvigor210 cohort, including CR/PR subgroup, SD/PD subgroup, IC0 subgroup, IC1 subgroup, IC2 subgroup, TC0 subgroup, TC1 subgroup, TC2 subgroup.

**Figure S28. Performance of the IRS RNA-expression profiles in predicting survival in subgroups of IMvigor210 cohort.** (A-H) Survival in high vs. low-IRS patients depicted by KM plots and ROC curves to depict the accuracy of IRS in eight subgroups of IMvigor210 cohort, including deserted phenotype subgroup, excluded phenotype subgroup, inflamed phenotype subgroup, ECOG score0 subgroup, ECOG score1 subgroup, ECOG score2 subgroup, pre-platinum therapy subgroup, no pre-platinum therapy subgroup.

**Figure S29. IRS predicted the clinical response of cancer immunotherapy.**

(A) Correlation between Siglec15 and IRS. (B-C) Correlations between IRS and the pan-cancer T cell inflamed score, and the inhibitory immune checkpoints. (D-E) Correlations between the IRS and immunomodulators, and the tumor-associated immune cells. (F) Correlations between the IRS and the activities of cancer immunity cycles. (G) Correlations between the IRS and the enrichment score of immunotherapy predicted pathways. The asterisks indicated a significant statistical p-value calculated

with Mann-Whitney U test (*P < 0.05; **P < 0.01; ***P < 0.001).

**Figure S30. Comparisons of the accuracy in predicting ICB response and survival probability between IRS and TIDE algorithms in GSE78220.**

(A) The accuracy of two algorithms in predicting ICB response. (B-C) Survival in high vs. low TIDE, and in high vs. low IRS patients depicted by KM plots. (D-E) ROC curves to depict the accuracy of TIDE and IRS in predicting survival probability.

**Figure S31. Comparisons of the accuracy in predicting ICB response and survival probability between IRS and TIDE algorithms in GSE91061.**

(A) The accuracy of two algorithms in predicting ICB response. (B-C) Survival in high vs. low TIDE, and in high vs. low IRS patients depicted by KM plots. (D-E) ROC curves to depict the accuracy of TIDE and IRS in predicting survival probability.

**Figure S32. Comparisons of the accuracy in predicting ICB response and survival probability between IRS and TIDE algorithms in PMID29301960.**

(A) The accuracy of two algorithms in predicting ICB response. (B-C) Survival in high vs. low TIDE, and in high vs. low IRS patients depicted by KM plots. (D-E) ROC curves to depict the accuracy of TIDE and IRS in predicting survival probability.

**Figure S33. Comparisons of the accuracy in predicting ICB response and survival probability between IRS and TIDE algorithms in IMvigor210.**

(A) The accuracy of two algorithms in predicting ICB response. (B-C) Survival in high vs. low TIDE, and in high vs. low IRS patients depicted by KM plots. (D-E) ROC curves to depict the accuracy of TIDE and IRS in predicting survival probability.

**Table S1.** Characters of data sets; Abbreviations of cancer types; Cox analysis in TCGA BLCA cohort.

**Table S2**. Correlations between Siglec15 and 122 immunomodulators, PD-L1, PD-1,CTLA-3, LAG-3.

**Table S3.** Comparisons of the cancer-immunity cycle activity between Siglec15 groups.

**Table S4.** Infiltration level of tumor associated immune cells in BLCA estimated by using different algorithms.

**Table S5.** Comparisons of the effector genes of tumor associated immune cells between Siglec15 groups.

**Table S6.** Comparisons of the inhibitory immune checkpoints between Siglec15 groups.

**Table S7.** The pan-cancer T cell inflamed score of TCGA BLCA patients.

**Table S8.** The CNV patterns of hyper-progression associated genes in BLCA.

**Table S9.** Detailed information of immunotherapy predicted signatures, bladder cancer signatures and other therapeutic signatures.

**Table S10.** Comparisons of therapeutic sensitivity of drug genes used in BCLA between Siglec15 groups.

**Table S11.** Molecular subtypes of patients in TCGA BLCA cohort.

**Table S12.** Expression of Siglec15, PD-L1, and CD8 in the clinical validation set.

**Table S13.** Comparisons of the enrichment score of therapeutic predicted pathways between Siglec15 groups.

**Table S14.** Correlations between Siglec15 and immunomodulators in Xiangya cohort.

**Table S15.** 1500 common differentially expressed RNAs.

**Table S16.** GO, KEGG analysis of 1500 common differentially expressed RNAs.

**Table S17.** Protein-protein interaction analysis of 1500 differentially expressed RNAs.

**Table S18.** Univariate cox analysis of 1500 common differentially expressed RNAs.
