# Supplementary figures and images for: Siglec15 shapes a non-inflamed tumor microenvironment and predicts the molecular subtype in bladder cancer
Source: Theranostics. 2021 Jan 1;11(7):3089–108. doi: 10.7150/thno.53649 (PMC7847675; doi:10.7150/thno.53649)

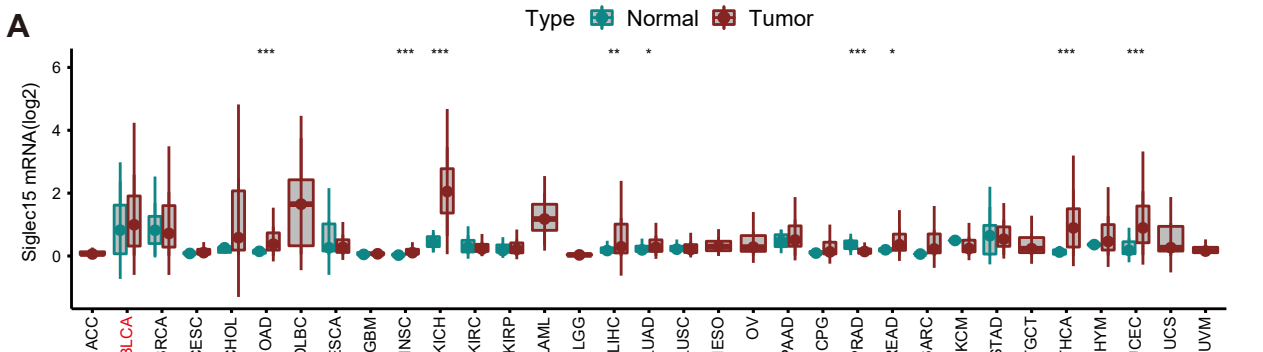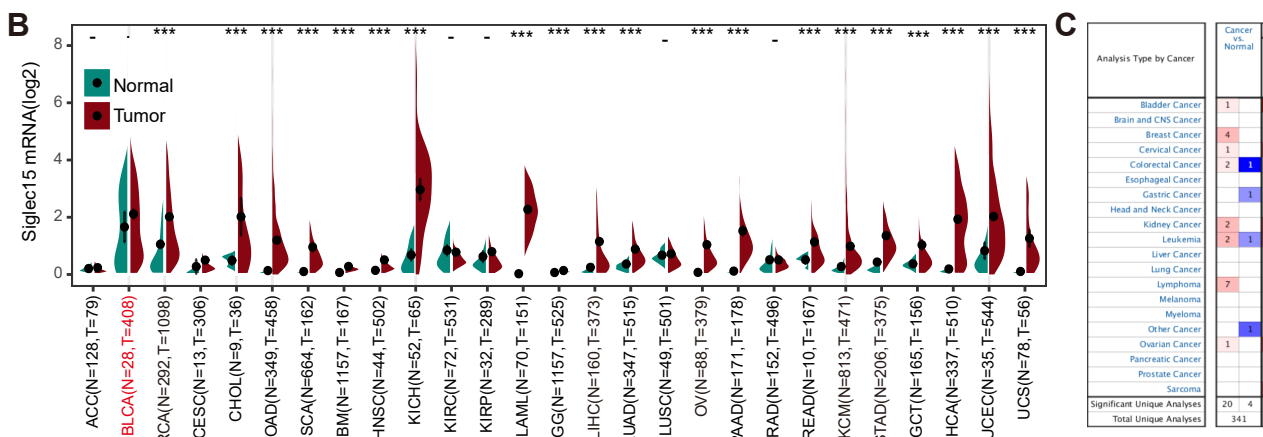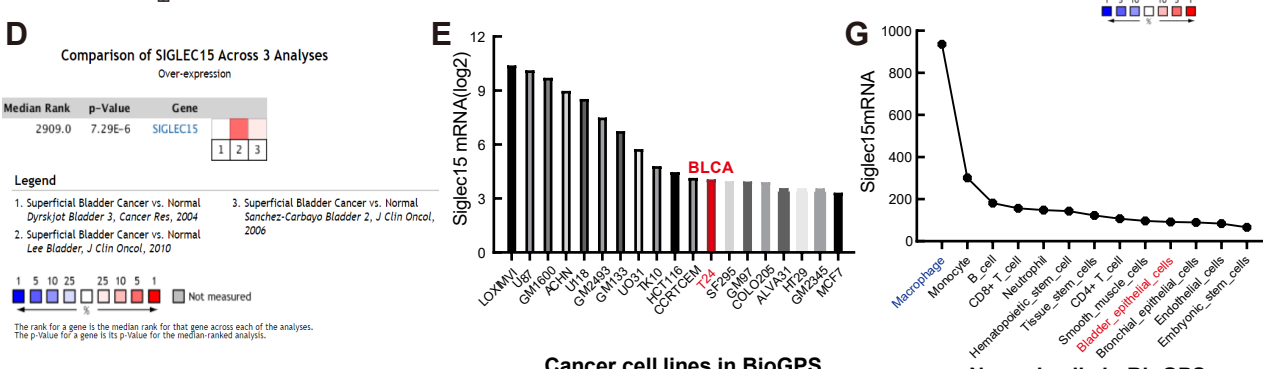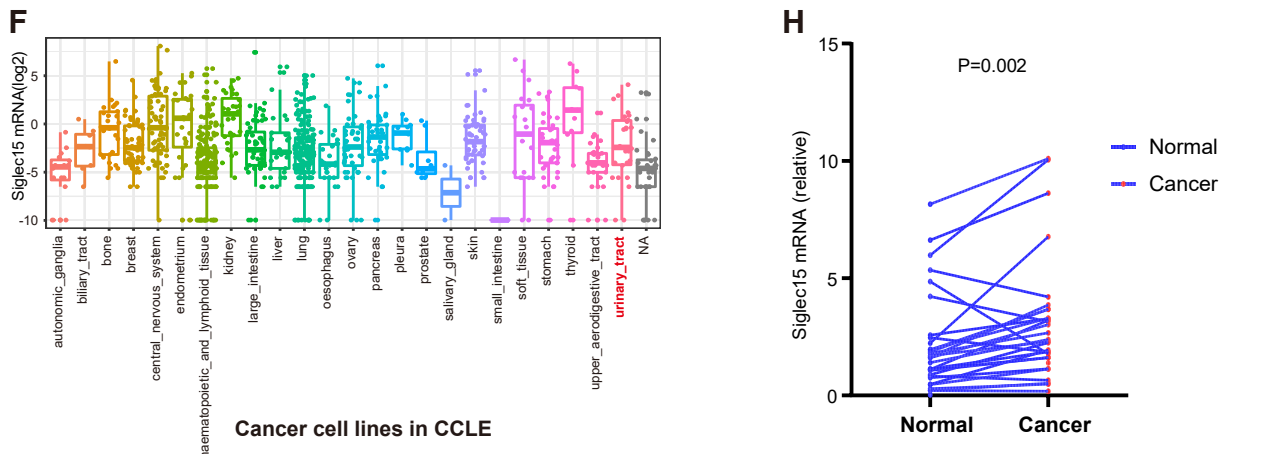

Supplement: Supplementary file 2 — Supplementary figures 1-19. [file thnov11p3089s2.zip › Figure S1.pdf]

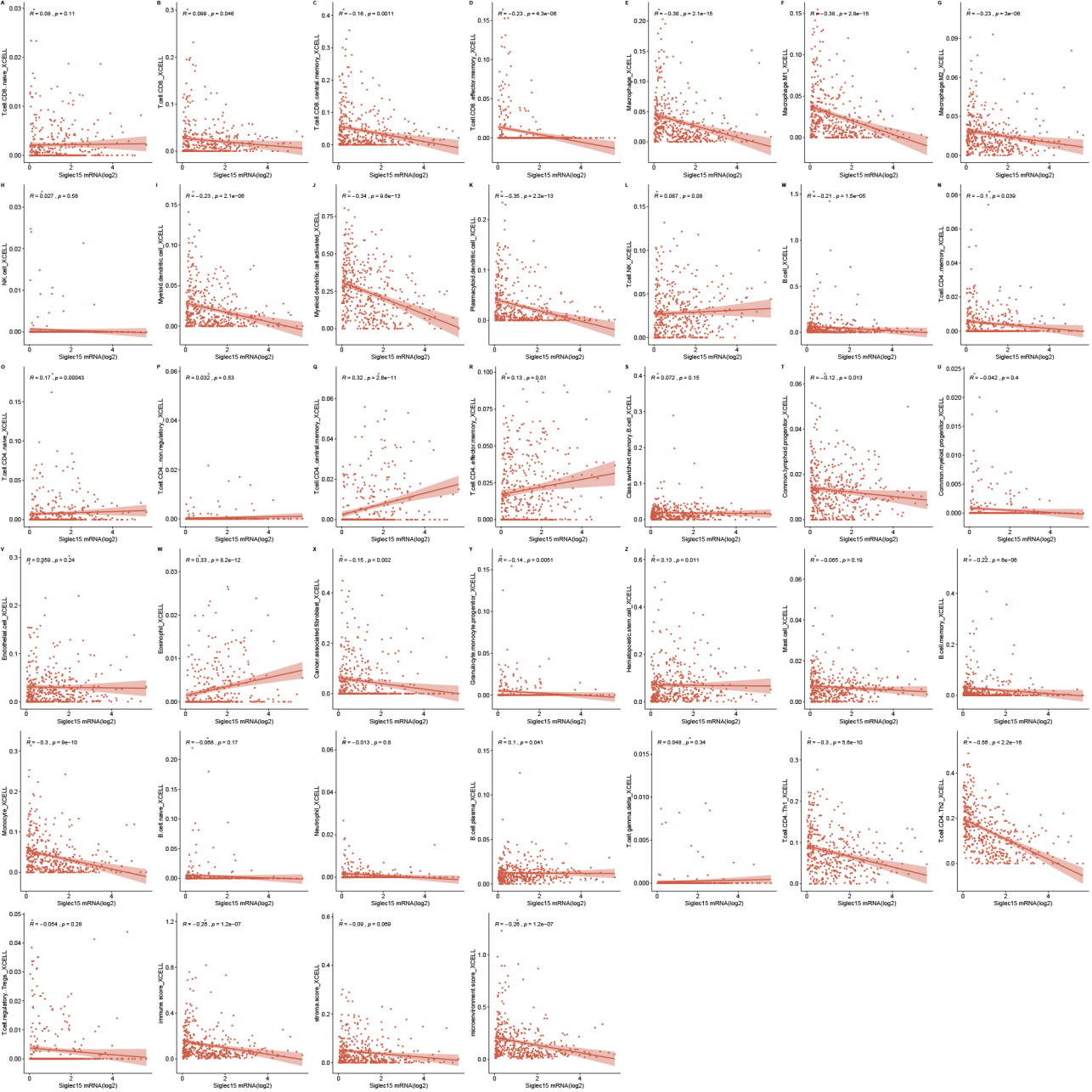

Supplement: Supplementary file 2 — Supplementary figures 1-19. [file thnov11p3089s2.zip › Figure S10.pdf]

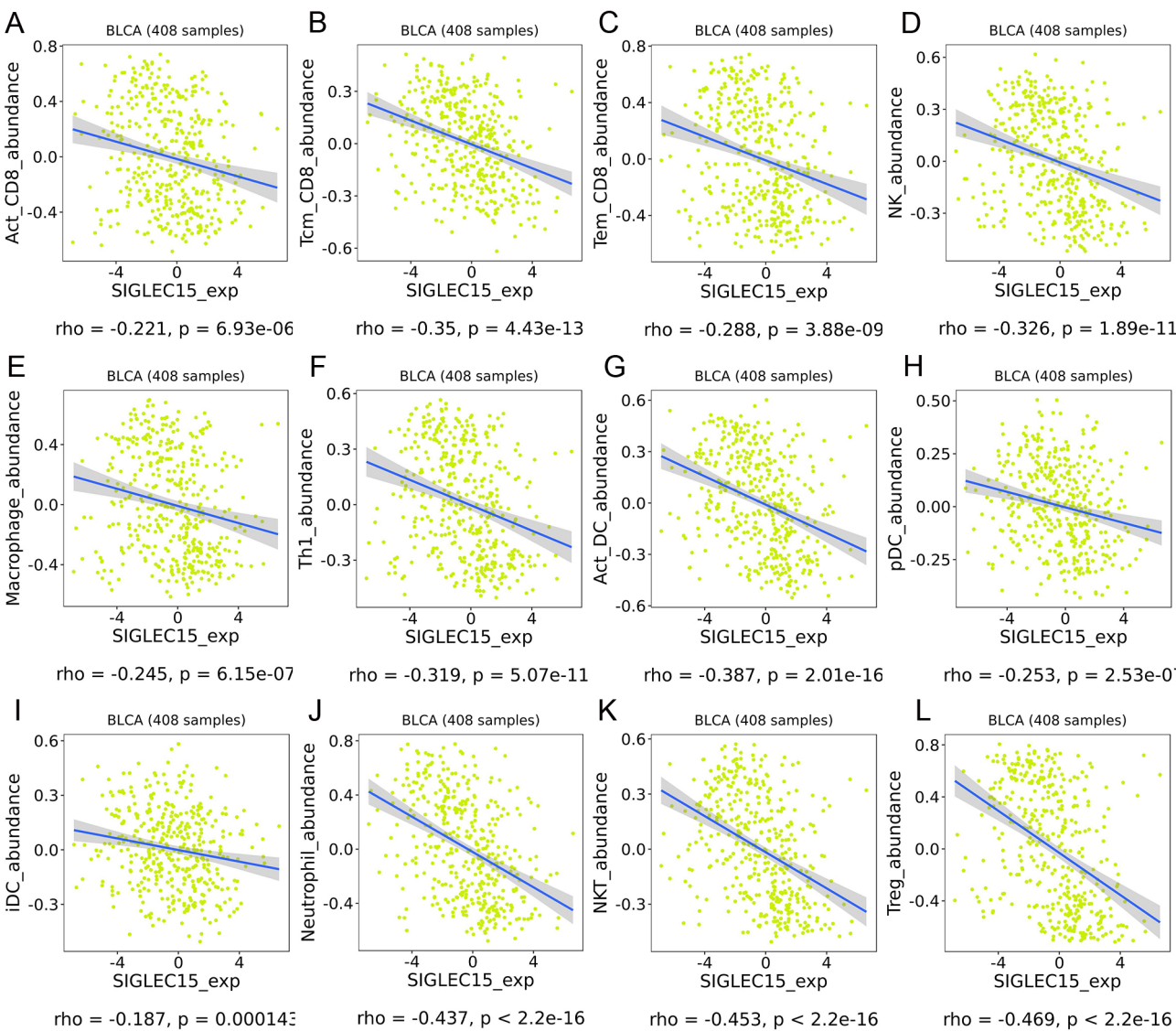

Supplement: Supplementary file 2 — Supplementary figures 1-19. [file thnov11p3089s2.zip › Figure S11.pdf]

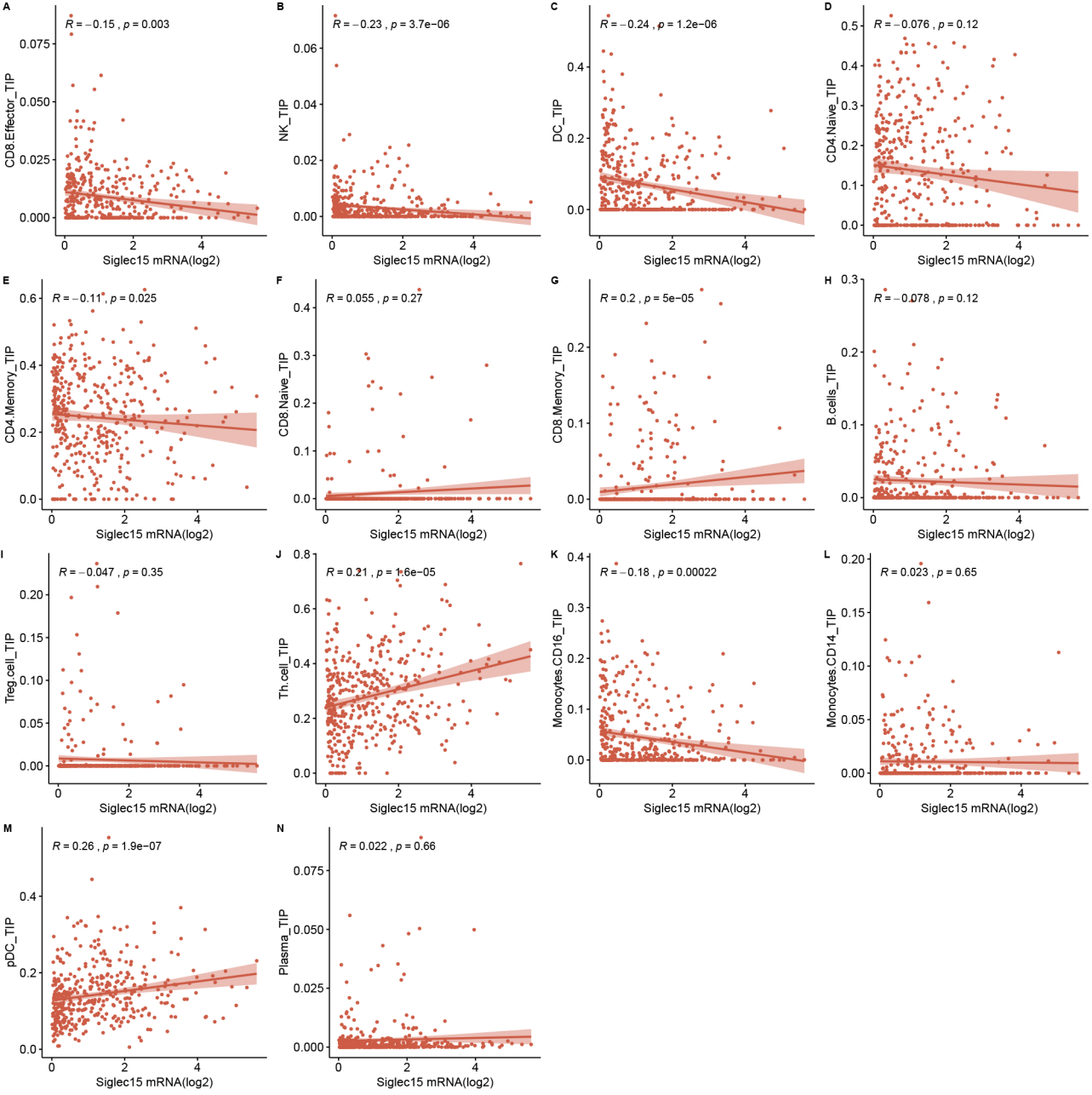

Supplement: Supplementary file 2 — Supplementary figures 1-19. [file thnov11p3089s2.zip › Figure S12.pdf]

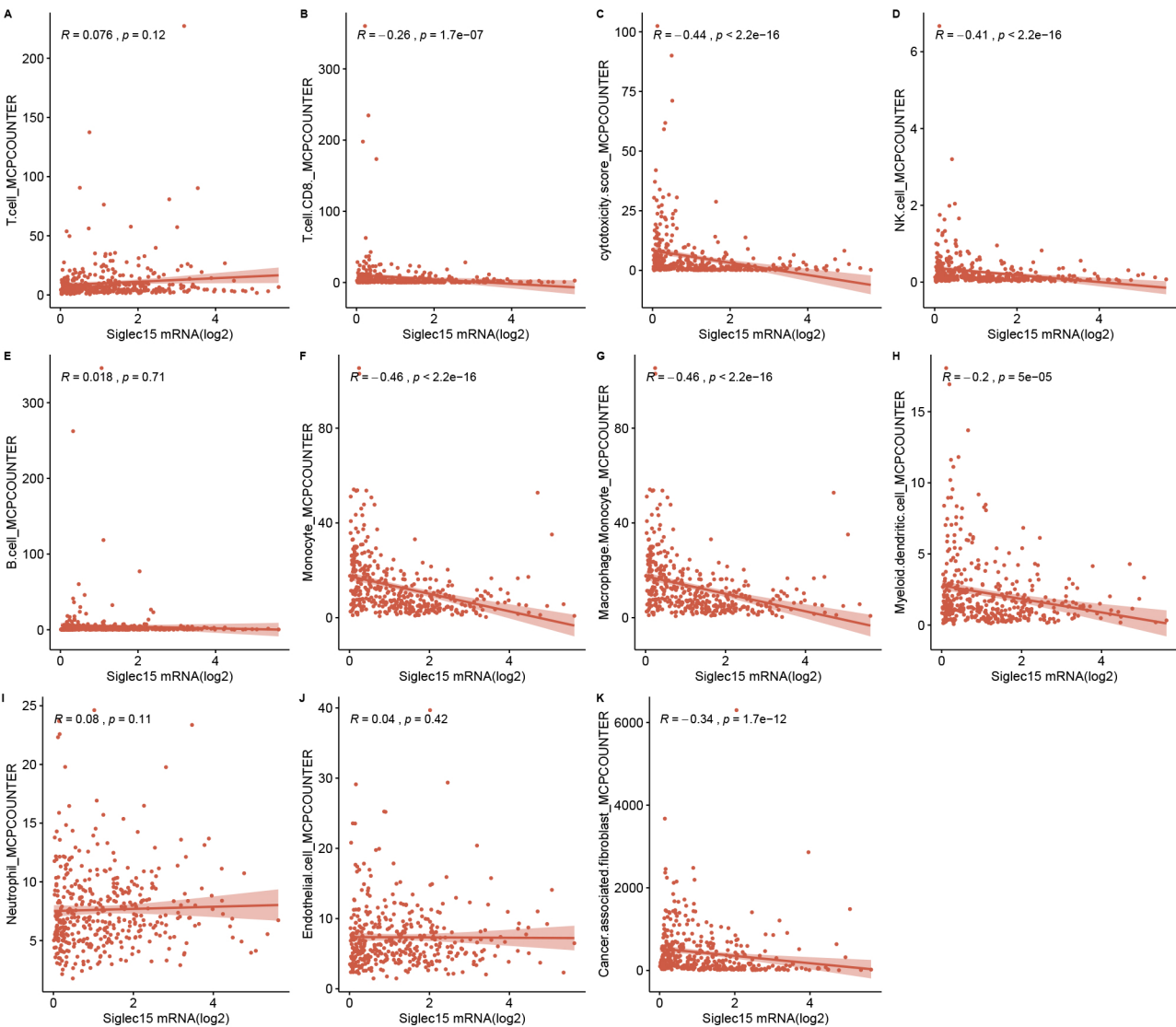

Supplement: Supplementary file 2 — Supplementary figures 1-19. [file thnov11p3089s2.zip › Figure S13.pdf]

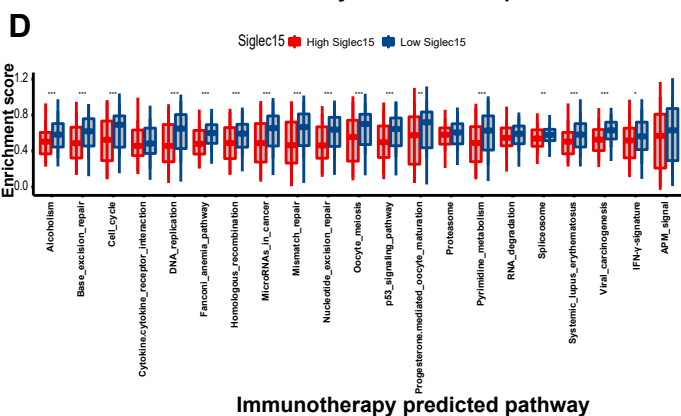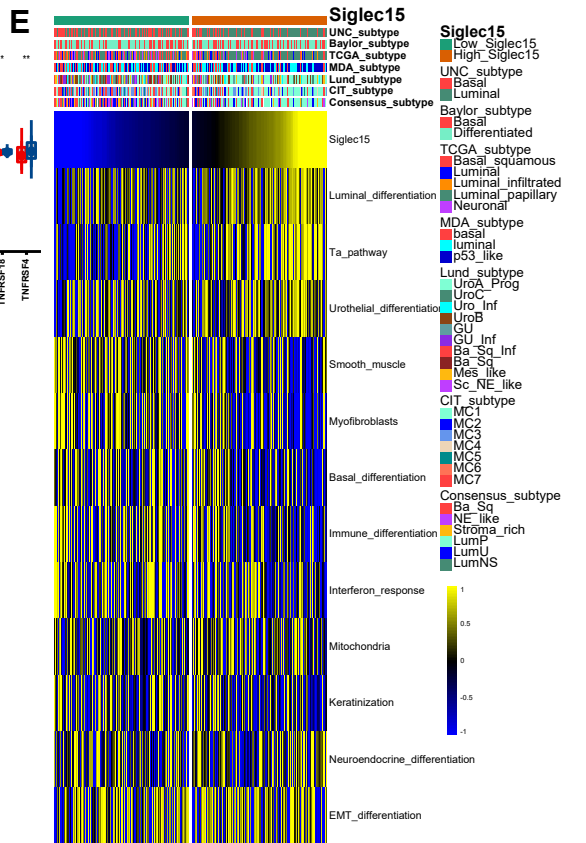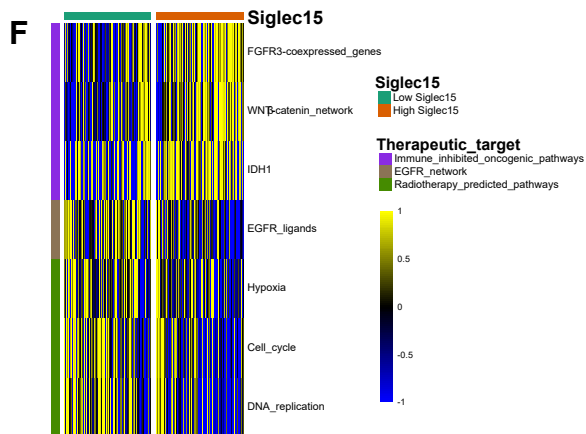

Supplement: Supplementary file 2 — Supplementary figures 1-19. [file thnov11p3089s2.zip › Figure S15.pdf]

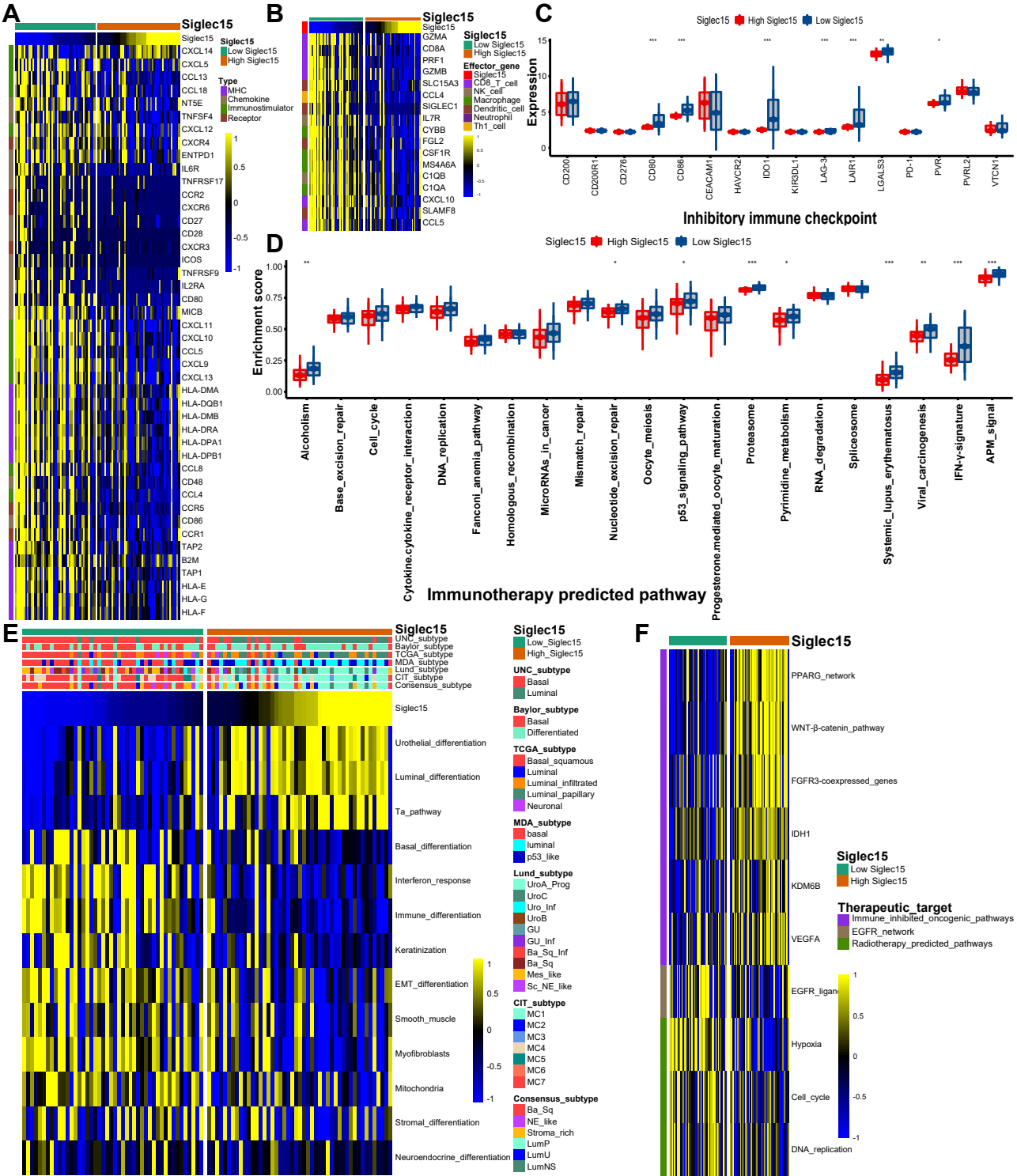

Supplement: Supplementary file 2 — Supplementary figures 1-19. [file thnov11p3089s2.zip › Figure S16.pdf]

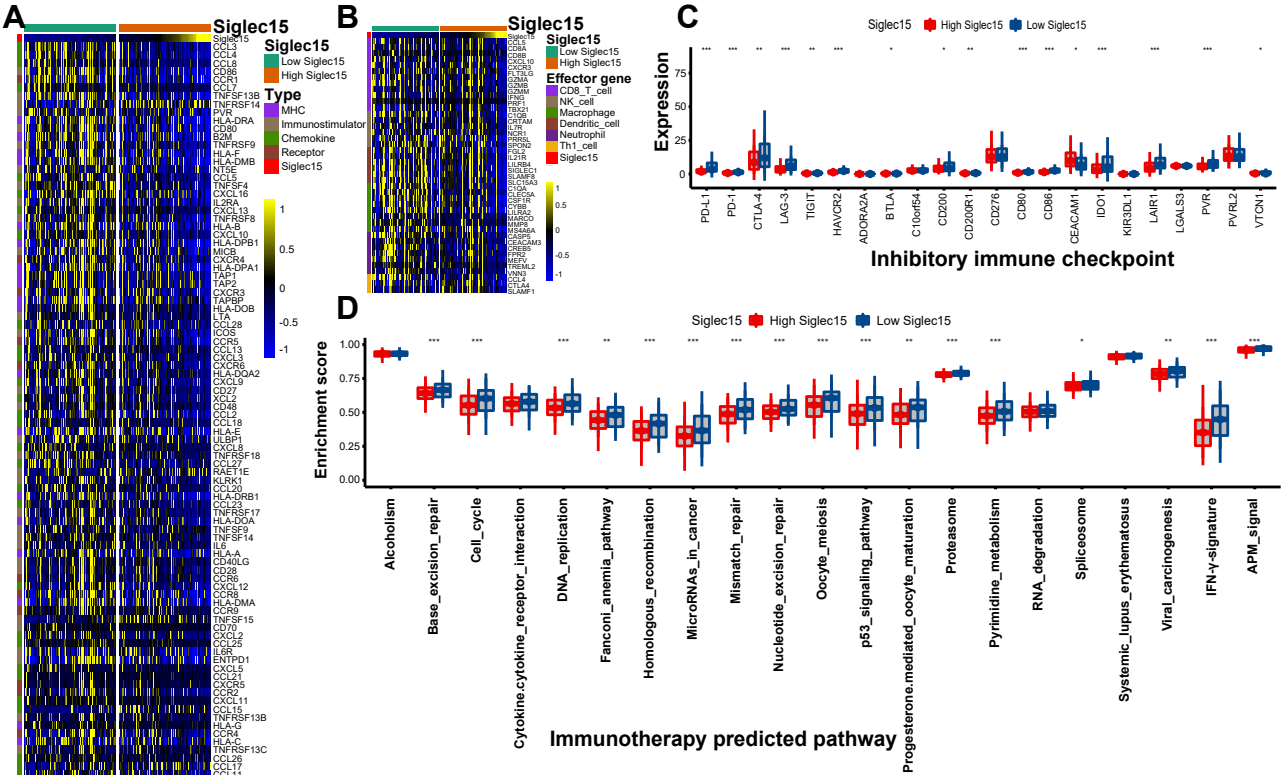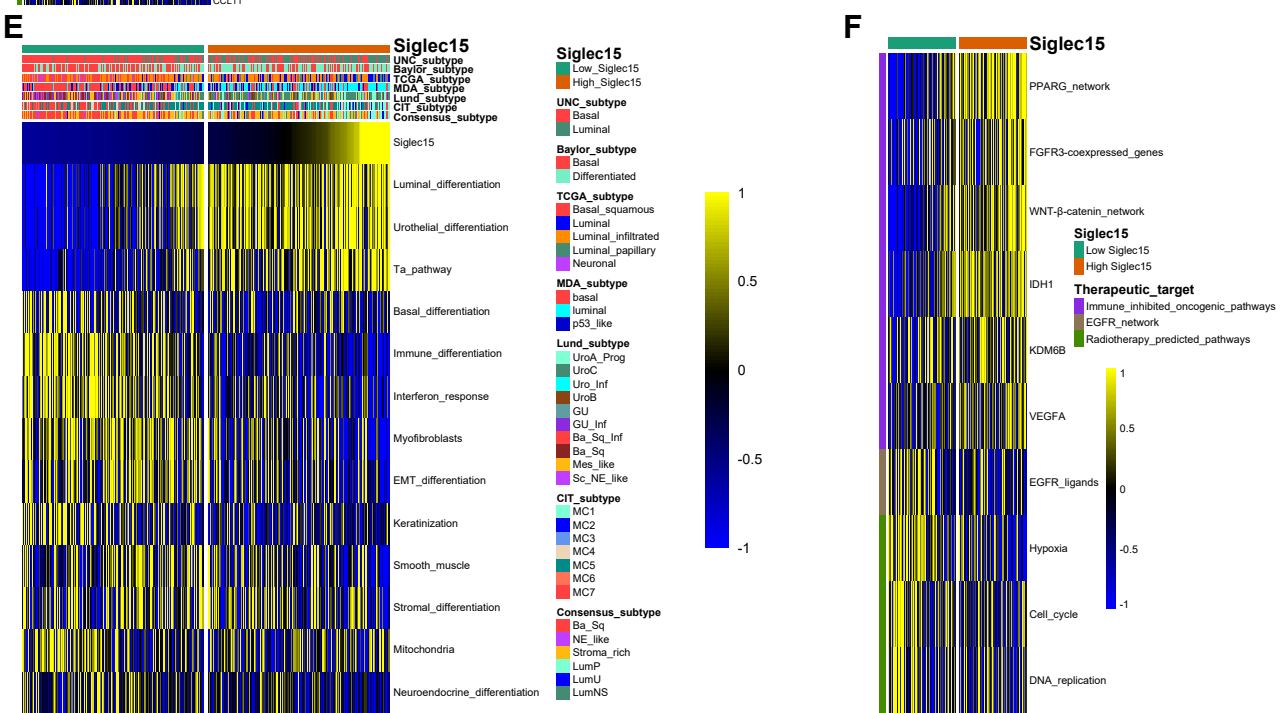

Supplement: Supplementary file 2 — Supplementary figures 1-19. [file thnov11p3089s2.zip › Figure S17.pdf]

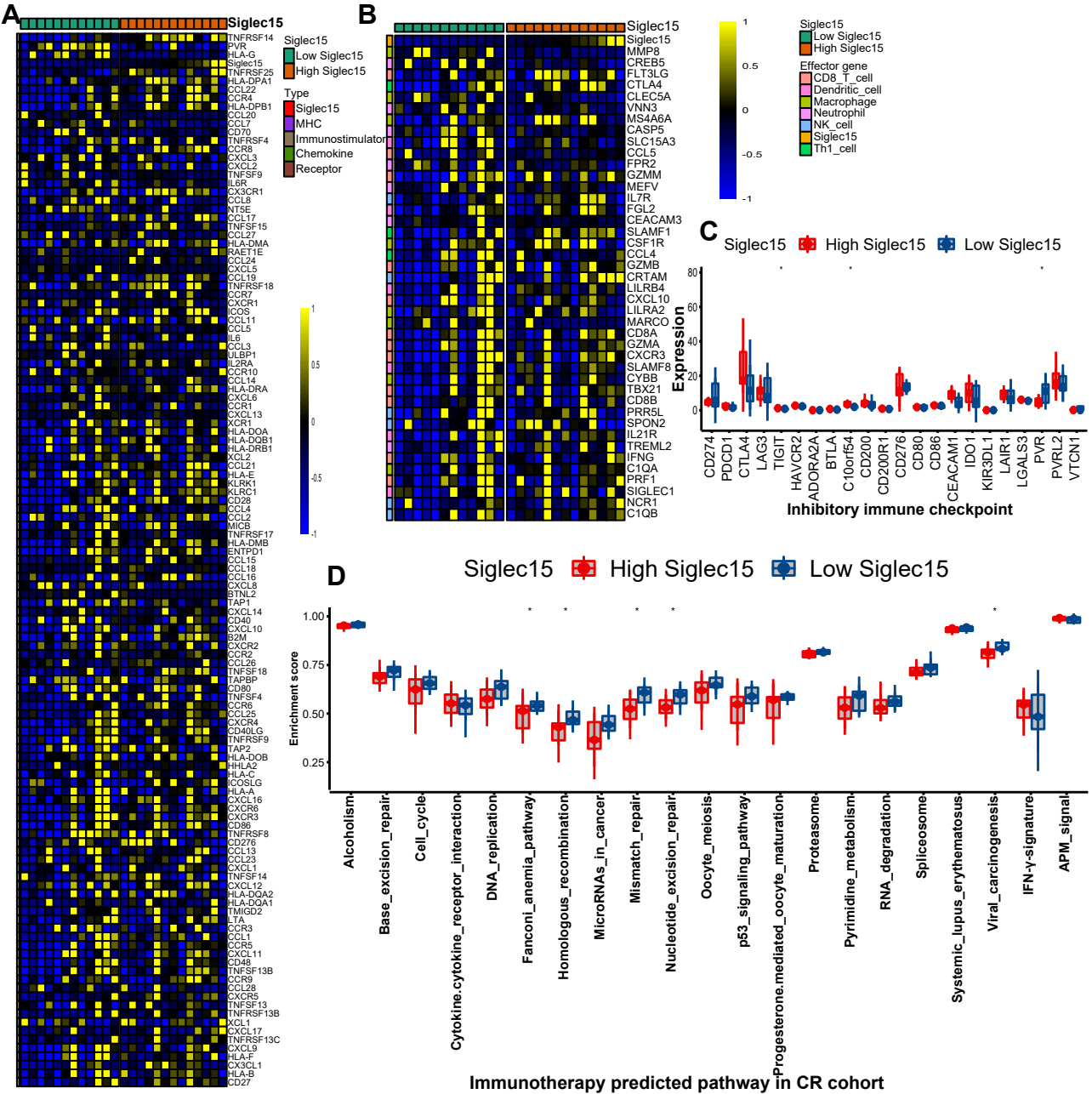

Supplement: Supplementary file 2 — Supplementary figures 1-19. [file thnov11p3089s2.zip › Figure S18.pdf]

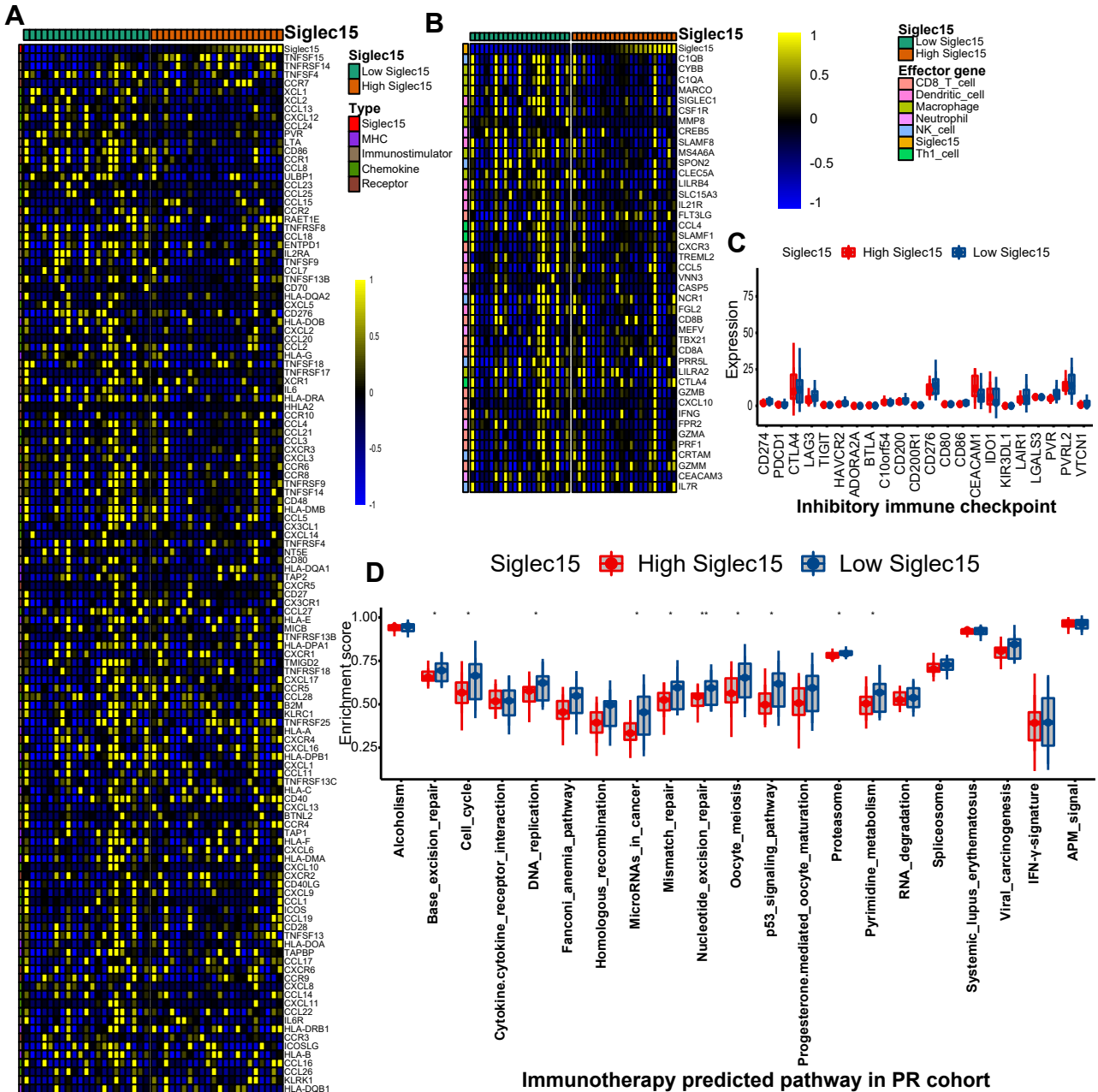

Supplement: Supplementary file 2 — Supplementary figures 1-19. [file thnov11p3089s2.zip › Figure S19.pdf]

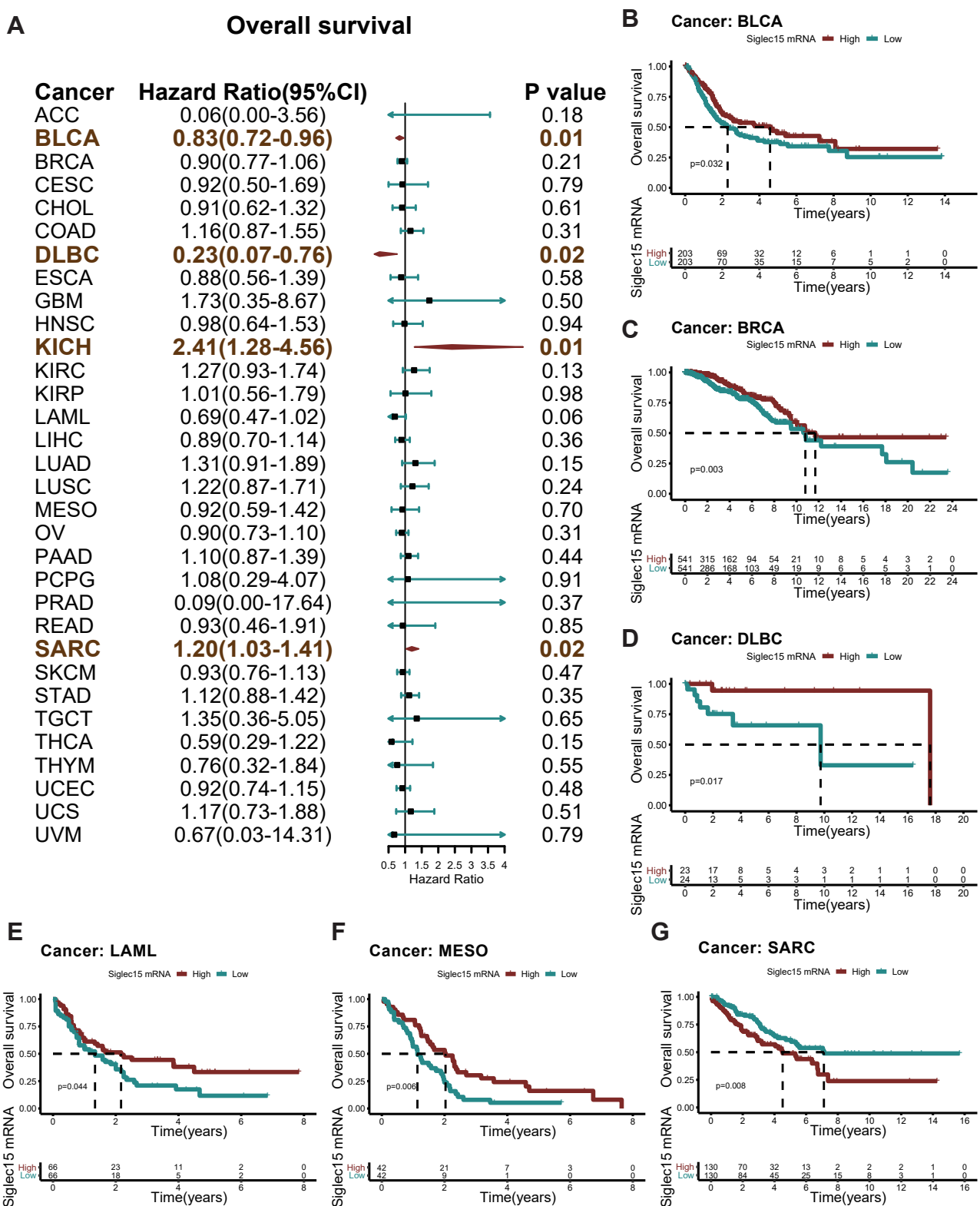

Supplement: Supplementary file 2 — Supplementary figures 1-19. [file thnov11p3089s2.zip › Figure S2.pdf]

# Disease specific survival

**A**

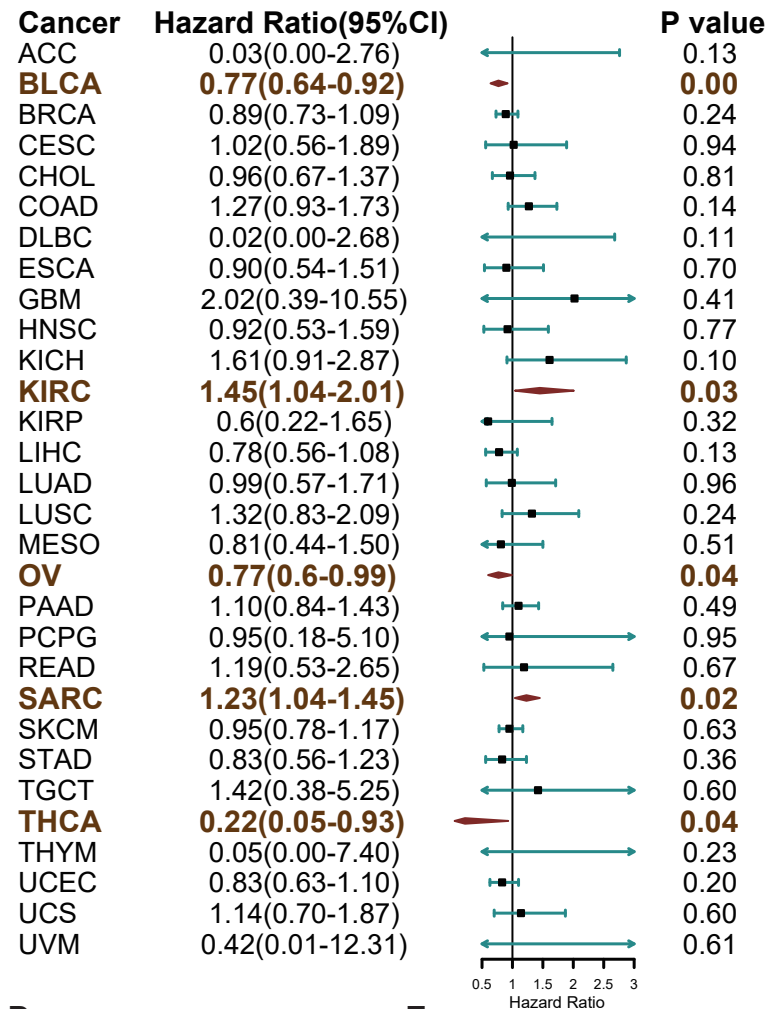

**B**

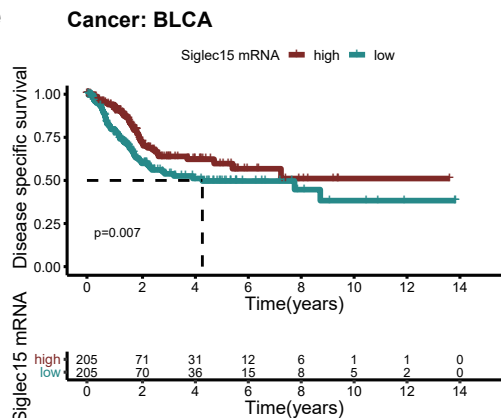

**C**

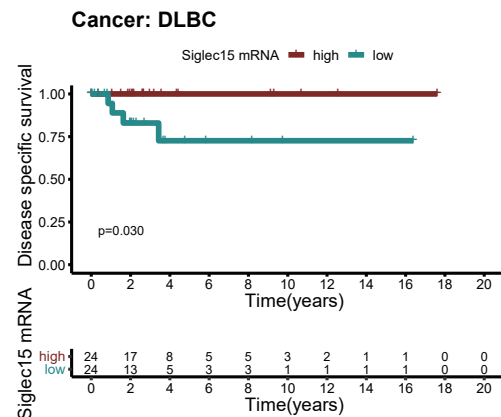

**D**

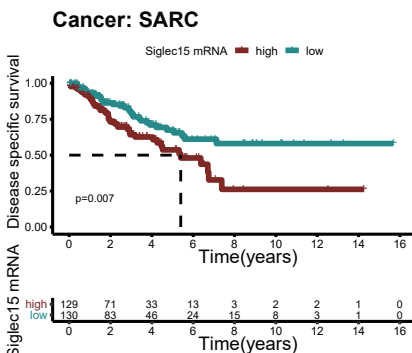

**E**

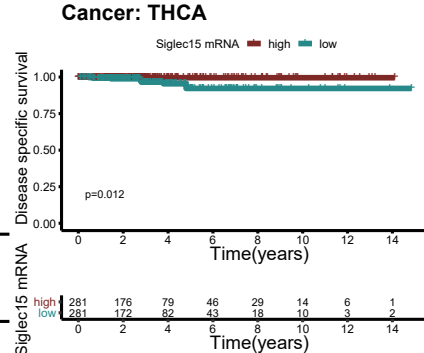

**F**

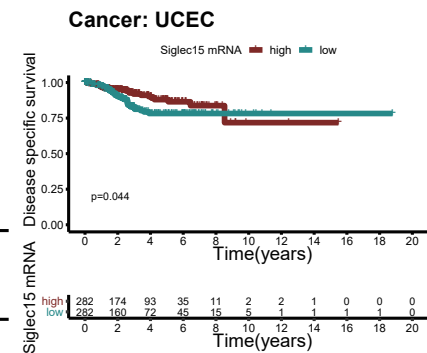

Supplement: Supplementary file 2 — Supplementary figures 1-19. [file thnov11p3089s2.zip › Figure S3.pdf]

# A Progression free survival

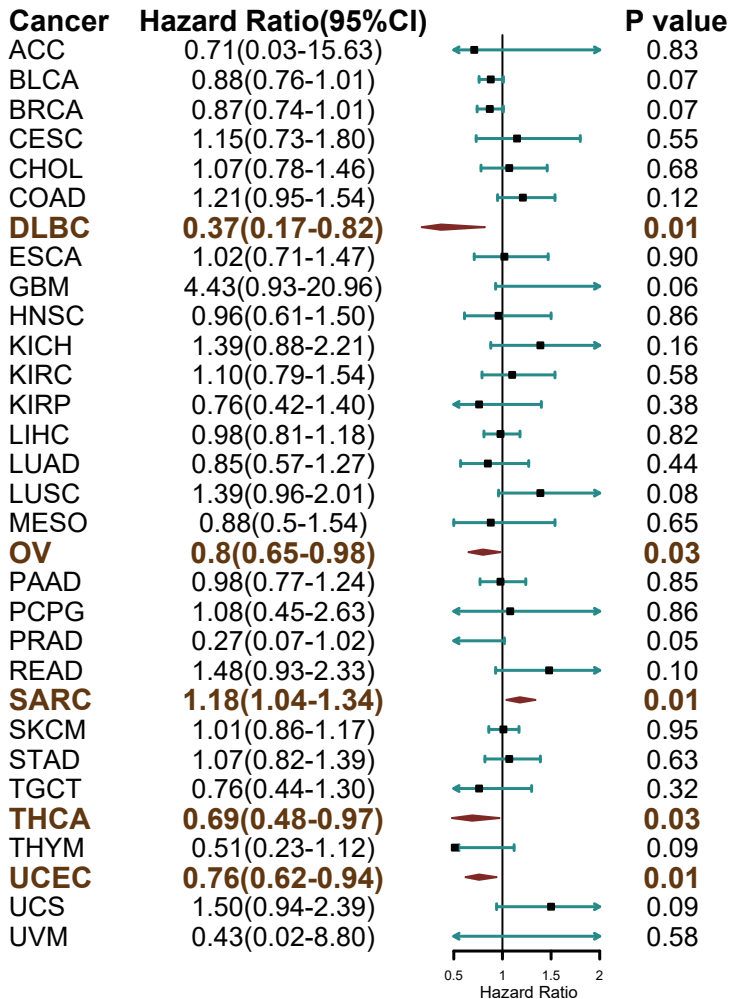

# B

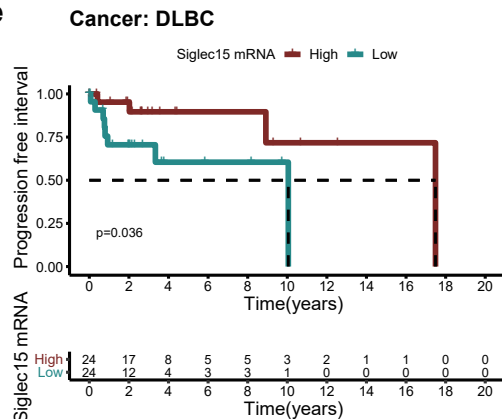

# C

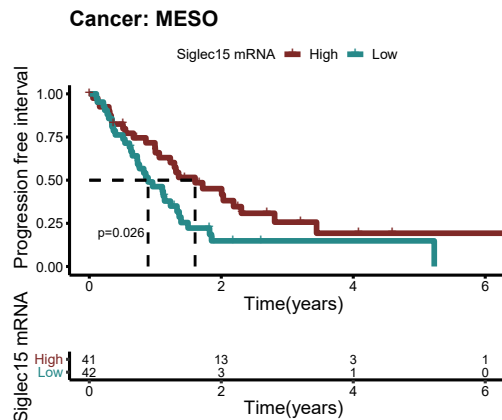

# D

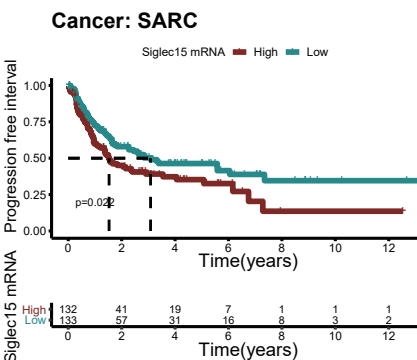

# E

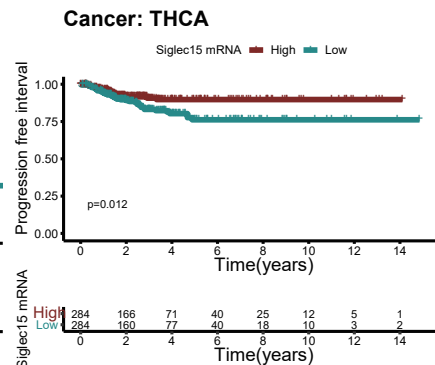

# F

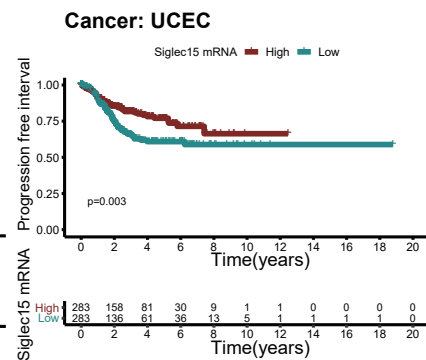

Supplement: Supplementary file 2 — Supplementary figures 1-19. [file thnov11p3089s2.zip › Figure S4.pdf]

**A**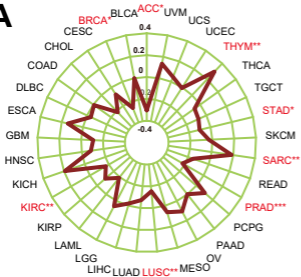**B**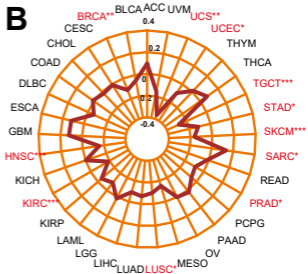

Supplement: Supplementary file 2 — Supplementary figures 1-19. [file thnov11p3089s2.zip › Figure S5.pdf]

**A****Siglec15 CNV Pattern**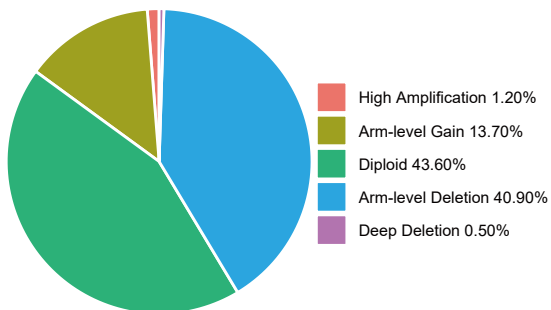**B**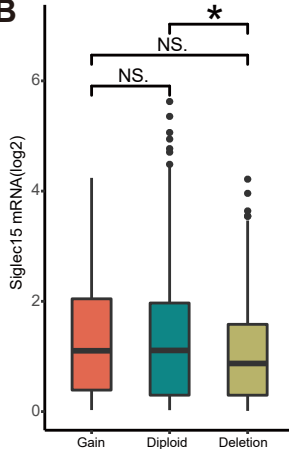**C**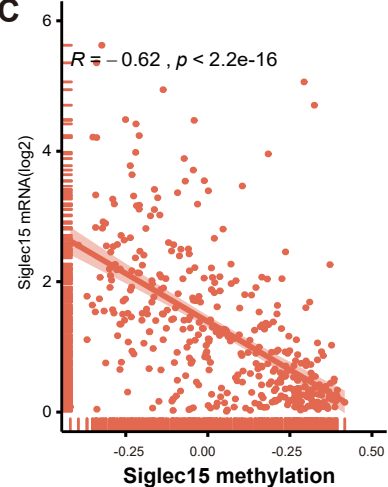**D**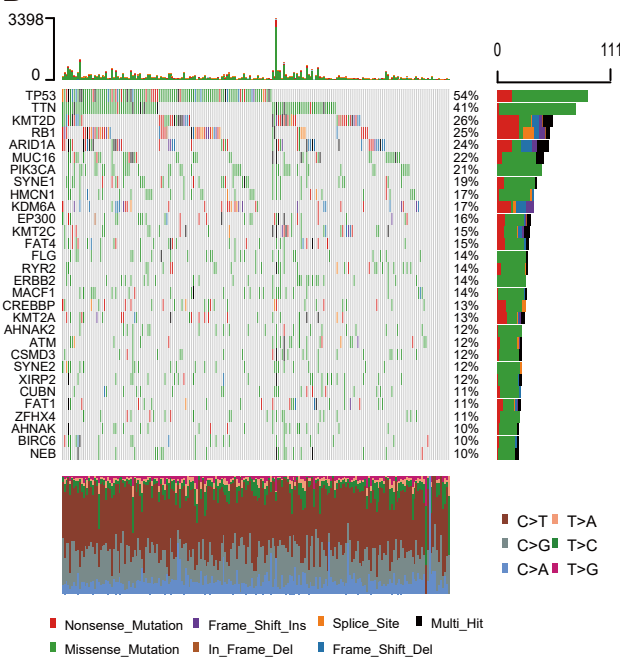**E**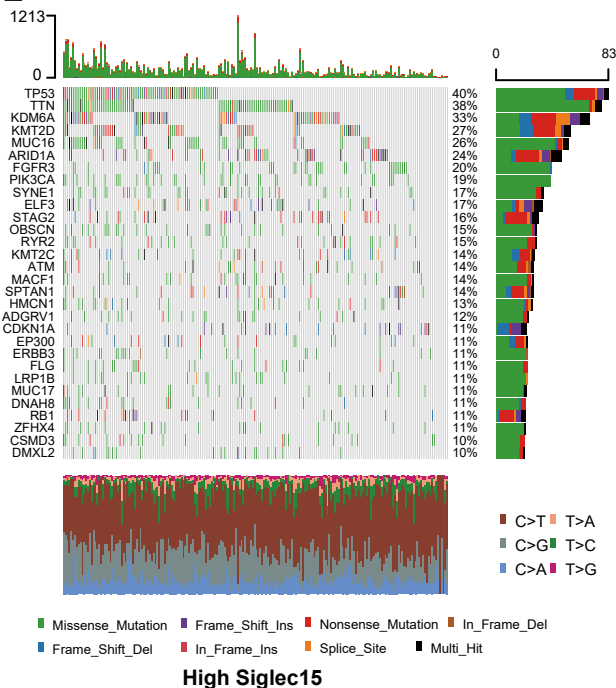**F**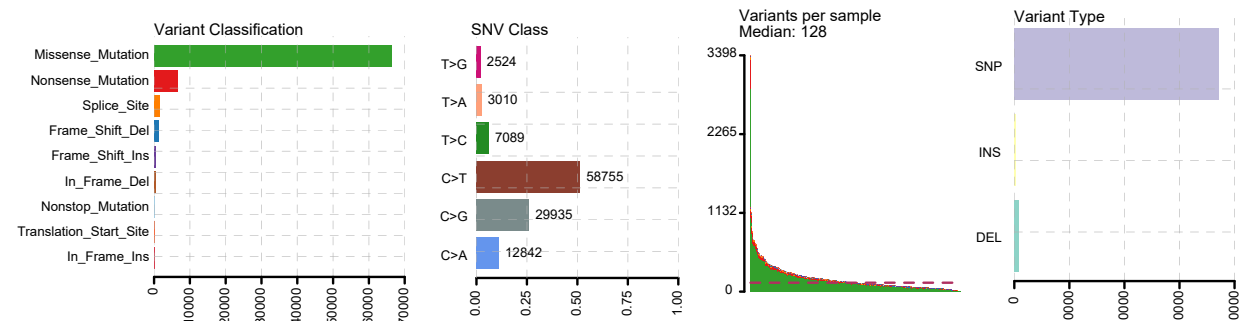

Supplement: Supplementary file 2 — Supplementary figures 1-19. [file thnov11p3089s2.zip › Figure S6.pdf]

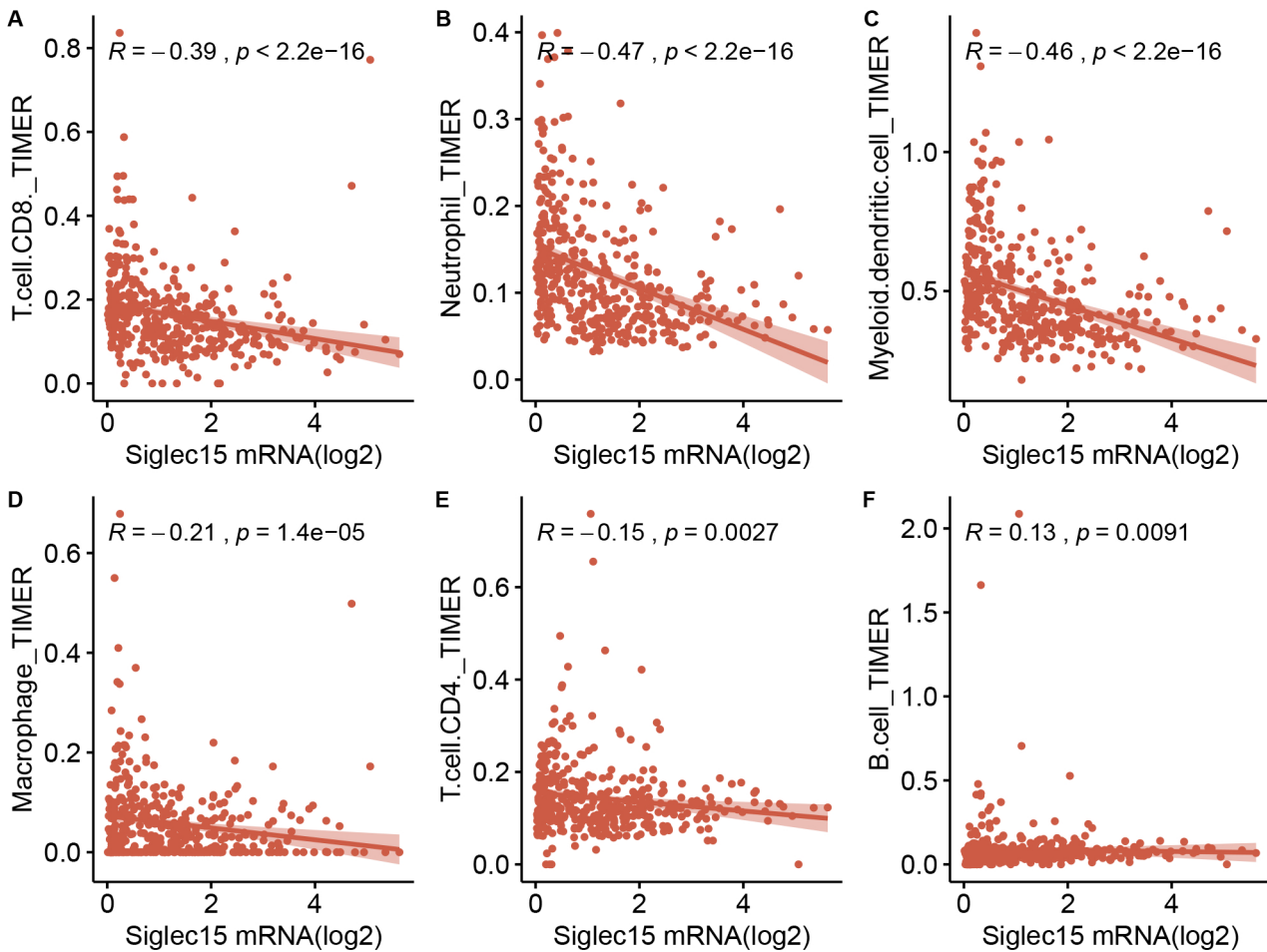

Supplement: Supplementary file 2 — Supplementary figures 1-19. [file thnov11p3089s2.zip › Figure S7.pdf]

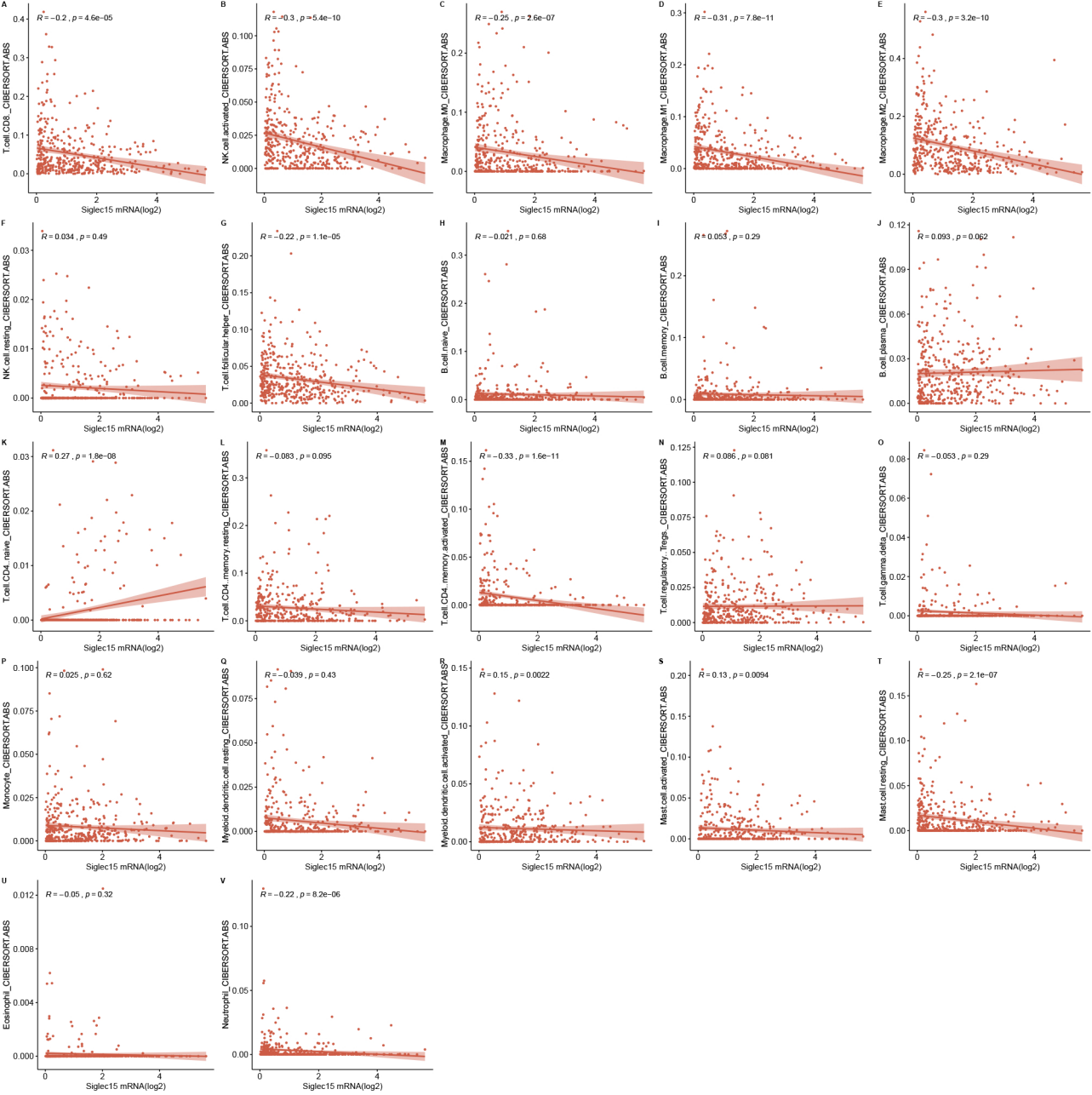

Supplement: Supplementary file 2 — Supplementary figures 1-19. [file thnov11p3089s2.zip › Figure S8.pdf]

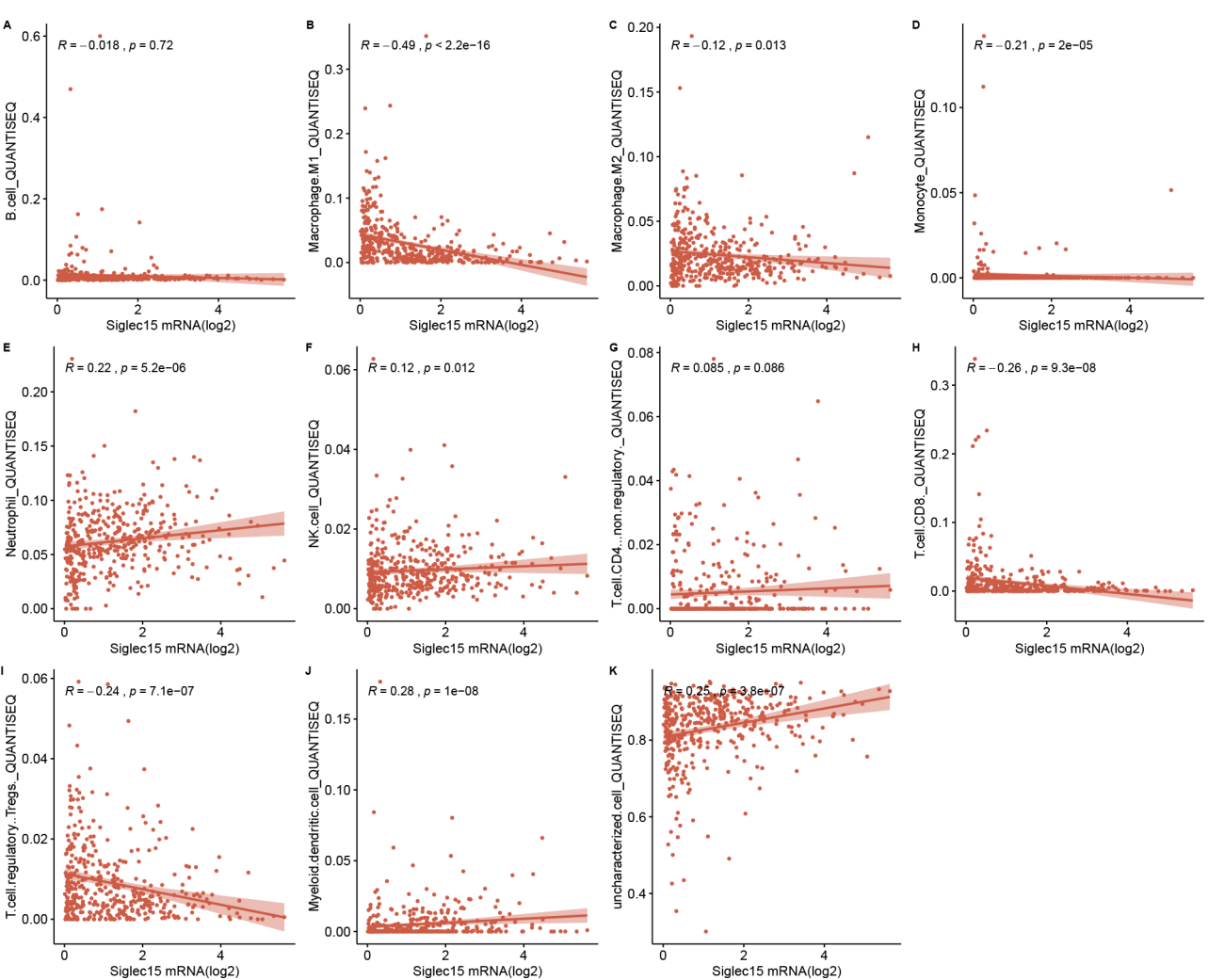

Supplement: Supplementary file 2 — Supplementary figures 1-19. [file thnov11p3089s2.zip › Figure S9.pdf]

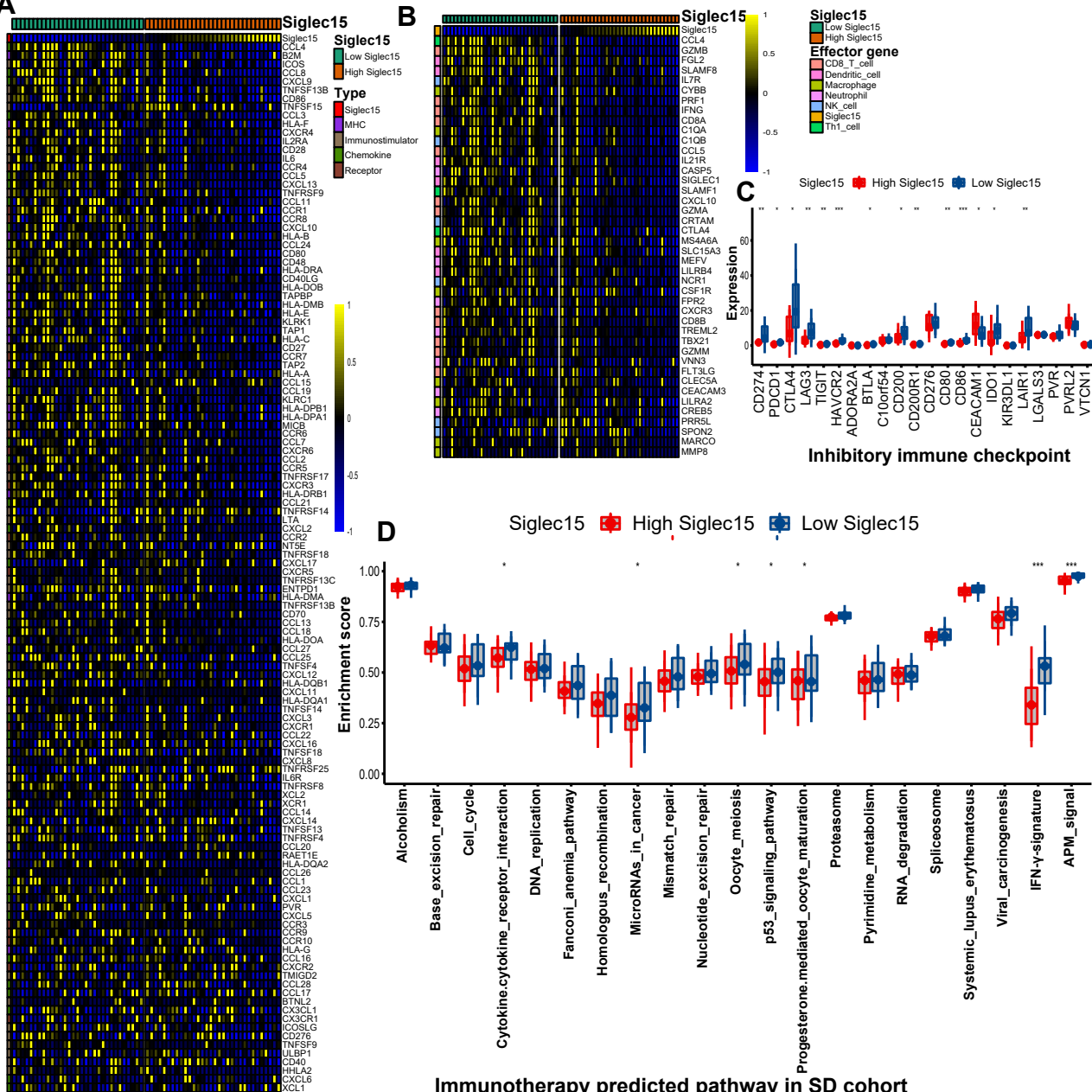

Supplement: Supplementary file 3 — Supplementary figures 20-33. [file thnov11p3089s3.zip › Figure S21.pdf]

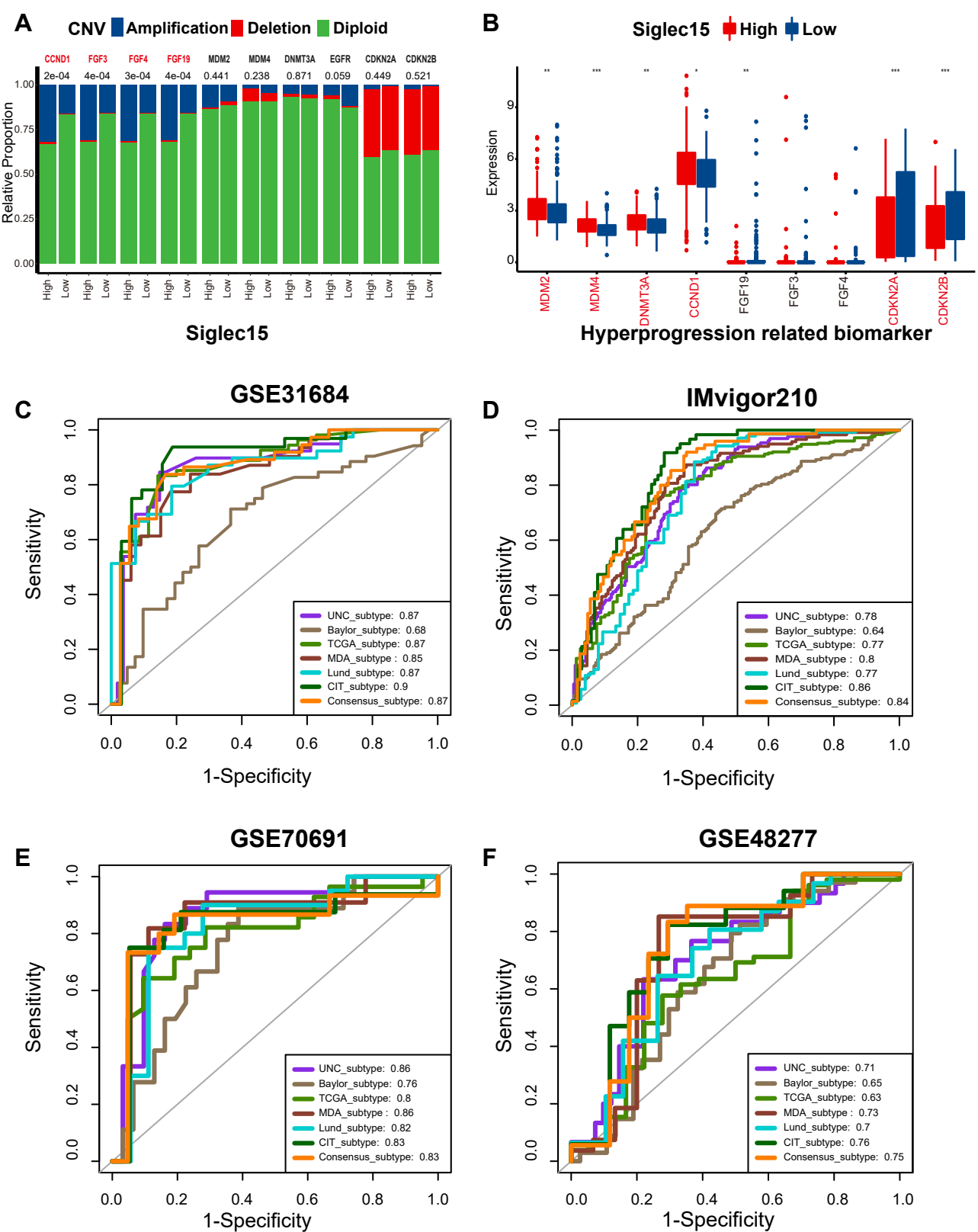

Supplement: Supplementary file 3 — Supplementary figures 20-33. [file thnov11p3089s3.zip › Figure S22.pdf]

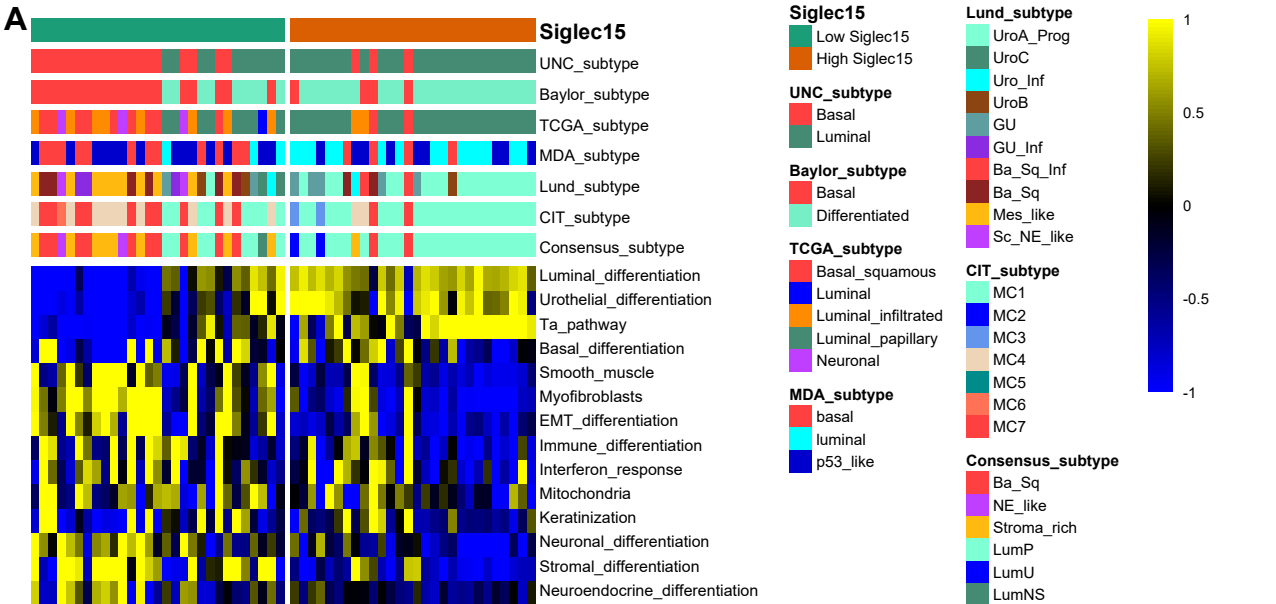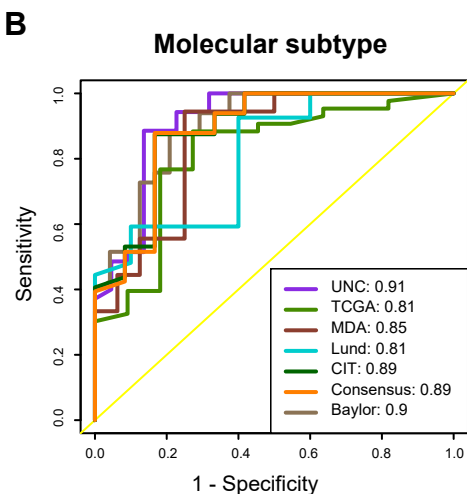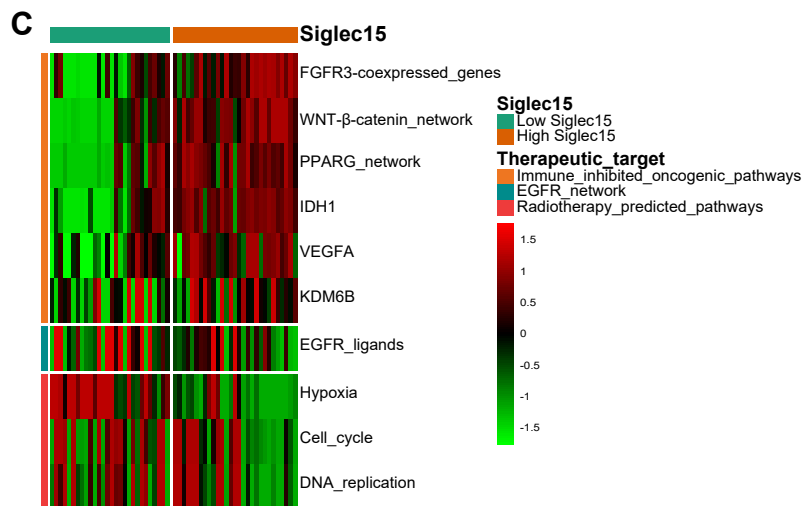

Supplement: Supplementary file 3 — Supplementary figures 20-33. [file thnov11p3089s3.zip › Figure S23.pdf]

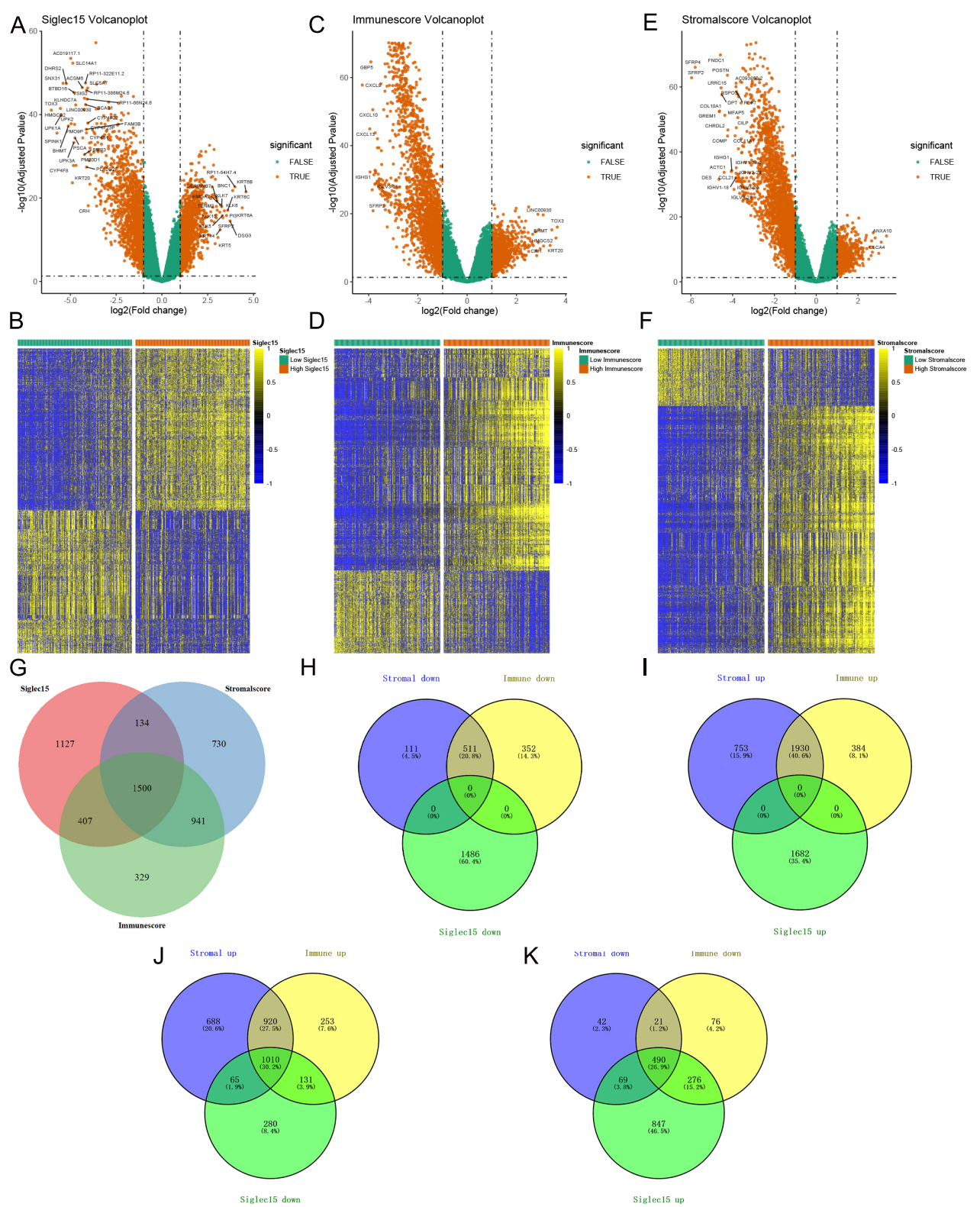

Supplement: Supplementary file 3 — Supplementary figures 20-33. [file thnov11p3089s3.zip › Figure S24.pdf]

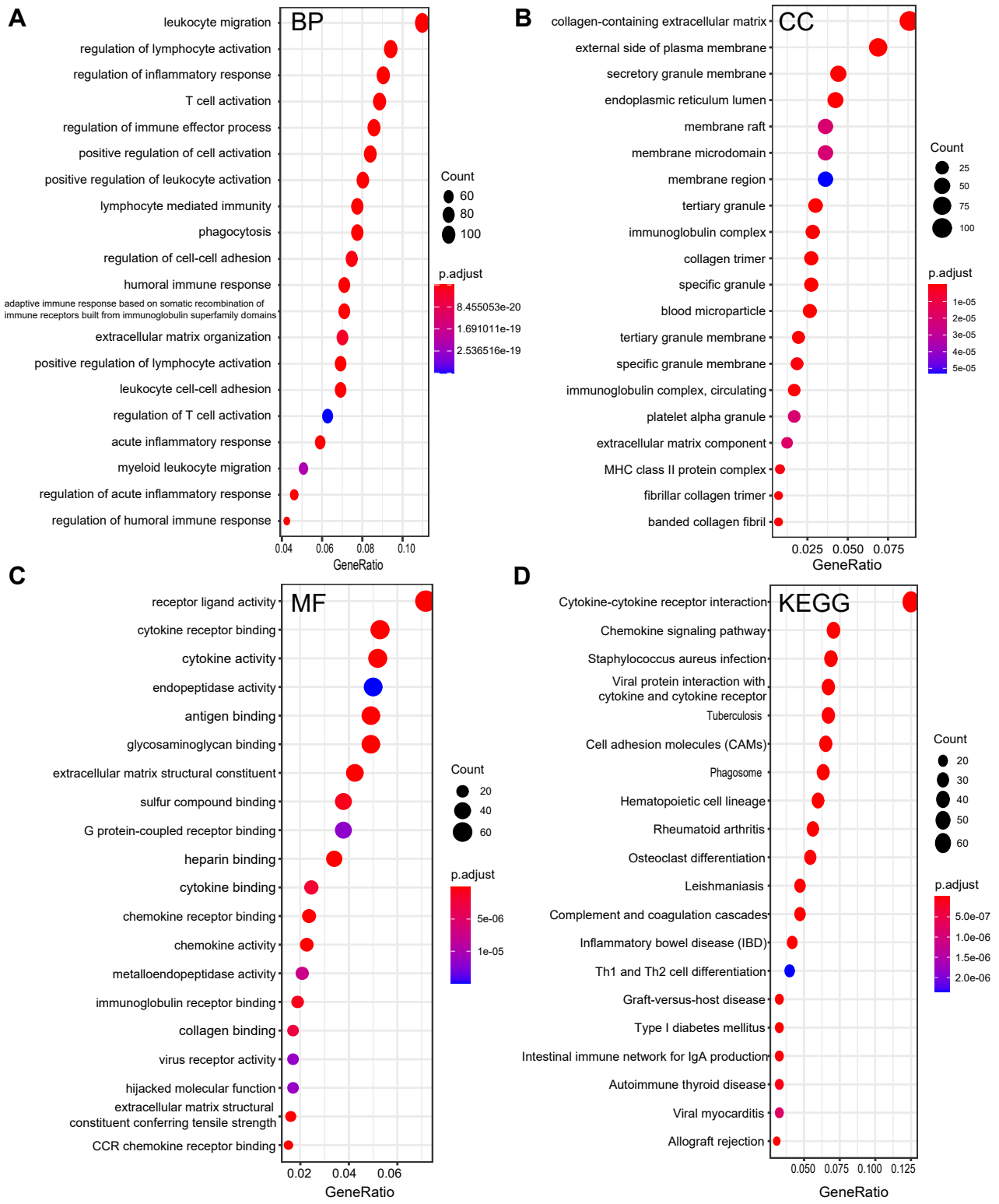

Supplement: Supplementary file 3 — Supplementary figures 20-33. [file thnov11p3089s3.zip › Figure S25.pdf]

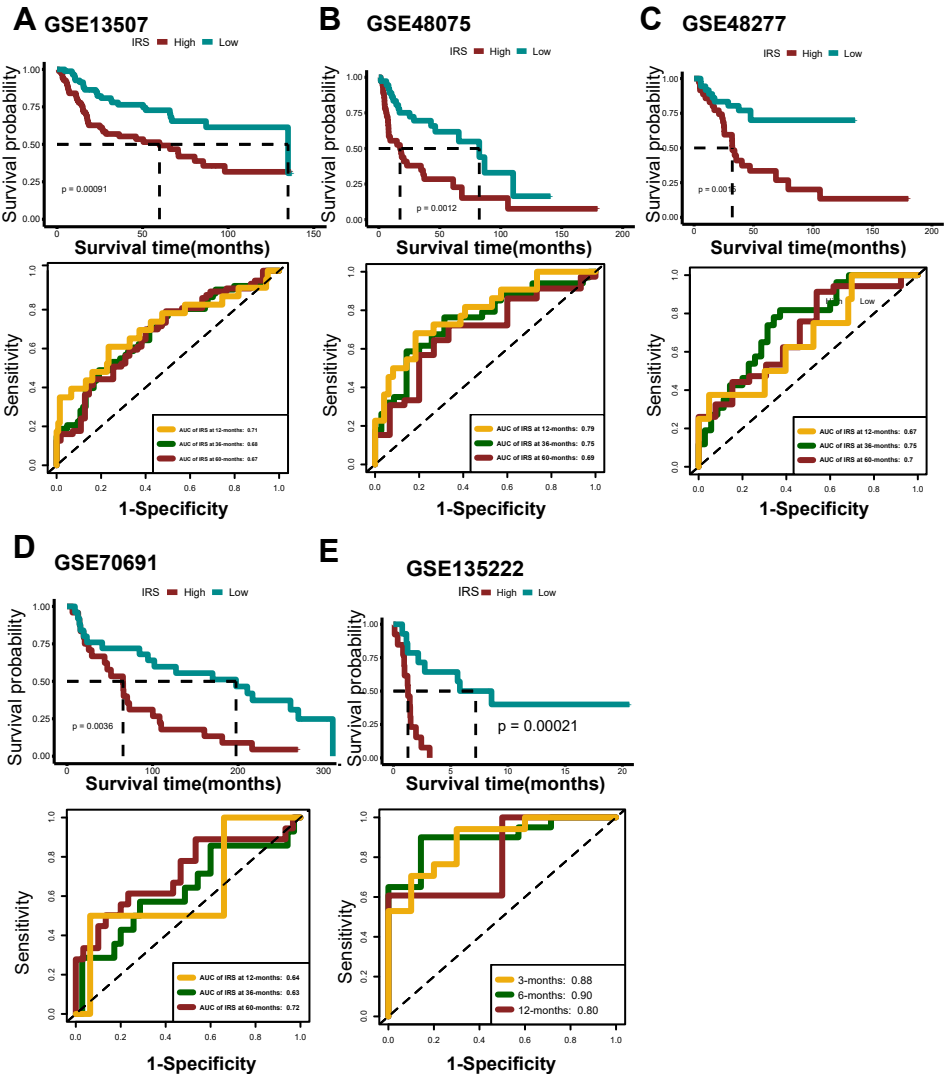

Supplement: Supplementary file 3 — Supplementary figures 20-33. [file thnov11p3089s3.zip › Figure S26.pdf]

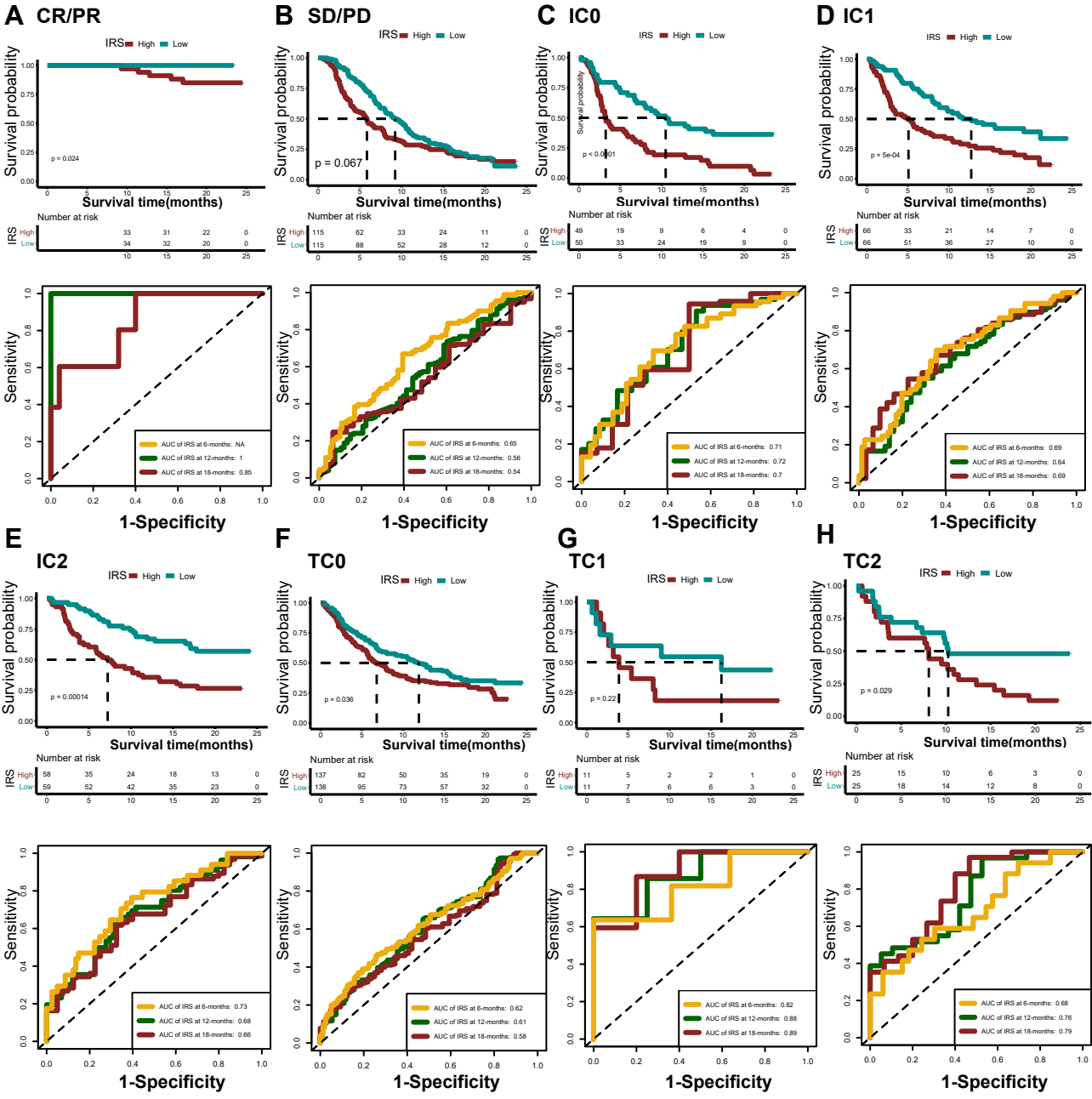

Supplement: Supplementary file 3 — Supplementary figures 20-33. [file thnov11p3089s3.zip › Figure S27.pdf]

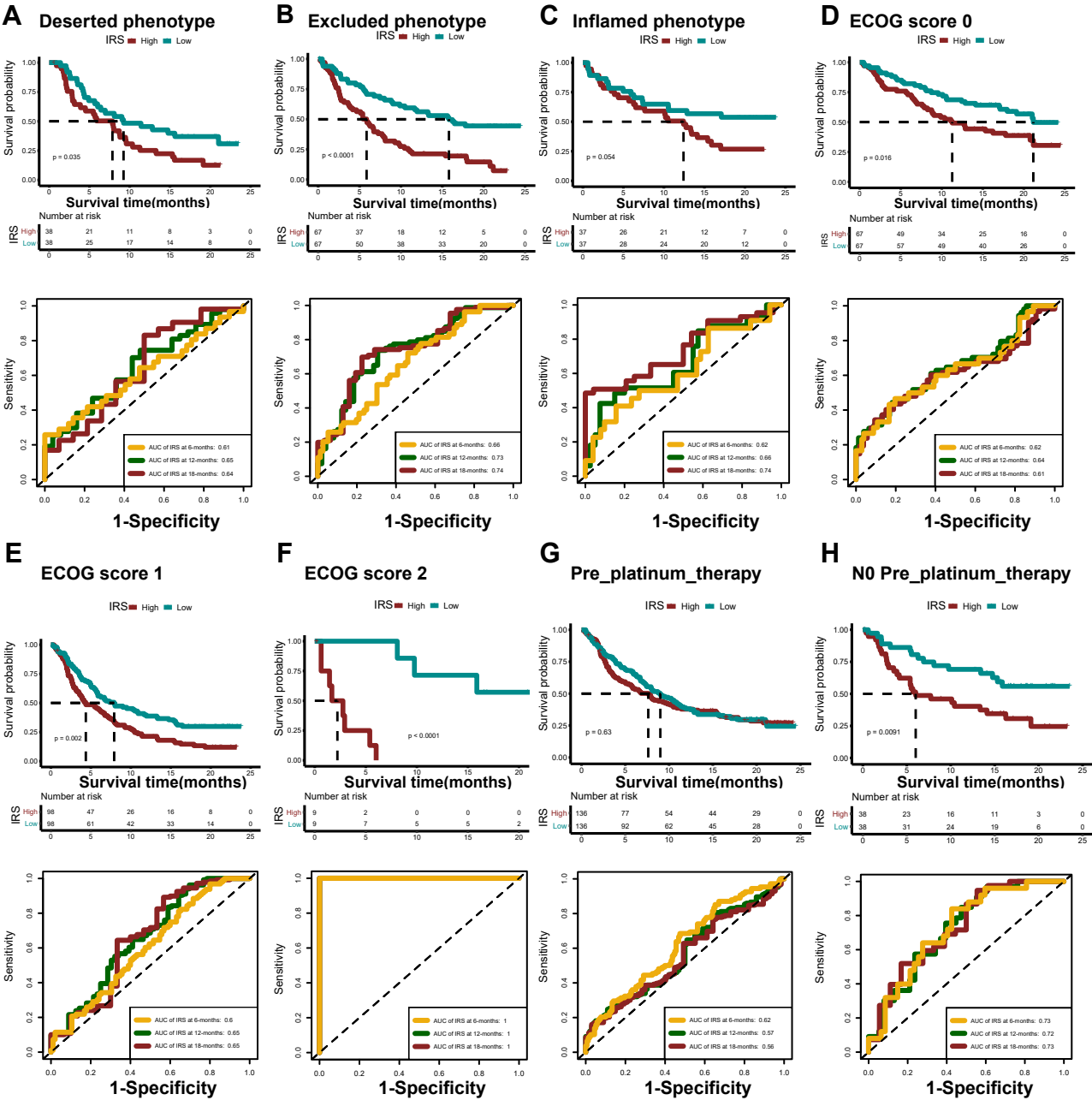

Supplement: Supplementary file 3 — Supplementary figures 20-33. [file thnov11p3089s3.zip › Figure S28.pdf]

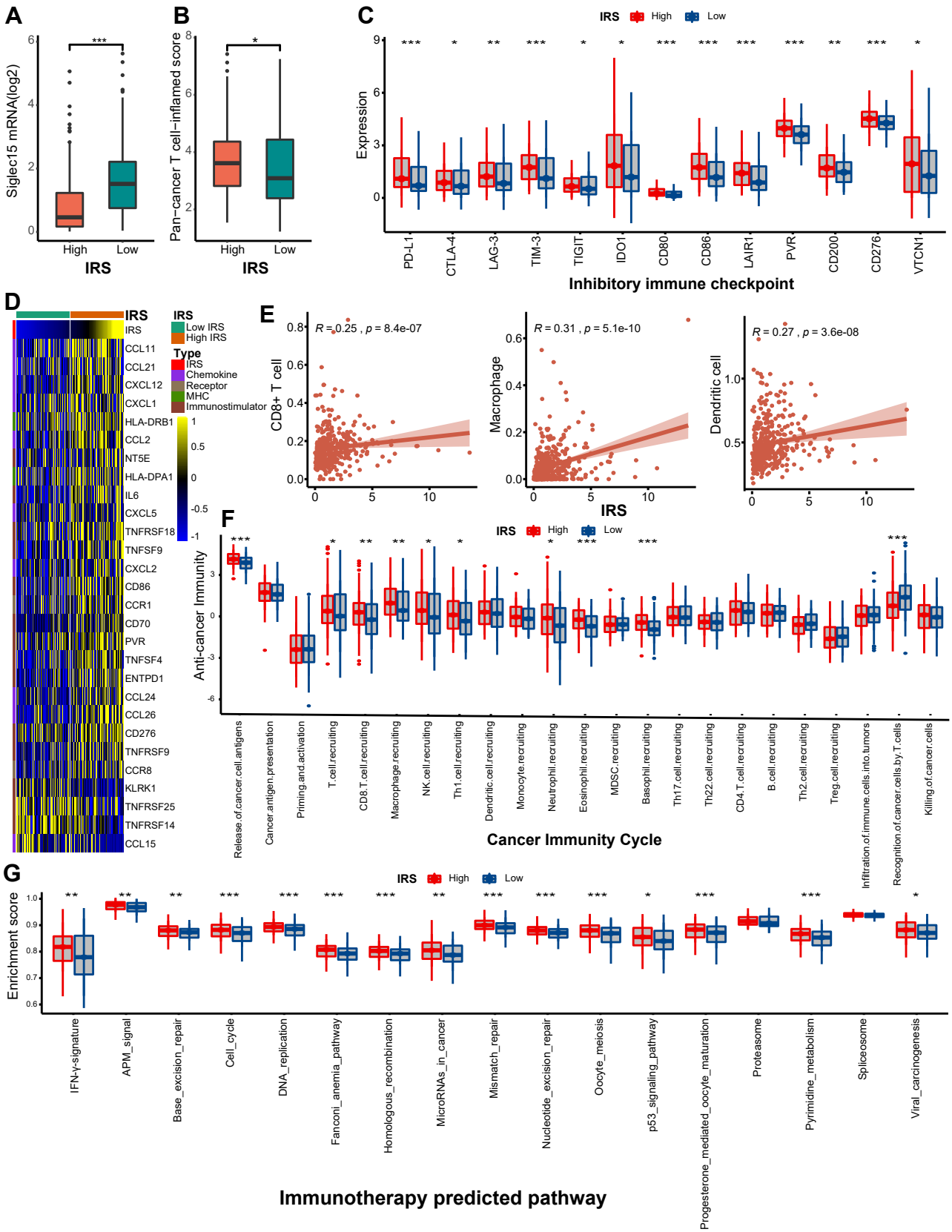

Supplement: Supplementary file 3 — Supplementary figures 20-33. [file thnov11p3089s3.zip › Figure S29.pdf]

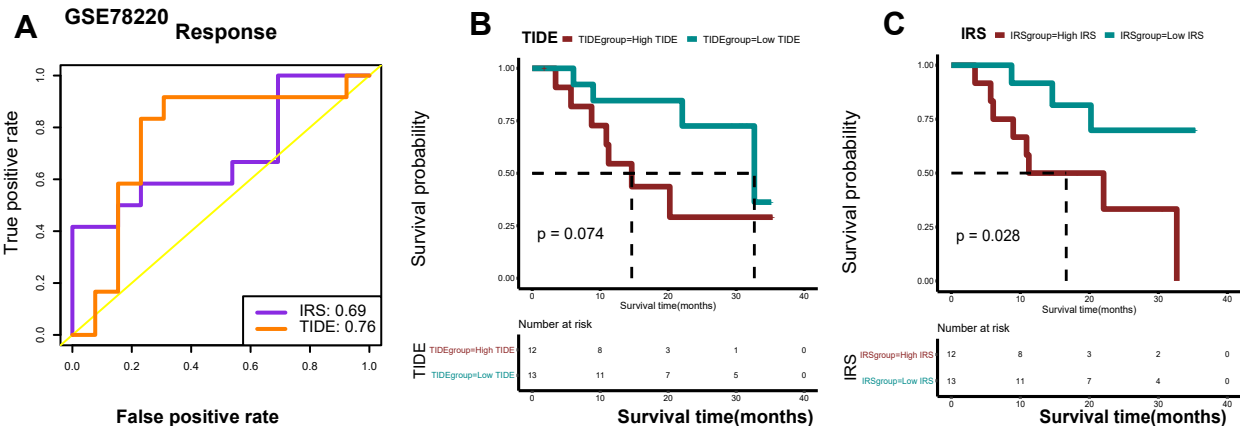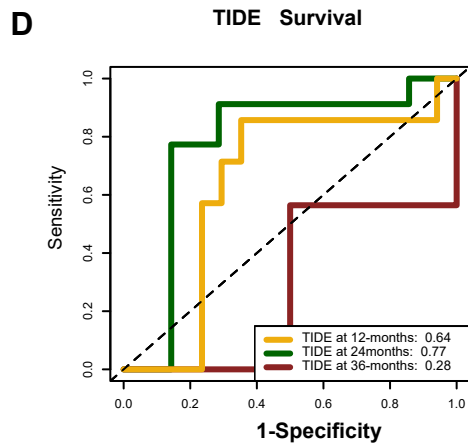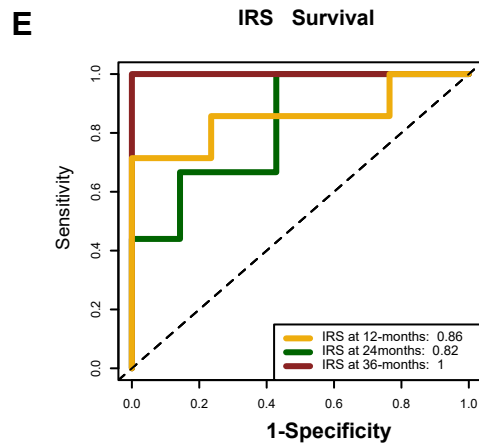

Supplement: Supplementary file 3 — Supplementary figures 20-33. [file thnov11p3089s3.zip › Figure S30.pdf]

**A** GSE91061**Response**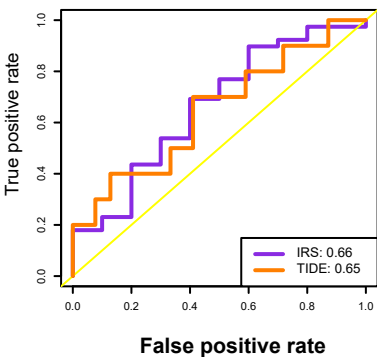**B**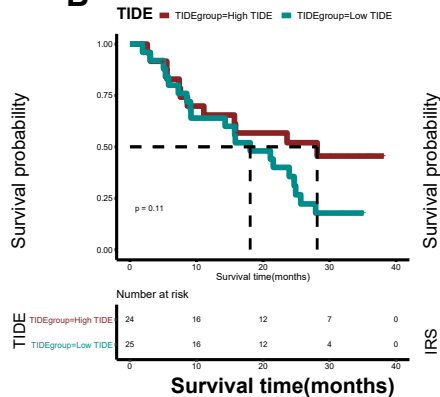**C**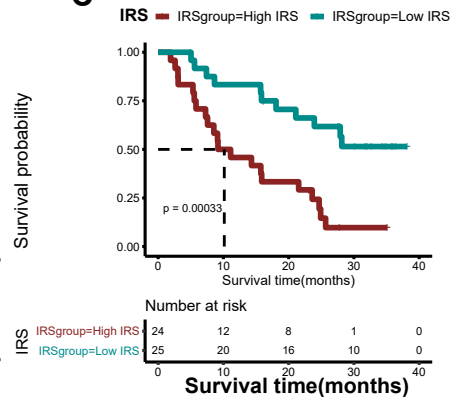**D****TIDE Survival**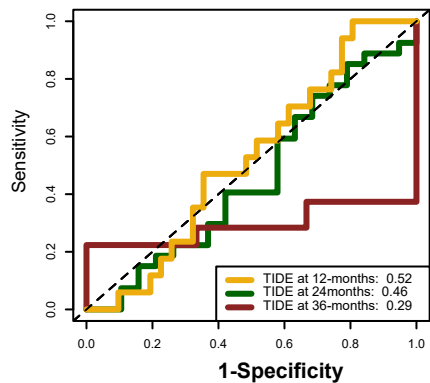**E****IRS Survival**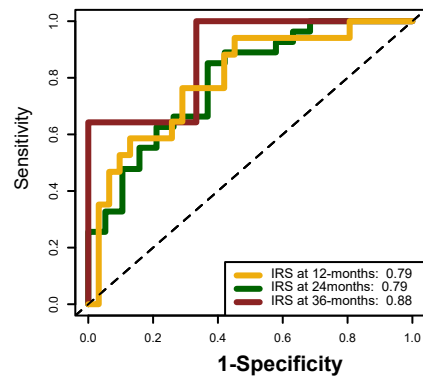

Supplement: Supplementary file 3 — Supplementary figures 20-33. [file thnov11p3089s3.zip › Figure S31.pdf]

**A**

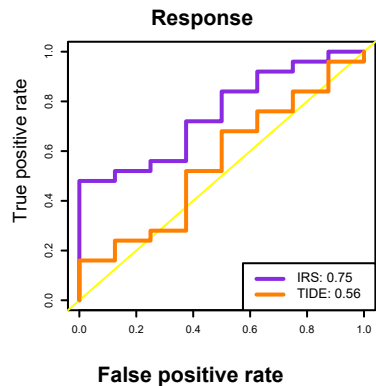

**B**

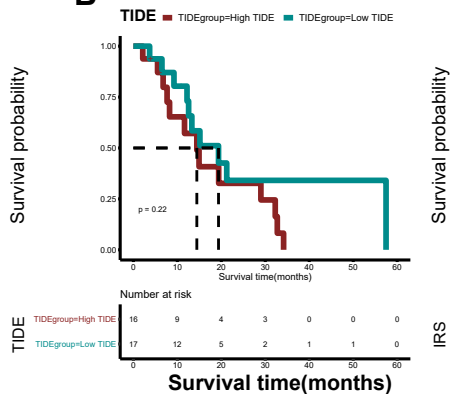

**C**

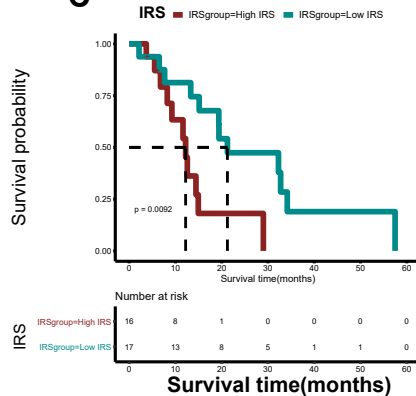

**D**

**TIDE Survival**

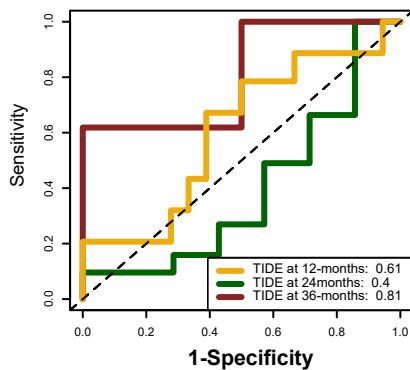

**E**

**IRS Survival**

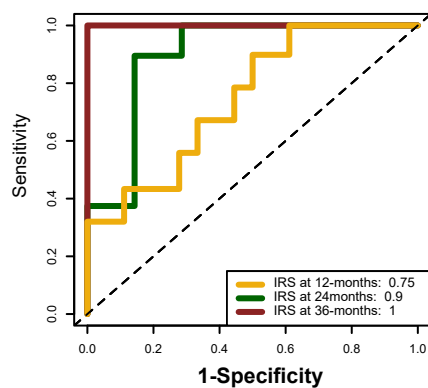

Supplement: Supplementary file 3 — Supplementary figures 20-33. [file thnov11p3089s3.zip › Figure S32.pdf]

**A** IMvigor210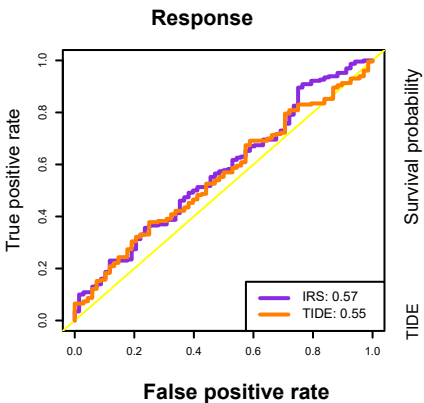**B**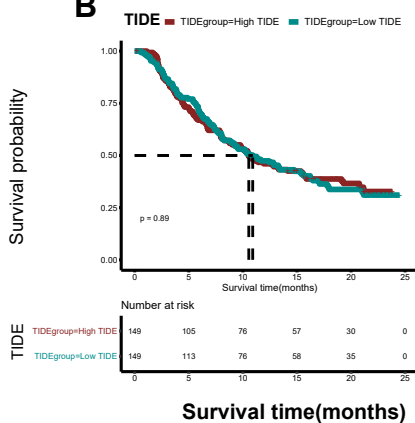**C**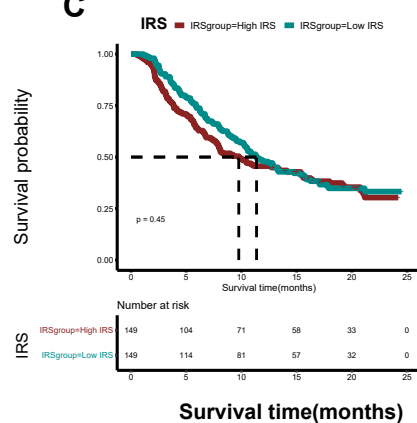**D**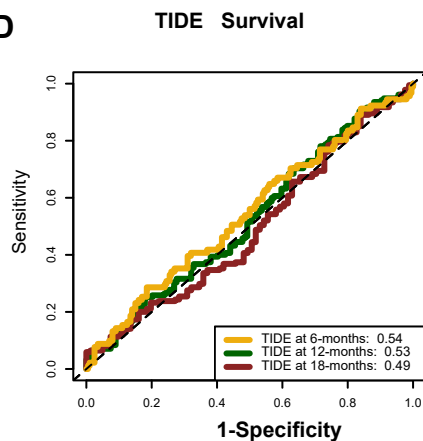**E**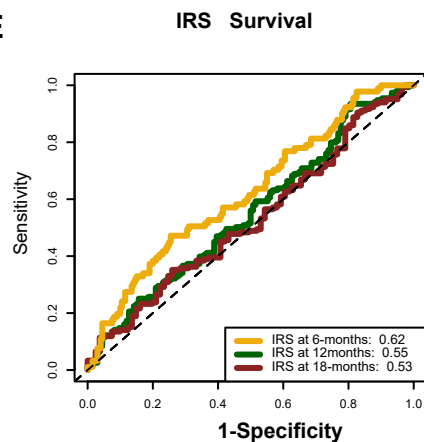

Supplement: Supplementary file 3 — Supplementary figures 20-33. [file thnov11p3089s3.zip › Figure S33.pdf]
