# Supplementary material for: Siglec15 shapes a non-inflamed tumor microenvironment and predicts the molecular subtype in bladder cancer
Source: Theranostics. 2021 Jan 1;11(7):3089–108. doi: 10.7150/thno.53649 (PMC7847675; doi:10.7150/thno.53649)
Supplement: Supplementary file 2 — Supplementary figures 1-19. [file Figure S14.pdf › Figure S14.pdf]

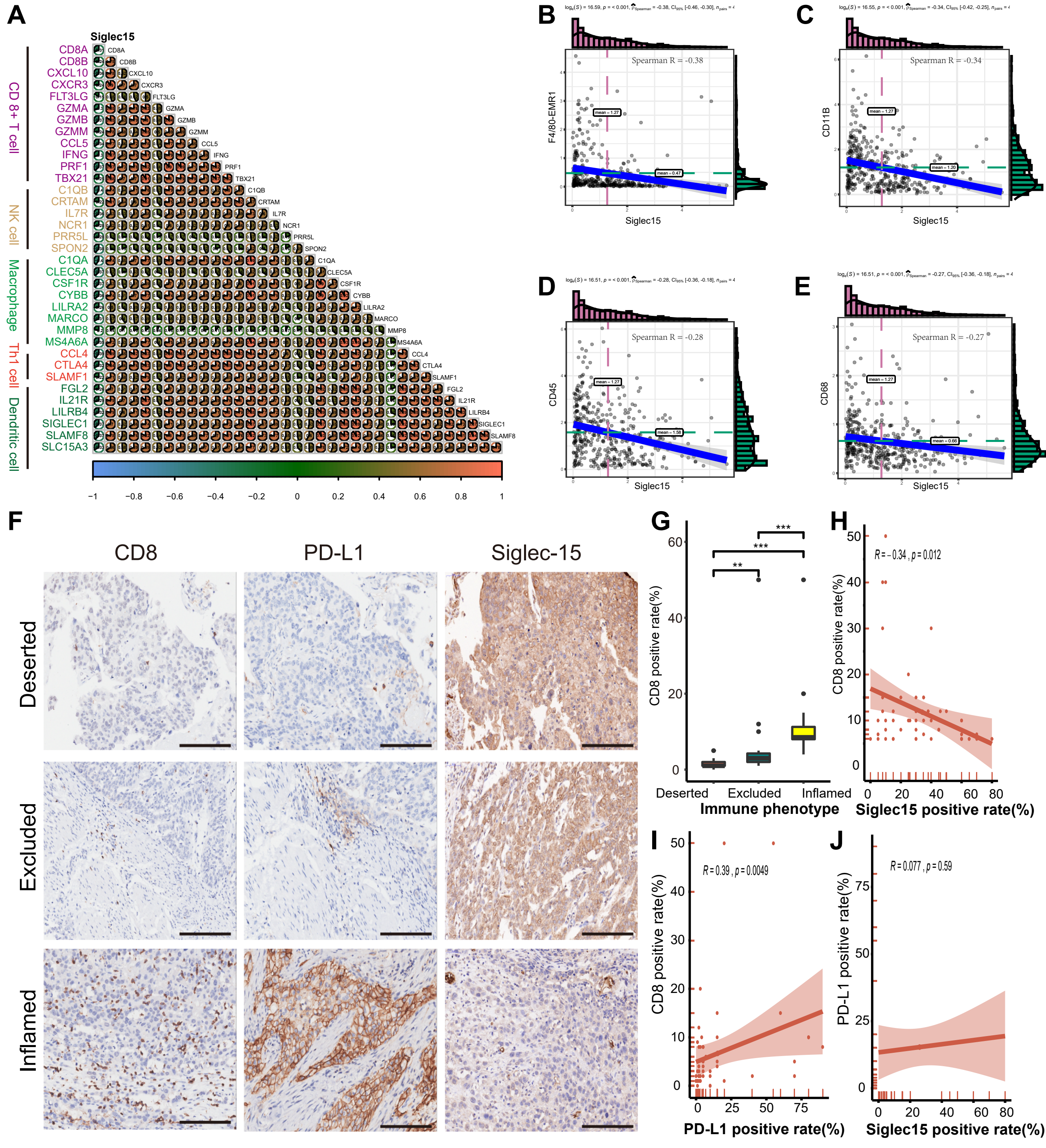

A
Siglec15
CD 8+ T cell
NK cell
Macrophage
Th1 cell
Dendritic cell
CD8A
CD8B
CXCL10
CXCR3
FLT3LG
GZMA
GZMB
GZMM
CCL5
IFNG
PRF1
TBX21
C1QB
CRTAM
IL7R
NCR1
PRR5L
SPON2
C1QA
CLEC5A
CSF1R
CYBB
LILRA2
MARCO
MMP8
MS4A6A
CCL4
CTLA4
SLAMF1
FGL2
IL21R
LILRB4
SIGLEC1
SLAMF8
SLC15A3
−1 −0.8 −0.6 −0.4 −0.2 0 0.2 0.4 0.6 0.8 1
B
Spearman R = -0.38
F4/80-EMR1
Siglec15
C
Spearman R = -0.34
CD11B
Siglec15
D
Spearman R = -0.28
CD45
Siglec15
E
Spearman R = -0.27
CD68
Siglec15
F
CD8
PD-L1
Siglec-15
Deserted
Excluded
Inflamed
G
CD8 positive rate(%)
Deserted
Excluded
Inflamed
Immune phenotype
H
R = −0.34 , p = 0.012
CD8 positive rate(%)
Siglec15 positive rate(%)
I
R = 0.39 , p = 0.0049
CD8 positive rate(%)
PD-L1 positive rate(%)
J
R = 0.077 , p = 0.59
PD-L1 positive rate(%)
Siglec15 positive rate(%)
