# Supplementary material for: Siglec15 shapes a non-inflamed tumor microenvironment and predicts the molecular subtype in bladder cancer
Source: Theranostics. 2021 Jan 1;11(7):3089–108. doi: 10.7150/thno.53649 (PMC7847675; doi:10.7150/thno.53649)
Supplement: Supplementary file 3 — Supplementary figures 20-33. [file Figure S20.pdf › Figure S20.pdf]

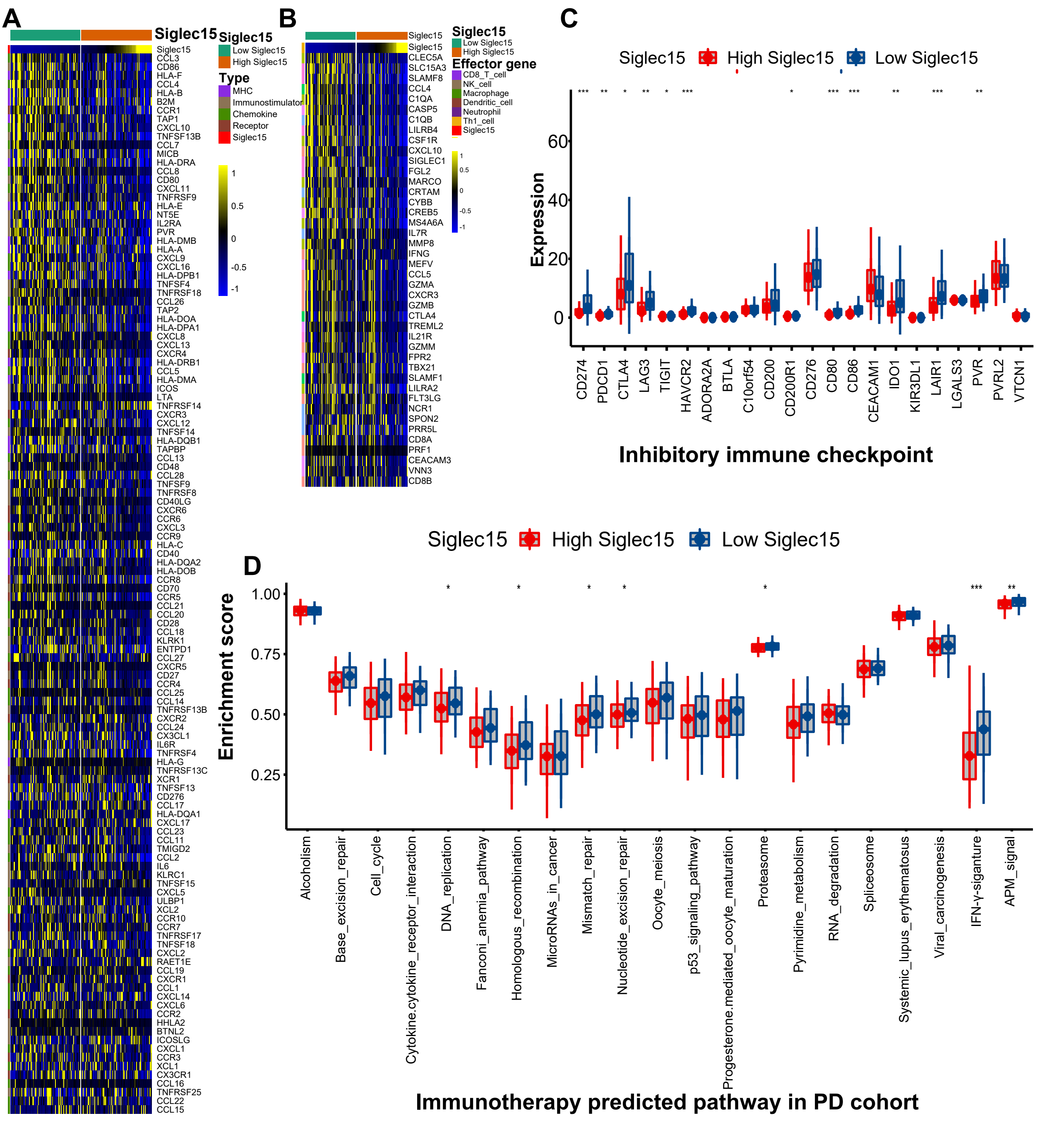
A
Siglec15
Siglec15
Low Siglec15
High Siglec15
Type
MHC
Immunostimulator
Chemokine
Receptor
Siglec15
1
0.5
0
-0.5
-1
Siglec15
CCL3
CD86
HLA-F
CCL4
HLA-B
B2M
CCR1
TAP1
CXCL10
TNFSF13B
CCL7
MICB
HLA-DRA
CCL8
CD80
CXCL11
TNFRSF9
HLA-E
NT5E
IL2RA
PVR
HLA-DMB
HLA-A
CXCL9
CXCL16
HLA-DPB1
TNFSF4
TNFRSF18
CCL26
TAP2
HLA-DOA
HLA-DPA1
CXCL8
CXCL13
CXCR4
HLA-DRB1
CCL5
HLA-DMA
ICOS
LTA
TNFRSF14
CXCR3
CXCL12
TNFSF14
HLA-DQB1
TAPBP
CCL13
CD48
CCL28
TNFSF9
TNFRSF8
CD40LG
CXCR6
CCR6
CXCL3
CCR9
HLA-C
CD40
HLA-DQA2
HLA-DOB
CCR8
CD70
CCR5
CCL21
CCL20
CD28
CCL18
KLRK1
ENTPD1
CCL27
CXCR5
CD27
CCR4
CCL25
CCL14
TNFRSF13B
CXCR2
CCL24
CX3CL1
IL6R
TNFRSF4
HLA-G
TNFRSF13C
XCR1
TNFSF13
CD276
CCL17
HLA-DQA1
CXCL17
CCL23
CCL11
TMIGD2
CCL2
IL6
KLRC1
TNFSF15
CXCL5
ULBP1
XCL2
CCR10
CCR7
TNFRSF17
TNFSF18
CXCL2
RAET1E
CCL19
CXCR1
CCL1
CXCL14
CXCL6
CCR2
HHLA2
BTNL2
ICOSLG
CXCL1
CCR3
XCL1
CX3CR1
CCL16
TNFRSF25
CCL22
CCL15
B
Siglec15
Low Siglec15
High Siglec15
Effector gene
CD8_T_cell
NK_cell
Macrophage
Dendritic_cell
Neutrophil
Th1_cell
Siglec15
1
0.5
0
-0.5
-1
Siglec15
Siglec15
CLEC5A
SLC15A3
SLAMF8
CCL4
C1QA
CASP5
C1QB
LILRB4
CSF1R
CXCL10
SIGLEC1
FGL2
MARCO
CRTAM
CYBB
CREB5
MS4A6A
IL7R
MMP8
IFNG
MEFV
CCL5
GZMA
CXCR3
GZMB
CTLA4
TREML2
IL21R
GZMM
FPR2
TBX21
SLAMF1
LILRA2
FLT3LG
NCR1
SPON2
PRR5L
CD8A
PRF1
CEACAM3
VNN3
CD8B
C
Siglec15 High Siglec15 Low Siglec15
Expression
60
40
20
0
*** ** * ** * *** * *** *** ** *** **
CD274
PDCD1
CTLA4
LAG3
TIGIT
HAVCR2
ADORA2A
BTLA
C10orf54
CD200
CD200R1
CD276
CD80
CD86
CEACAM1
IDO1
KIR3DL1
LAIR1
LGALS3
PVR
PVRL2
VTCN1
Inhibitory immune checkpoint
Siglec15 High Siglec15 Low Siglec15
D
Enrichment score
1.00
0.75
0.50
0.25
* * * * * *** **
Alcoholism
Base_excision_repair
Cell_cycle
Cytokine.cytokine_receptor_interaction
DNA_replication
Fanconi_anemia_pathway
Homologous_recombination
MicroRNAs_in_cancer
Mismatch_repair
Nucleotide_excision_repair
Oocyte_meiosis
p53_signaling_pathway
Progesterone.mediated_oocyte_maturation
Proteasome
Pyrimidine_metabolism
RNA_degradation
Spliceosome
Systemic_lupus_erythematosus
Viral_carcinogenesis
IFN-γ-signature
APM_signal
Immunotherapy predicted pathway in PD cohort
